# Design, Synthesis, and Antitumor Potential of New Thiazole-contained 5-Fluoro-2-Oxindole Derivatives as Sunitinib Analogues

**DOI:** 10.2174/0109298673346427241016100726

**Published:** 2024-11-04

**Authors:** Ivan Semenyuta, Oleksandr Los, Vitalii Sinenko, Victor Zhirnov, Lyudmyla Potikha, Oleksandr Kobzar, Volodymyr Brovarets

**Affiliations:** 1 Department of Chemistry of Natural Compounds, V. P. Kukhar Institute of Bioorganic Chemistry and Petrochemistry, Kyiv, 02094, Ukraine;; 2 Department of Chemistry of Bioactive Nitrogen-containing Heterocyclic Bases, V. P. Kukhar Institute of Bioorganic Chemistry and Petrochemistry, Kyiv, 02094, Ukraine;; 3 Department of Chemistry, Taras Shevchenko National University, Kyiv, 01601, Ukraine;; 4 Department of Mechanisms of Bioorganic Reactions, V. P. Kukhar Institute of Bioorganic Chemistry and Petrochemistry, Kyiv, 02094, Ukraine

**Keywords:** 5-Fluoro-2-oxindole, anticancer agents, thiazole, VEGFR2, sunitinib analogues, cancer

## Abstract

**Background:**

Indole is considered the most promising scaffold for anticancer drug design due to its high bioavailability, unique chemical properties, and broad spectrum of pharmacological action.

**Objective:**

Twelve novel thiazole-containing the 5-fluoro-1,3-dihydro-2H-indol-2-one derivatives as sunitinib analogs were designed and synthesized, and their anticancer activity was evaluated against the NCI-60 cancer cell lines.

**Methods:**

The thiazole-contained 5-fluoro-1,3-dihydro-2H-indol-2-one derivatives were synthesized using Knoevenagel condensation of 1,3-thiazole-5-carboxylic acid **1**. Their anticancer activities were evaluated by NCI-60 one-dose screen assay. The molecular docking studies were performed using AutoDock tools and the AutoDock Vina programs. The ADMETlab 2.0 web server predicted the physicochemical properties of compounds.

**Results:**

Among the synthesized new 5-fluoro-2-oxindole derivatives, compound **3g** demonstrated high antitumor activity (GI>70%) against eight types of cancer: leukemia, breast cancer, ovarian cancer, lung cancer, melanoma, CNS cancer, renal cancer, and colon cancer. The most activity was observed against breast cancer (T-47D, GI=96.17%), lung cancer (HOP-92, GI=95.95%), ovarian cancer (NCI/ADR-RES, GI=95.13%), and CNS cancer (SNB-75, GI=89.91%). The molecular docking results of compound **3g** demonstrated the possibility of inhibiting VEGF2 receptors as his potential anticancer mechanism. The physicochemical properties predicted for compounds **3f** and **3g** showed positive results.

**Conclusion:**

Compound 3g demonstrated high *in vitro* NCI-60 anticancer activity against nine cancer types and showed cell growth inhibition against leukemia, CNS, and breast cancer at 6 - 31% higher than Sunitinib, and may represent the basis for further modification of the thiazole-containing analogs of the anticancer drug Sunitinib.

## 1 INTRODUCTION

**Fig. (1):**
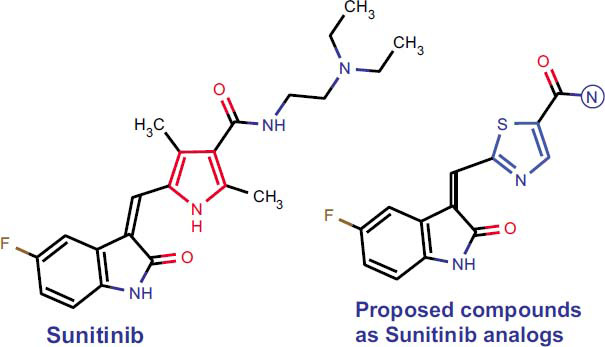
Design of thiazole-contained derivatives of sunitinib.

The nitrogen-containing heterocycles are regarded as the most important scaffolds in drug development [1]. Among them, the indole moiety is the most frequently occurring nitrogen heterocycle in biologically active compounds. Indole is widely considered the most promising scaffold for anticancer drug design due to its high bioavailability, unique chemical properties, and broad spectrum of pharmacological actions [2-6]. Known indole derivatives exhibit anticancer properties by acting on a variety of molecular targets, including the tumor suppressor protein p53, the Bcl-2 family member Mcl-1, tubulin assembly enzymes, Pim-kinases, tyrosine kinases, aromatase, histone deacetylases, topoisomerases, and the G protein-coupled estrogen and sigma receptors. Inhibition of these targets leads to cell cycle arrest and induction of cancer cell apoptosis [7-9]. Most indole-contained compounds are multitarget agents that inhibit cancer cell growth through different mechanisms. It is one of the advantages of indols in treating multifactorial diseases to which cancer belongs [10, 11]. So, the anticancer drug sunitinib is an oxindole derivative and is a multitarget inhibitor of receptor tyrosine kinases. It has been found that sunitinib terminates cellular signaling by inhibiting platelet-derived growth factor receptors (PDGFRa and PDGFRb), vascular endothelial growth factor receptors (VEGFR1, VEGFR2, and VEGFR3), stem cell factor receptors (KIT), Fms-like tyrosine kinase-3 (FLT3), colony-stimulating factor receptor Type 1 (CSF-1R), and the glial cell-line neurotrophic factor receptor (RET), as demonstrated in biochemical and cell proliferation assays [12]. Therefore, developing the oxindole fragment as a matrix for creating new drugs with antitumor activity is relevant. Besides, a study [13] reported that indole-thiazole derivatives are selective agonists of the G protein-coupled estrogen receptor (but not the classical estrogen receptors α and β), representing a promising target for treating ER-negative breast cancers. 4-Oxo-thiazol-2-dihydropyrazole hybrids and thiazolidinone derivatives of oxindole have been found to possess antiproliferative activity *in vitro* in various human cell lines [14-16]. We previously synthesized thiazol- contained 5-sulfanyl substituted (thiazol-4-yl) phosphonium salt derivatives and thiazole-containing derivatives of rhodanine, which showed high anticancer activity *in vitro* [17, 18]. Wherein compound **7** [17] demonstrated the best anti-cancer results against leukemia, ovarian cancer, breast cancer, melanoma, and colon cancer. Also, compound **2** showed high activity (GI_50_=0.77 - 1.48 µM) against most NCI-60 cancer cells. Simultaneously, compound **2** demonstrated low-level cytotoxicity with LC_50_ ≥ 100 µM values for most cancer cell lines. Thus, the present study used sunitinib to design and synthesize derivatives with a replacement pyrrole group on the thiazole group (Fig. **1**). Twelve novel derivatives of 5-fluoro-1,3-dihydro-2H-indol-2-one were synthesized, and their anticancer activity was investigated in NCI-60 cancer cells.

## 2 MATERIALS AND METHODS

### 2.1 Chemistry

We used commercially available chemical reagents (Enamine Ltd.) and solvents without purifying them. To monitor the progress of reactions, we employed the TCL method. The ^1^H and ^13^C NMR spectra were recorded on a Varian Mercury 300, Varian Unity Plus 400, Bruker 170 Avance 500, and Agilent ProPulse 600 spectrometers. IR spectra of compounds were recorded on a Bruker Vertex 70 infrared spectrometer using the KBr pellet method. LCMS spectra were obtained using an Agilent 1100 Series Liquid Chromatograph (HPLC system). The MPA100 OptiMelt automated system was employed on the melting point. The supplementary materials describe the chemical synthesis methods of compounds and their spectral data in detail.

### 2.2 Biology

#### 2.2.1 NCI-60 *in vitro* Anticancer Screening of Compounds (One Dose Assay)

Twelve synthesized compounds were studied at the National Cancer Institute (NCI, Bethesda, Maryland, U.S.A.) for their anticancer activity as part of the Developmental Therapeutic Program (DTP). The NCI cell line panel included 58 human tumor cell lines (except SNB-19 and TK-10) related to nine cancer types: lung, colon, melanoma, renal, ovarian, brain, leukemia, breast, and prostate [19]. The primary anticancer screening was started by cell inoculation of each 58- cell line into a series of standard 96-well microliter plates at 5000-40000 cells/well in RPMI 1640 medium, containing 5% fetal bovine serum and 2 mM L-glutamine (day 0), and then preincubated without drug at 37°C and 5% CO_2_ for 24 h. Studied compounds were added (10^-5^ M) (day 1), followed by incubation for 48 h. Next, the media were removed, and the cells were fixed *in situ*, washed, and dried (day 3). The cell density determination was based on measuring cellular protein content using the sulforhodamine B assay. After incubation, cell monolayers were fixed with 10% trichloroacetic acid and stained for 30 min. The excess dye was removed by washing repeatedly with 1% acetic acid. The bound stain was resolved in a 10 mM Tris base and measured spectrophotometrically on automated microplate readers (510 nm) [20]. Our biological research of the compounds was conducted within the NCI Development Therapeutics Program (DTP) framework. The NCI Development Therapeutics Program (DTP) provides services and resources to the academic and private-sector research communities worldwide to facilitate the discovery and development of new cancer therapeutic agents.

### 2.3 Molecular Docking Studies

ChemAxon MarvinSketch 5.3.7 [21] program was used to create, pre-optimize, and save the ligand structures in mol2 format. The Avogadro v1.2.0 program (for Windows) [22] was employed to minimize the ligands' potential energy (MMFF94s force field) with the steepest descent algorithm. The AutoDock Tools (ADT) 1.5.6 [23] software was applied to prepare the receptors and ligands; all polar hydrogens were added to the receptor structures, and all receptor atoms were renumbered using the noBondOrder method; the calculated partial charges of the ligands and receptors were changed by ADT (Gasteiger method). The refined proteins and ligands were saved in pdbqt format. AutoDock Vina 1.1.2 program [24] for docking studies and the grid map of 30*30*30 A with a grid spacing of 1Å was used. The research and demonstration of receptor-ligand interactions were performed by Accelrys Discovery Studio Visualizer 4.0.100 [25].

### 2.4 Prediction of Physicochemical Properties

The ADMETlab 2.0 Web server [26] was used to predict the physicochemical properties of compounds **3f** and **3g** compared to sunitinib. This web platform for predicting the ADMET properties of new chemical compounds is based on robust QSAR models and a chemical compound database with 288,967 entries.

## 3 RESULTS AND DISCUSSION

### 3.1 Chemistry

**Scheme 1:**
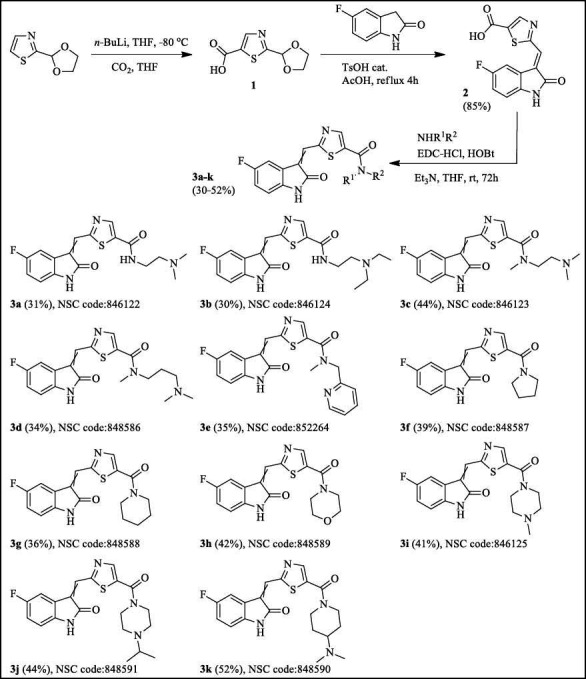
Synthesis of target 2-[(5-fluoro-2-oxo-1,2-dihydro-3H-indol-3-ylidene)methyl]-1,3-thiazole-5-carboxamides.

Scheme **1** presents the synthesis of thiazole-contained compounds **3a-3k** based on the condensation of thiazole-2-carbaldehyde. A new efficient method for synthesizing linked 3-(1,3-thiazol-2-ylmethylidene)-1,3-dihydro-2H-indol-2-ones has been developed, which involves combining 5-fluoro-1,3-dihydro-2H-indol-2-one with 2-(1,3-dioxolan-2-yl)-1,3-thiazole-5-carboxylic acid (1) in the presence of p-toluenesulfonic acid and acetic acid as solvent by employing the versatile Knoevenagel condensation reaction (Supplementary material).

**Fig. (2):**
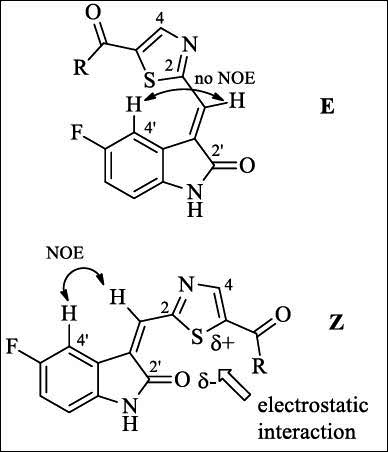
Two configurations of 2-[(5-fluoro-2-oxo-1,2-dihydro-3H-indol-3-ylidene)methyl]-1,3-thiazole-5-carboxylic acid derivatives.

The starting 1,3-thiazole-5-carboxylic acid **1** [27] was obtained by the carboxylation reaction of the appropriate 2-(1,3-dioxolan-2-yl)thiazole, as shown in Scheme **1**. However, like other known 1,3-dioxolan-2-yl substituted 1,3-thiazoles, acid 1 was not used to synthesize Knoevenagel condensation products. The reaction product 2-[(E)-(5-fluoro-2-oxo-1,2-dihydro-3H-indol-3-ylidene)methyl]-1,3-thiazole-5-carboxylic acid **2** was obtained in a high yield, and its structure was confirmed by the results of correlation heteroatom 2D NMR spectroscopy (Fig. (**S1-S57**) and 1D NOE analysis (Fig. **S5** and Fig. **2**).

The 3-(thiazol-2-ylmethylene)indolin-2-ones may exist as either the Z or E isomers (Fig. **2**). The Z-configured compounds should exhibit a NOE effect between the proton at position 4 and the vinyl proton. In contrast, the E-configured compounds should not display this effect. Compound **2** showed no NOE effect between the proton at position 4 and the vinyl proton, which indicated that **2** may exist as the E isomer. According to the literature [28], the Z-E configuration of 3-heteroaromatic substituted indolin-2-ones was related to the electrostatic effect or hydrogen-bond interaction. Thus, 3-(furanyl-2-ylmethylene)indolin-2-ones appear to favor the E isomer form, but 3-(pyrazolyl-2-ylmethylene)indolin-2-ones prefer to exist as the Z isomer form. The chemical shifts for the proton Н4 of the 3-heteroaromatic substituted indolin-2-ones for the E isomer are significantly greater than those for the Z isomer. It may be the result of the deshielding effect of heteroaromatic rings. The formation of intramolecular hydrogen bonding between the proton Н4 of the indolin-2-one ring and the N3 atom in the thiazole ring and the deshielding effect of the thiazole ring may cause the chemical shifts for the proton Н4 of 3-(thiazol-2-ylmethylene)indolin-2-ones to appear at a relatively low field. Therefore, taking the NOE analysis and the chemical shift (δ=8.89 ppm) for the proton Н4 of compound **2** into consideration, it would appear that 3-(thiazol-2-ylmethylene)indolin-2-one **2** exists as the E isomer. Further acylation of primary and secondary amines with acid **2** was carried out in THF at room temperature in the presence of 1-ethyl-3-(3-dimethylaminopropyl)carbodiimide, hydrochloride hydroxybenzotriazole, and triethylamine (Scheme **1**). The substituents of the target compounds are mentioned in Table **S1**. The obtained carboxamides **3a-k** turned out to be mixtures of E and Z isomers where the content of the E isomer as the main component was 71-87% according to the data of their ^1^H NMR spectra. At the same time, the content of the Z isomeric form in compounds **3a-d** with a non-cyclic aliphatic residue in the amide fragment was slightly higher (23-29%) compared to the derivatives of cyclic amines **3f-k** and *N*-methyl-1-(pyridin-2-yl)methanamine **3e** where the Z isomer was 15-20%.

The proton signals of the 3-(thiazol-2-ylmethylene)indolin-2-one core of the main component of the mixture in the ^1^H NMR spectra of amides 3 were observed in the same regions that determined for the E isomer of the compound 2, except for the regular shift of the H4 signal of thiazole cycle in a strong field by 0.1-0.2 ppm. The most noticeable changes in the position of the signals of the Z isomeric form of amides 3 corresponded to the protons of the methylidene group (shift to the weak field, Δδ = +0.5 ppm) and H4' (shift to the strong field, Δδ = -1.1 ppm) of indolinone fragment. Such differences in the signals of these protons can be explained by the fact that there is no deshielding of H4' by the thiazole cycle in the Z-isomeric form. Instead, the methylidene proton undergoes additional deshielding due to convergence with the benzene ring of indolinone. Noticeable changes in the values of the chemical shifts of NH protons (shift to a weak field, Δδ = +0.2 ppm) and H4 of the thiazole ring (shift to a strong field, Δδ = -0.2 ppm) in the Z isomeric form are probably due to the electrostatic interaction between the carbonyl oxygen of C2' and partial positively charged S1 thiazole (Fig. **2**). This type of interaction is generally responsible for increasing the stability of the Z isomeric form of amides 3 in comparison with the known representatives of the series of 3-(thiazol-2-ylmethylene)indolin-2-ones [29, 30] and 3-(benzothiazol-2-ylmethylene)indolin-2-ones [31, 32], which do not contain electron-withdrawing substituents in the thiazole ring and form exclusively E isomers. In the case of acid 2, an easily deprotonated group also does not contribute to strengthening the interaction between S1 of the thiazole and the C2' carbonyl oxygen. The ^13^C NMR spectra of amides 3 are characterized by a complex pattern of two sets of signals of both isomeric forms for most carbon atoms, except for the amine residues of the amide group. The signals of the E isomer can be easily identified and determined by the intensity and the chemical shift value, which is slightly different from the parent acid 2. Three strong bands are present in the IR spectra of amides 3: ν_C=O_ 1691-1712 cm^-1^, ν_C=O_ 1552-1626 cm^-1^ (broad), and 1466-1484 cm^-1^.

Bands of valence vibrations of carbonyl groups naturally shifted to the low-frequency region in comparison with acid **2** (ν_C=O_ 1724 (strong) cm^-1^ and 1681 (medium) cm^-1^). 3-(Thiazol-2-ylmethylene)indolin-2-one derivatives **2** and **3a-k** are red solids. According to their UV/Vis spectra, the structure of the substituent at C5 of the thiazole ring and the presence of Z isomeric forms in product **3** have a relatively small effect on the shape of the absorption curves. In particular, the absorption band of low intensity (ε 3100-4600) is present in the low-frequency part of the visible region of the UV/Vis spectra of compounds **2** and **3** in DMSO. This band (λ_max_ = 429-438 nm) for amides **3** undergoes a hypsochromic shift by 2-11 nm relative to the corresponding band for acid **2** (λ_max_ = 440 nm). A slight bathochromic shift of 1-7 nm relative to the maximum for acid **2** (λ_max_ = 357 nm) was observed for the group of intense absorption bands with a maximum in the region of 358-364 nm (ε 16200-24900).

### 3.2 Biology

#### 3.2.1 NCI-60 *In Vitro* Anticancer Screening of Compounds (One Dose Assay)

Table **1** presents the statistical analysis results of the effect of 5-fluoro-1,3-dihydro-2H-indol-2-one derivatives on cell growth of the NCI-60 total panel and subpanels obtained in the one-dose assay. The subpanels have formed the following series based on the average inhibitory activity of compound **3g**: leukemia (76%) > breast cancer (63%) > ovarian cancer (58) > NSCLC (54) > prostate cancer (48) ≈ colon cancer (47) ≈ melanoma (46) > renal cancer (41) > CNS cancer (27). The sensitivity of the leukemia subpanel to compound **3g** differed statistically significantly (*p*<0.05) from the other subpanels, except breast cancer, due to a significant spread in the sensitivity to the compound **3g** of individual cell lines belonging to this subpanel (Table **S2**). Furthermore, the growth inhibitory activity of **3g** was not significantly different from that of **3f** against most subpanels, except for NSCLC (*p* = 0.03). Compound **3g** moderately reduced cell growth in the total panels and subpanels for NSCLC, CNS, ovarian, and breast cancer. It showed high inhibitory activity against the leukemic subpanel. Although **3f** showed moderate inhibitory activity only against the leukemia subpanel, it was not significantly different from **3g** (*p* = 0.06). The rest of the compounds exhibited only weak or very weak activity. Complete data from the one-dose assay are presented in Table **S2**.

 Fig. (**3**) presents a detailed inhibitory activity analysis of compounds **3f** and **3g** for all NCI-60 subpanels. So, for compound **3g**, we have formed the following activity series based on the average GI values: leukemia-76% > breast cancer - 63% > ovarian cancer - 58% > CNS cancer - 54% > lung cancer - 53% > prostate cancer - 48% ≈ colon cancer - 47% ≈ melanoma- 46% > renal cancer - 42%. For less active compound **3f**, this series is different: leukemia - 63% > breast cancer - 46% > melanoma- 41% ≈ ovarian cancer - 41% ≈ prostate cancer - 41% > renal cancer - 34% > CNS cancer - 32% > lung cancer - 21% > colon cancer - 17%.

For comparison, the anticancer activity of sunitinib is as follows: leukemia -70% > colon cancer - 69% > prostate cancer - 68% > renal cancer - 66% > melanoma - 60% > lung cancer - 56% > ovarian cancer - 55% > CNS cancer - 35% > breast cancer - 32%. Thus, compound 3g demonstrated lower average GI values (14 - 24%) for colon, melanoma, renal, and prostate cancer than sunitinib. The GI values for lung and ovarian cancers were found to be relatively equal, and for leukemia, CNS cancer, and breast cancer, they were higher by 6 - 31%.

The results of growth inhibition of some cancer cell lines NCI-60 for compound **3g** are presented in Fig. (**4**), which demonstrates inhibition activity (GI>70%) for the following cancer cell lines: leukemia - CCRF-CEM, HL-60, MOLT-4, RPMI-8226, and SR with GI=71.49 - 84.49%, breast cancer cell lines MCF7, BT-549, T-47D, MDA-MB-468 with values GI=72.44 - 96.17%, ovarian cancer cell lines OVCAR-3, OVCAR-4, and NCI/ADR-RES with GI=71.96 - 95.13%. Moreover, compound **3g** demonstrated growth inhibition in lung cancer cells HOP-92 and NCI-H522 with GI50 values of 76.72% and 95.95%, respectively, and melanoma cancer cells LOX IMVI (GI=75.21%) and MDA-MB-435 (GI=78.05%). Furthermore, compound **3g** showed growth inhibition in CNS cancer cell SNB-75 (GI=89.91%), renal cancer cell line CAKI-1 (GI=84.84%), and colon cancer line HCT-15 (GI=72.24%).

**Fig. (4):**
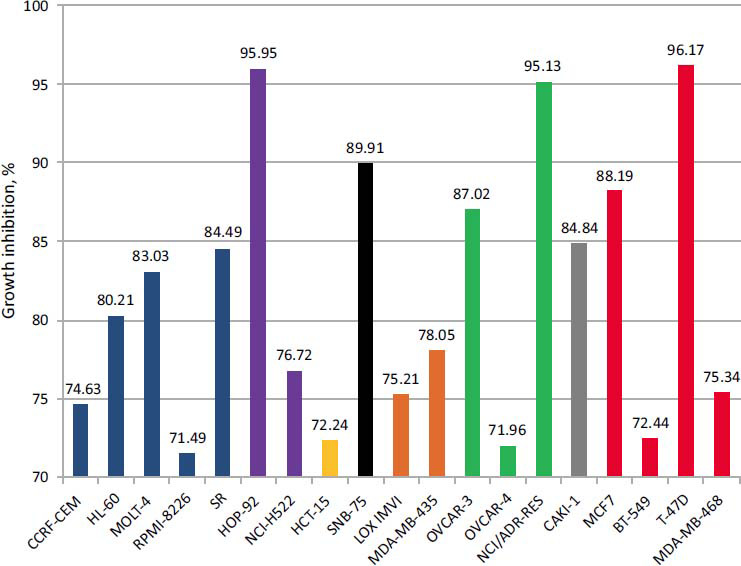
Growth inhibition in some cancer cell lines NCI-60 using compound **3g.**

Thus, compound **3g** demonstrated inhibitory activity (GI > 70%) in 8 out of 9 types of cancer, except prostate cancer (Fig. **4**). Compound **3g** exhibited the greatest activity against breast cancer (cell line T-47D) with a GI value of 96.17%, lung cancer (cell line HOP-92) with a GI value of 95.95%, and ovarian cancer (cell line NCI/ADR-RES) with a GI value of 95.13%. These results suggest a mechanism of anticancer activity similar to the sunitinib molecular. A study [33] reported promising results of targeted therapy for TNBC using tyrosine kinase inhibitors. Optimistic results were also reported using tyrosine kinase inhibitors to treat ovarian cancer [34]. This study also described promising approaches to treating non-small cell lung cancer using tyrosine kinase inhibitors [35]. The molecular docking method was used to study the mechanism of anticancer activity of compound **3g** more thoroughly.

### 3.3 Molecular Modeling

#### 3.3.1 Molecular Docking Study

Molecular docking for compounds **3f** and **3g** was conducted using the following structures of receptor tyrosine kinases (RTKs): human VEGFR1 (PDB ID: 3HNG), human VEGFR2 (PDB ID: 4AGD), human PDGFRA (alpha) (PDB ID: 6JOK), and receptor tyrosine kinase c-KIT (PDB ID: 3G0E). All structures of RTKs were received from the RCSB Protein Data Bank [36]. Table **2** demonstrates the properties of the interactions of compounds **3f** and **3g** and redocking molecules sunitinib and N-(4-Chlorophenyl)-2-((pyridin-4-ylmethyl)amino) benzamide in the ATP-binding centers of VEGFR1, VEGFR2, PDGFRA, and receptor c-KIT.

The validation molecular docking was conducted by redocking co-crystalized sunitinib and N-(4-Chlorophenyl)-2-((pyridin-4-ylmethyl)amino) benzamide into the ATP-binding sites of human VEGFR1, VEGFR2, PDGFRA, and receptor c-KIT (Table **2**). The estimated binding energy (∆G) amounted to -7.3 to -10.7 kcal/mol, and the RMSD values were 1.03 - 1.89 Ǻ. The docking results demonstrated that the compounds **3f** and **3g** formed high-energetically ligand-receptor complexes into ATP-binding center VEGFR2, with estimated binding energies ranging from - 9.9 to -10.1 kcal/mol than the more co-crystalized sunitinib (∆G= -9.0 kcal/mol).

The complexation in compounds **3f** and **3g** with other RTKs occurred with lower energy, with VEGFR1 at - 8.9 kcal/mol and PDGFRA at -7.8 and -8.3 kcal/mol, and with receptor c-KIT, the complex formation occurred with -8.5 and -8.7, kcal/mol respectively. Thus, the most energetically favorable complex formation was observed for compounds **3f** and **3g** in the ATP-binding pocket of VEGFR2 with ∆G values of -9.9 and -10.1 kcal/mol. This energy was found to be higher than the binding energy of sunitinib, *i.e.*, ∆G= -9.0 kcal/mol, and correlated to the significant experimental anticancer activity of compounds **3f** and **3g**. Fig. (**5**) shows the interaction of compounds **3g** with the ATP-binding site of the VEGFR2.

Fig. (**5A**) shows the docking results of compound 3g and demonstrates the formation of the ligand-receptor (VEGFR2) complex. The predicted binding energy of this complex was ∆G = – 10.1 kcal/mol. The complex stabilization occurred by four hydrogen bonds: between the fluorine atom and amino acid LYS868 (2.45Ǻ) and PHE1047 (2.97Ǻ); between the oxygen atom of the indole group and the amino acid residue CYS919 (1.83Ǻ), and between indole group nitrogen and amino acid GLU917 (2.08Ǻ). Besides, in the complex formation, multiple hydrophobic interactions (3.70 - 5.32Ǻ) occurred between the indole and thiazole groups and surrounding amino acids. Fig. (**5B**) demonstrates similar positions of sunitinib-ligand and compounds 3f and 3g in the ATP binding pocket of VEGFR2. Thus, the molecular docking of compounds 3f and 3g indicated a potential mechanism of the antitumor activity through complexation in the ATP-binding center VEGFR2, which was similar to sunitinib.

#### 3.3.2 Structure-activity Relationship

**Fig. (6):**
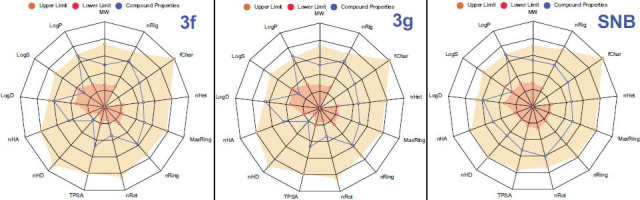
Radar charts (using ADMETlab 2.0) showing molecular physicochemical properties of compounds **3f, 3g**, and sunitinib; **3f** -compound **3f**, 3g - compound **3g**, SNB - sunitinib.

Replacing the pyrrole group with a similar thiazole fragment in sunitinib led to high-active compounds **3f** and **3g**, which contained pyrrolidine and piperidine carbonyl groups in the 5***^th^*** position of thiazole. Modifying this group on dimethylamine and diethylamine afforded low-activity compounds **3a - 3d**. Moreover, positive results did not occur with an addition at the 5***^th^*** position of the thiazole with piperidine, morpholine, methylpiperazine, and methylethylpiperazine groups, forming low-activity compounds **3d** and **3h-3j** (Fig. **6**).

#### 3.3.3 Prediction of Physicochemical Properties of Compounds

Fig. (**6**) demonstrates the ADMETlab 2.0 Web server prediction results for the molecular physicochemical properties of compounds **3f**, **3g**, and sunitinib.

Table **3** provides a detailed description of the physicochemical properties of compounds obtained in comparison to sunitinib.

Table **3** presents the main physicochemical properties of compounds **3f, 3g**, and sunitinib (for comparisons) calculated using the ADMETlab 2.0 web server. The compounds demonstrated good molecular properties, such as a number of rigid bonds (23 - 24), a formal charge of molecules (0), a number of heteroatoms (7), a number of atoms in the biggest ring (9), a number of rings (4), a number of rotatable bonds (3), and a number of hydrogen bond donors (1) and acceptors (5). The topological polar surface area of compounds **3f** and **3g** was 62.300 A^2^, which was below the value of sunitinib (77.230 A^2^) but in the optimal range of 0 - 140.0 A^2^. The synthesized compounds **3f** and **3g** were found to be moderately soluble and showed a decrease in overall aqueous solubility (logS) compared to sunitinib: Sunitinib -3.893 > -5.094 (**3f**) > -5.480 (**3g**). The lipophilicity (LogP) associated with membrane permeability of compounds is presented as 3.244 (**3g**) > 2.906 (Sunitinib) > 2.794 (**3f**) and is accepted. So, n-octanol/water distribution coefficients (logD7.4) at physiological pH=7.4 are as follows: 2.952 (**3g**) > > 2.630 (**3f**) > 2.542 (Sunitinib). Thus, compounds 3f and 3g were moderately soluble; some decrease in overall aqueous solubility could be improved by structural modification of the compounds. Thus, the physicochemical properties of compounds **3f** and **3g** demonstrated positive results.

## CONCLUSION

Twelve novel 5-fluoro-1,3-dihydro-2H-indol-2-one derivatives, thiazole-containing analogs of sunitinib, were designed and synthesized, and their antitumor activity was evaluated against the NCI-60 cancer cell lines. Target 2-[(5-fluoro-2-oxo-1,2-dihydro-3H-indol-3-ylidene)methyl] -1,3-thiazole-5-carboxamides 3a-k have been obtained according to a three-stage scheme: the carboxylation reaction of 2-(1,3-dioxolan-2-yl)thiazole, Knoevenagel condensation of 1,3-thiazole-5-carboxylic acid 1 with 5-fluoro-1,3-dihydro-2H-indol-2-one, and subsequent acylation of primary and secondary amines with 2-[(E)-(5-fluoro-2-oxo-1,2-dihydro-3H-indol-3-ylidene)methyl]-1,3-thiazole-5-carboxylic acid 2. The NCI-60 cell lines were used in the one-dose assays to evaluate the anticancer activity of these compounds. The one-dose assay results confirmed that compound 3g exerted inhibitory activity (GI >70%) in 8 out of 9 types of cancer, except prostate cancer. The compound **3g** demonstrated cell growth inhibition against breast cancer (cell line T-47D, GI=96.17%; MCF7, GI=88.19%), lung cancer (cell line HOP-92, GI=95.95%), ovarian cancer (cell line NCI/ADR-RES, GI=95.13%; OVCAR-3, GI=87.02%), CNS cancer (SNB-75, GI=89.91%), and leukemia cells (SR, GI=84.49%; MOLT-4, GI=84.49%; HL-60, GI=80.21%). The compound **3g** showed cell growth inhibition against leukemia, CNS, and breast cancer at 6 - 31%, which was higher than sunitinib. The SAR analysis of synthesized compounds indicated that replacing the pyrrole group with a similar thiazole fragment in sunitinib led to high-active compounds **3f** and **3g**, which contained pyrrolidine and piperidine carbonyl groups in the 5-position of thiazole. Modifying this group on dimethylamine and diethylamine afforded low-activity compounds **3a - 3d**. The molecular docking analysis demonstrated the potential antitumor action mechanism of compound **3g** as a VEGFR2 inhibitor. Compared to sunitinib, the predicted molecular physicochemical properties of compounds **3f** and **3g** demonstrated a positive prognosis. Thus, the study results validated compounds **3f** and **3g** as receptor tyrosine kinase inhibitors.

## AUTHORS’ CONTRIBUTIONS

I.S., V.B., O.K. provided the conception and design of the study. I.S., V.Z., L.P. carried out data analysis and interpretation of the results. V.B. contributed to the conceptualization. I.S., V.S., O.L. performed an investigation. I.S. took part in writing the original draft. All authors reviewed the results and approved the final version of the manuscript.

## Figures and Tables

**Fig. (3):**
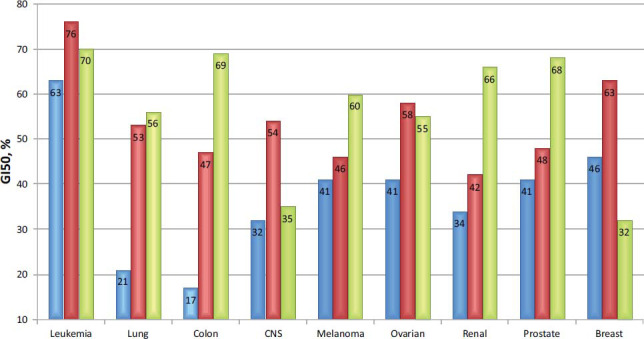
Mean GI values for NCI-60 subpanels for compounds **3f** (blue), **3g** (red), and sunitinib (green).

**Fig. (5):**
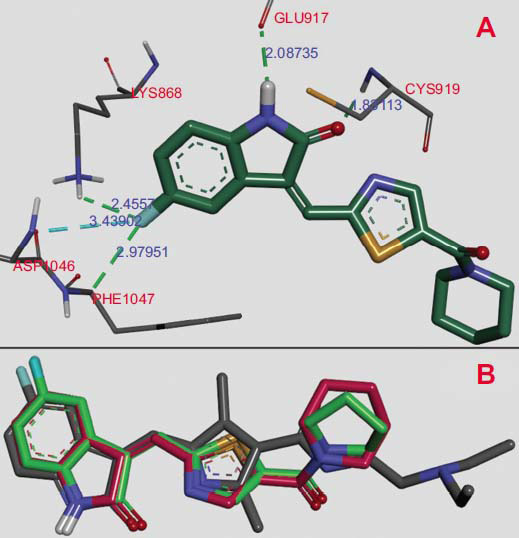
Molecular docking of the compound **3g** in the VEGFR2 ATP-binding pocket; (**A**). **3g** docking results; (**B**). Sunitinib (grey) and docking positions of **3f** (green) and **3g** (red).

**Table 1:** Mean values of growth inhibition for NCI-60 total panel and subpanels of tested compounds (GI, %).

**Panel/Subpanel**	**Compounds**											
	**2**	**3a**	**3b**	**3c**	**3d**	**3e**	**3f**	**3g**	**3h**	**3i**	**3j**	**3k**
Total	103.67 ±2.14	92.54 ±1,36	85.34 ±1,49	83.20 ±2,15	97.17 ±1,62	69.20 ±4.33	61,45 ±2,71	46,44 ±3,16	79,56 ±2,58	89,23 ±1,94	83.67 ±1,62	87,68 ±2,48
Leukemia	110.74 ±7.76	95,53 ±7,49	79,08 ±7,41	82,54 ±10,55	93,46 ±5,28	45.30 ±9.77	36,91 ±5,41	23,74 ±3,22	66,49 ±6,56	81,65 ±5,87	75,93 ±5,83	78,94 ±4,49
NSCLC	106.14 ±3.94	92,64 ±2,37	83,37 ±2,20	81,38 ±2,00	93,07 ±2,62	73.71 ±6.21	68,92 ±4,35	46,64 ±7,93	77,18 ±3,43	87,40 ±4,12	82,58 ±2,19	84,82 ±5,02
Colon Cancer	106.42 ±2.99	93,10 ±1,90	83,69 ±3,02	88,27 ±6,55	100,65 ±2,91	83.95 ±7.77	73,29 ±8,08	53,04 ±8,36	84,10 ±8,05	91,54 ±5,10	88,48 ±3,79	85,39 ±4,37
CNS Cancer	96.58 ±3.43	92,43 ±3,24	89,56 ±2,35	84,53 ±3,51	95,82 ±3,24	76.13 ±14.14	67,69 ±4,14	45,66 ±9,47	83,46 ±4,55	92,75 ±5,27	85,17 ±2,76	90,79 ±5,23
Melanoma	109.80 ±5.24	91,56 ±3,07	89,36 ±5,15	89,78 ±8,30	104,82 ±3,71	73.82 ±8.68	59,72 ±7,64	53,93 ±7,65	91,97 ±10,35	97,79 ±4,11	87,48 ±5,44	96,29 ±5,89
Ovarian Cancer	99.31 ±3.06	97,96 ±4,66	90,52 ±4,56	87,15 ±4,36	98,90 ±3,39	69.32 ±6.96	59,29 ±9,22	42,29 ±10,24	78,02 ±6,78	90,00 ±5,67	88,12 ±4,75	92,42 ±5,45
Renal Cancer	97.72 ±3.06	90,21 ±4,10	92,12 ±3,71	81,32 ±5,93	99,41 ±8,11	67.11 ±5.08	66,56 ±8,77	58,52 ±9,09	79,88 ±6,11	86,21 ±7,26	82,52 ±5,27	89,55 ±13,03
Prostate Cancer	109.77 ±0.23	85,13 ±1,18	74,36 ±0,31	72,79 ±4,00	88,91 ±7,06	79.09 ±3.34	59,36 ±6,75	52,51 ±2,09	74,74 ±4,80	88,37 ±5,33	77,07 ±1,18	83,03 ±5,09
Breast Cancer	96.58 ±7.47	89,42 ±7,24	79,84 ±5,53	73,71 ±10,12	90,53 ±4,84	54.39 ±16.13	53,77 ±9,08	37,35 ±14,04	71,34 ±10,26	83,92 ±8,51	77,01 ±8,01	80,90 ±9,38

**Table 2:** The molecular docking energies of the ligands 3f and 3g into the ATP-binding sites of RTKs and redocking results.

**Compounds**	**∆G, kcal/mol**			
	**VEGFR1**	**VEGFR2**	**PDGFRA**	**c-KIT**
**3f**	– 8.9	**– 9.9**	– 7.8	– 8.7
**3g**	– 8.9	**– 10.1**	– 8.3	– 8.5
Sunitinib	–	– 9.0	– 7.3	– 10.1
N-(4-Chlorophenyl)-2-((pyridin-4-ylmethyl)amino) benzamide	– 10.7	–	–	–

**Table 3:** The predicted physicochemical properties of the compounds 3f and 3g in comparison to Sunitinib.

**Physicochemical Properties**	**Compounds**		
	**3f**	**3g**	**Sunitinib**
Molecular Weight, MW	343.080	357.090	398.210
Number of rigid bonds, nRig	23	24	18
Formal charge, fChar	0	0	0
Number of heteroatoms, nHet	7	7	7
Number of atoms in the biggest ring, MaxRing	9	9	9
Number of rings, nRing	4	4	3
Number of rotatable bonds, nRot	3	3	8
Topological Polar Surface Area, TPSA, A^2^	62.300	62.300	77.230
Number of hydrogen bond donors, nHD	1	1	3
Number of hydrogen bond acceptors, nHA	5	5	6
LogD7.4, logP at physiological pH=7.4	2.630	2.952	2.542
LogS, Log of the aqueous solubility	-5.094	-5.480	-3.893
LogP, Log of the octanol/water partition coefficient	2.794	3.244	2.906

## Data Availability

All data generated or analysed during this study are included in this published article.

## LIST OF ABBREVIATIONS

VEGFR: Vascular Endothelial Growth Factor Receptor
PDGFRA: Platelet-derived Growth Factor Receptor Alpha
C-KIT: Receptor Tyrosine Kinase Kit
RTK: Receptor Tyrosine Kinase
RMSD: Root Mean Square Deviation
ADT: Autodock Tools
