# Supplementary material for: Design, Synthesis, and Antitumor Potential of New Thiazole-contained 5-Fluoro-2-Oxindole Derivatives as Sunitinib Analogues
Source: Curr Med Chem. 2024 Nov 4;32(25):5279–91. doi: 10.2174/0109298673346427241016100726 (PMC12613094; doi:10.2174/0109298673346427241016100726)
Supplement: Supplementary file 1 [file CMC-32-25-5279_SD1.pdf]

Supplementary Material

Design, Synthesis, and Antitumor Potential of New Thiazole-contained 5-Fluoro-2-Oxindole Derivatives as Sunitinib Analogues

Ivan Semenyuta[1,*], Oleksandr Los[2], Vitalii Sinenko[2], Victor Zhirnov[2], Lyudmyla Potikha[2,4], Oleksandr Kobzar[3] and Volodymyr Brovarets[2]

[1]Department of chemistry of natural compounds, V. P. Kukhar Institute of Bioorganic Chemistry and Petrochemistry, Kyiv, 02094, Ukraine; [2]Department of chemistry of bioactive nitrogen-containing heterocyclibases, V. P. Kukhar Institute of Bioorganic Chemistry and Petrochemistry, Kyiv, 02094, Ukraine; [3]Department of Mechanisms of Bioorganic reactions, V. P. Kukhar Institute of Bioorganic Chemistry and Petrochemistry, Kyiv, 02094, Ukraine; [4]Department of Chemistry, Taras Shevchenko National University, Kyiv, 01601, Ukraine

Table S1. Chemical structures of synthesized compounds 2 and 3a-3k.

Number	NSC number	Structure/UIPAC name
**2**	852265	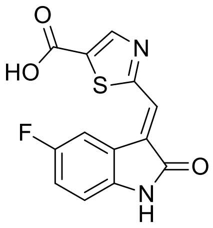 2-[(E)-(5-fluoro-2-oxo-1,2-dihydro-3H-indol-3-ylidene)methyl]-1,3-thiazole-5-carboxylic acid
**3a**	846122	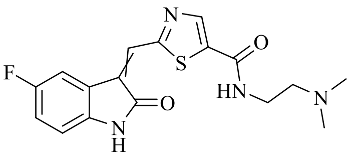 N-[2-(dimethylamino)ethyl]-2-[(5-fluoro-2-oxo-1,2-dihydro-3H-indol-3-ylidene)methyl]-1,3-thiazole-5-carboxamide
**3b**	846124	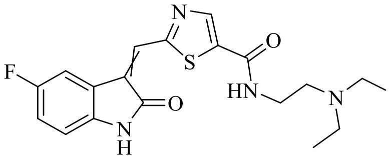 N-[2-(diethylamino)ethyl]-2-[(5-fluoro-2-oxo-1,2-dihydro-3H-indol-3-ylidene)methyl]-1,3-thiazole-5-carboxamide
**3c**	846123	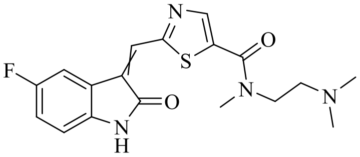 N-[2-(dimethylamino)ethyl]-2-[(5-fluoro-2-oxo-1,2-dihydro-3H-indol-3-ylidene)methyl]-N-methyl-1,3-thiazole-5-carboxamide

**3d**	848586	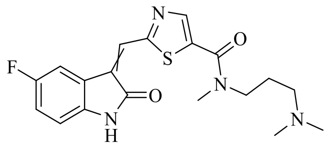 N-[3-(dimethylamino)propyl]-2-[(5-fluoro-2-oxo-1,2-dihydro-3H-indol-3-ylidene)methyl]-N-methyl-1,3-thiazole-5-carboxamide
**3e**	852264	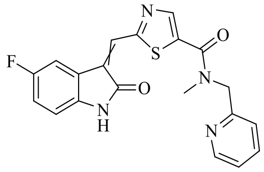 2-[(5-fluoro-2-oxo-1,2-dihydro-3H-indol-3-ylidene)methyl]-N-methyl-N-(pyridin-2-ylmethyl)-1,3-thiazole-5-carboxamide
**3f**	848587	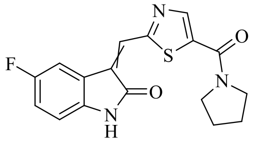 5-fluoro-3-{[5-(pyrrolidin-1-ylcarbonyl)-1,3-thiazol-2-yl]methylidene}-1,3-dihydro-2H-indol-2-one
**3g**	848588	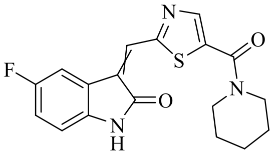 5-fluoro-3-{[5-(piperidin-1-ylcarbonyl)-1,3-thiazol-2-yl]methylidene}-1,3-dihydro-2H-indol-2-one
**3h**	848589	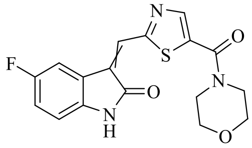 5-fluoro-3-{[5-(morpholin-4-ylcarbonyl)-1,3-thiazol-2-yl]methylidene}-1,3-dihydro-2H-indol-2-one
**3i**	846125	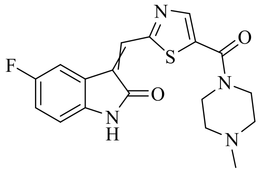 5-fluoro-3-({5-[(4-methylpiperazin-1-yl)carbonyl]-1,3-thiazol-2-yl}methylidene)-1,3-dihydro-2H-indol-2-one
**3j**	848591	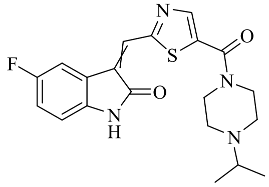 5-fluoro-3-[(5-{[4-(1-methylethyl)piperazin-1-yl]carbonyl}-1,3-thiazol-2-yl)methylidene]-1,3-dihydro-2H-indol-2-one
**3k**	848590	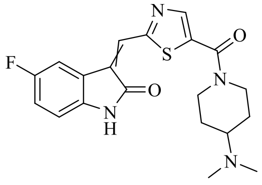 3-[(5-{[4-(dimethylamino)piperidin-1-yl]carbonyl}-1,3-thiazol-2-yl)methylidene]-5-fluoro-1,3-dihydro-2H-indol-2-one

1. EXPERIMENTAL SECTION

1.1. Reagents and Instruments

All reagents and solvents were purchased from Enamine Ltd. (www.enamine.net). TLC characterization was performed with pre-coated silica gel GF254 (0.2 mm). NMR spectra of the obtained products were recorded on a Varian Mercury 300 spectrometer (300 MHz for ^1H and 76 MHz for ^{13}C), Varian Unity Plus 400 spectrometer (400 MHz for ^1H and 100 MHz for ^{13}C), Bruker 170 Avance 500 spectrometer (500 MHz for ^1H and 126 MHz for ^{13}C), and Agilent ProPulse 600 spectrometer (600 MHz for ^1H and 151 MHz for ^{13}); ^1H NMR chemical shifts were calibrated using residual undeuterated DMSO (δ = 2.50 ppm) signal. ^{13}C NMR chemical shifts for ^{13}C NMR are reported relative to the central DMSO (δ = 40.45 ppm) signal. LC/MS spectra were recorded on an Agilent 1100 Series high-performance liquid chromatograph HPLC system equipped with a diode matrix with an Agilent LC/MS mass selective detector (chemical ionization). IR spectra of the compounds were recorded on a Bruker Vertex 70 instrument (*ATR* technique for oils and liquids; from KBr pellets for solids), and vibration frequencies were given in cm^{-1}. UV spectra of the compounds were recorded on a Cary 3500 multicell UV-Vis Spectrophotometer. Melting points were measured on a MPA100 OptiMelt automated melting point system. Elemental analyses were performed at the Analytical Laboratory of the V.P. Kukhar Institute of Bioorganic Chemistry and Petrochemistry of NAS of Ukraine.

1.2. Procedure for the Preparation of Compound 1

To a solution of 2-(1,3-dioxolan-2-yl)thiazole (50 g, 0.3181 mol) in THF (510 mL) was slowly added dropwise n-BuLi (146 mL, 2.5 M in hexanes) at -80 °C. The mixture was stirred at -80 - -70 °C for 1 h, and then cooled to -90 °C and CO_2 was bubbled through the reaction mixture maintaining the temperature below -70 °C. The solution was then warmed to rt, treated with water (400 mL), 3M HCl solution (146 mL) and brine (300 mL). The mixture was then extracted with MTBE (3×300 mL). Combined organic phases were washed with brine, the combined organic layer dried with anhydrous Na_2SO_4, and concentrated. 300 mL MTBE-Hexane (2 : 3) mixture was added to the residue and then a white solid was filtered, giving **1** as a colorless crystals.

1.3. Procedure for the Preparation of Compound 2

5-Fluoroindolin-2-one (7.51 g, 49.7 mmol) and PTSA (0.60 g, 3.5 mmol) were added to a solution of 2-(1,3-dioxolan-2-yl)thiazole-5-carboxylic acid **1** (10.00 g, 49.7 mmol) in acetic acid (150 mL). The resulting solution was boiled for 4 hours, a red precipitate fell out during the reaction. The precipitate was filtered the next day and washed with acetic acid, giving **2**.

1.4. General Procedure for the Preparation of Compounds 3a-k

1-Ethyl-3-(3-dimethylaminopropyl)carbodiimide hydrochloride (1.98 g, 10.34 mmol), hydroxybenzotriazole (1.40 g, 10.34 mmol), and triethylamine (1.40 g, 13.79 mmol) were added to the suspension of 2-[(*E*)-(5-fluoro-2-oxo-1,2-dihydro-3H-indol-3-ylidene)methyl]-1,3-thiazole-5-carboxylic acid **2** (2.00 g, 6.89 mmol) in THF (100 mL). The resulting suspension was stirred for 30 min at room temperature. Then the corresponding amine (13.79 mmol) was added and stirred for 3 days, treated with a saturated solution of $NaHCO_3$ (50 ml) and stirred for 10 min. The reaction mixture was extracted with THF/Ethyl acetate 1:1 (3×50 mL). The combined organic layer was washed with brine solution (20 mL), dried over anhydrous Na_2SO_4, filtered and concentrated under reduced pressure to obtain the crude product. The product was purified by column chromatography or recrystallization.

2. IDENTIFICATION DATA OF COMPOUNDS

2.1. 2-(1,3-Dioxolan-2-yl)-1,3-thiazole-5-carboxylic acid (1)

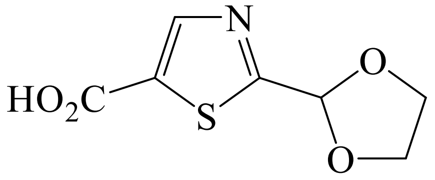

White solid; yield 57.60 g, 90%. M.p. 154-155 °C. ^1H NMR (400 MHz, DMSO-d_6) δ 8.33 (s, 1H, H4), 6.07 (s, 1H, CH), 4.09–3.99 (m, 4H, $(CH_2)_2$). ^{13}C NMR (100.6 MHz, DMSO-d_6) δ 175.7 (C2), 164.2 (CO_2H), 150.2 (C4), 133.8 (C5), 101.1 (CH), 67.5 (2C, CH_2). LC/MS (I, %) 202.0 (100) $[M+H]^+$. Anal. calcd for $C_7H_7NO_4S$ C (41.79), H (3.51), N (6.96), S (15.94), found C C (41.67), H (3.53), N (6.98), S (15.91).

2.2 2-[(E)-(5-fluoro-2-oxo-1,2-dihydro-3H-indol-3-ylidene)methyl]-1,3-thiazole-5-carboxylic acid (2)

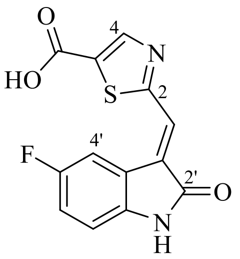

Red solid; yield 12.26 g, 85%. M.p. >250 °C. ^{1}H NMR (600 MHz, DMSO-d$_6$) δ 10.72 (s, 1H, NH), 8.89 (dd, J_{HF} 9.0 Hz, $^4J_{HH}$ 2.0 Hz, 1H, H4’), 8.70 (s, 1H, H4), 7.68 (s, 1H, CH), 7.18 (td, *J* 7.5 Hz, $^4J_{HH}$ 2.0 Hz, 1H, H6’), 6.86 (dd, $^3J_{HH}$ 7.0 Hz, J_{HF} 4.5 Hz, 1H, H7’). ^{13}C NMR (150.8 MHz, DMSO-d$_6$) δ 169.1 (C2’), 166.8 (C5-CO), 162.2 (C2), 157.8 (d, *J* 233.8 Hz, C5’), 150.2 (C4), 140.9 (d, *J* 1.5 Hz, C7a’), 134.8 (C5), 130.4 (d, *J* 3.0 Hz, C3’), 124.4 (CH), 122.1 (d, *J* 9.0 Hz, C3a’), 118.8 (d, *J* 21.1 Hz, C6’), 114.6 (d, *J* 30.2 Hz, C4’), 111.0 (d, *J* 7.5 Hz, C7’). UV/Vis (DMSO, nm (mol^{-1}dm^{3}cm^{-1})) λ_{max} (ε) 440 (4080), 376 (19400), 357 (22800), 269 (10500). LC/MS (I, %) 291.0 (100) [*M*+H]$^+$. Anal. calcd for $C_{13}H_7FN_2O_3S$ C (53.79), H (2.43), N (9.65), S (11.05), found C (53.85), H (2.39), N (9.68), S (11.00).

2.3. N-[2-(dimethylamino)ethyl]-2-[(5-fluoro-2-oxo-1,2-dihydro-3H-indol-3-ylidene)methyl]-1,3-thiazole-5-carboxamide (3a)

Synthesized following the general procedure using *N*,*N*-dimethylethane-1,2-diamine. The product was purified by column chromatography on silica gel using THF as eluent, and then THF/MeOH(5%)/Et$_3$N(2%) to afford the mixture of isomerz (*E*- : *Z*- = 74 : 26) of desired compound **3a**.

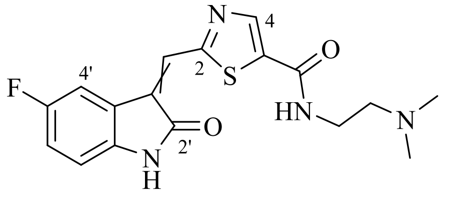

Red solid; yield 0.77 g, 31%. M.p. 224-226 °C. ^{1}H NMR (400 MHz, DMSO-d$_6$) δ 10.93 (br s, NH *Z*-), 10.74 (br s, NH *E*-), 8.90 (dd, J_{HF} 10.0 Hz, $^4J_{HH}$ 2.4 Hz, H4’ *E*-), 8.80 (t, *J* 5.2 Hz, NH *E*-), 8.74 (t, *J* 5.6 Hz, NH *Z*-), 8.71 (s, H4 *E*-), 8.57 (s, H4 *Z*-), 8.13 (s, CH *Z*-), 7.84 (dd, J_{HF} 8.0 Hz, $^4J_{HH}$ 2.2 Hz, H4’ *Z*-), 7.66 (s, CH *E*-), 7.18 (td, *J* 9.6 Hz, $^4J_{HH}$ 2.4 Hz, H6’ *E*-), 7.18 (td, *J* 10.0 Hz, $^4J_{HH}$ 2.2 Hz, H6’ *Z*-), 6.86 (dd, $^3J_{HH}$ 8.4 Hz, J_{HF} 4.4 Hz, 1H, H7’), 3.39–3.35 (m, 2H, CH$_2$), 2.43 (t, *J* 6.8 Hz, 2H, CH$_2$), 2.19 (s, 6H, N(CH$_3$)$_2$). ^{13}C NMR (100.6 MHz, DMSO-d$_6$) δ 169.7 (C2’ *E*-), 168.1 (C2’ *Z*-), 165.4 (C2 *E*-), 162.8 (C2 *Z*-), 160.9 (C5-CO *Z*-), 159.5 (C5-CO, *E*-), 159.0 (d, *J* 225.6 Hz, C5’ *Z*-), 158.4 (d, *J* 233.7 Hz, C5’ *E*-), 146.2 (C4 *E*-), 144.8 (C4 *Z*-), 141.2 (d, *J* 1.9 Hz, C7a’ *E*-), 140.5 (C5 *E*-), 140.2 (C5 *Z*-), 138.9 (d, *J* 1.6 Hz, C7a’ *Z*-), 130.2 (d, *J* 3.6 Hz, C3’ *E*-), 129.7 (CH Z-), 129.5 (d, *J* 3.0 Hz, C3’ *E*-), 125.4 (d, *J* 8.3 Hz, C3a’ *Z*-), 125.1 (CH *E*-), 122.8 (d, *J* 10.0 Hz, C3a’ *E*-), 119.1 (d, *J* 24.2 Hz, C6’ *E*-), 118.0 (d, *J* 24.0 Hz, C6’ *Z*-), 115.0 (d, *J* 27.4 Hz, C4’ *E*-), 111.8 (d, *J* 8.8 Hz, C7’ *Z*-), 111.5 (d, *J* 9.0 Hz, C7’ *E*-), 109.7 (d, *J* 25.4 Hz, C4’ *Z*-), 59.0 (CH$_2$), 46.1 (2C, N(CH$_3$)$_2$), 38.4 (CH$_2$). UV/Vis (DMSO, nm (mol^{-1}dm^{3}cm^{-1})) λ_{max} (ε) 437 (3810), 378 (17600), 360 (20800), 264 (10600). LC/MS (I, %) 361.1 (100) [*M*+H]$^+$. Anal. Calcd. for $C_{17}H_{17}FN_4O_2S$ C (56.65), H (4.75), N (15.55), S (8.90), found C (56.70), H (4.78), N (15.57), S (8.87).

2.4. N-[2-(diethylamino)ethyl]-2-[(5-fluoro-2-oxo-1,2-dihydro-3H-indol-3-ylidene)methyl]-1,3-thiazole-5-carboxamide (3b)

Synthesized following the general procedure using *N*,*N*-diethylethane-1,2-diamine. The product was purified by column chromatography on silica gel using THF as eluent, and then THF/Et$_3$N(2%) to afford the mixture of isomerz (*E*- : *Z*- = 77 : 23) of desired compound **3b**.

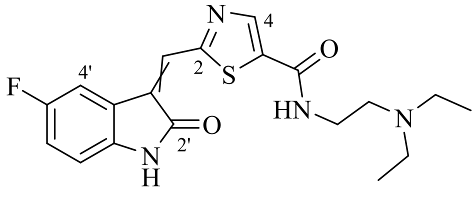

Red solid; yield 0.80 g, 30%. M.p. 185-187 °C. ^{1}H NMR (500 MHz, DMSO-d_6) δ 10.92 (br s, NH *Z*-), 10.72 (br s, NH *E*-), 8.88 (dd, J_{HF} 9.5 Hz, $^4J_{HH}$ 2.0 Hz, H4' *E*-), 8.76 (br t, *J* 6.0 Hz, NH *E*-), 8.72–8.68 (m, NH *Z*-), 8.67 (s, H4 *E*-), 8.53 (s, H4 *Z*-), 8.11 (s, CH *Z*-), 7.81 (br d, J_{HF} 8.5 Hz, H4' *Z*-), 7.64 (s, CH *E*-), 7.16 (td, *J* 8.5 Hz, $^4J_{HH}$ 2.0 Hz, H6' *E*-), 7.11 (br t, J_{HF} 9.0 Hz, H6' *Z*-), 6.86–6.83 (m, 1H, H7'), 3.344–3.28 (m, 2H, CH_2), 2.55 (t, *J* 7.5 Hz, 2H, CH_2), 2.51–2.47 (m, 4H, $N(CH_2CH_3)_2$), 2.19 (t, *J* 8.5 Hz, 6H, $N(CH_2CH_3)_2$). ^{13}C NMR (125.6 MHz, DMSO-d_6) δ 170.1 (C2' *E*-), 168.5 (C2' *Z*-), 165.8 (C2 *E*-), 163.2 (C2 *Z*-), 161.3 (C5-CO *Z*-), 160.6 (C5-CO, *E*-), 159.5 (d, *J* 239.5 Hz, C5' *Z*-), 158.8 (d, *J* 234.1 Hz, C5' *E*-), 146.6 (C4 *E*-), 145.1 (C4 *Z*-), 141.7 (C7a' *E*-), 141.0 (C5 *E*-), 140.6 (C5 *Z*-), 139.4 (C7a' *Z*-), 130.7 (d, *J* 2.4 Hz, C3' *E*-), 130.1 (CH Z-), 130.0 (d, *J* 3.8 Hz, C3' *E*-), 125.8 (d, *J* 8.7 Hz, C3a' *Z*-), 125.4 (CH *E*-), 123.2 (d, *J* 9.9 Hz, C3a' *E*-), 119.5 (d, *J* 23.6 Hz, C6' *E*-), 118.4 (d, *J* 23.9 Hz, C6' *Z*-), 115.4 (d, *J* 27.4 Hz, C4' *E*-), 112.2 (d, *J* 8.3 Hz, C7' *Z*-), 111.9 (d, *J* 7.5 Hz, C7' *E*-), 110.0 (d, *J* 25.8 Hz, C4' *Z*-), 52.8 (CH_2), 48.2 (2C, $N(CH_2CH_3)_2$), 39.2 (CH_2), 13.3 (2C, $N(CH_2CH_3)_2$). UV/Vis (DMSO, nm (mol^{-1}dm^{3}cm^{-1})) λ_{max} (ε) 430 (3710), 362 (19700), 269 (11800). LC/MS (I, %) 389.1 (100) [*M*+H]$^+$. Anal. Calcd. for $C_{19}H_{21}FN_4O_2S$ C (58.75), H (5.45), N (14.42), S (8.25), found C (58.81), H (5.42), N (14.44), S (8.26).

2.5. N-[2-(dimethylamino)ethyl]-2-[(5-fluoro-2-oxo-1,2-dihydro-3H-indol-3-ylidene)methyl]-N-methyl-1,3-thiazole-5-carboxamide (3c)

Synthesized following the general procedure using *N*,*N*,*N*'-trimethylethane-1,2-diamine to afford the mixture of isomerz (*E*- : *Z*- = 73 : 27) of desired compound **3c** without addition purification.

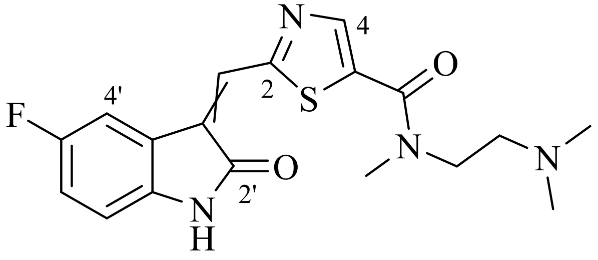

Red solid; yield 1.14 g, 44%. M.p. 107-108 °C. ^{1}H NMR (301.55 MHz, DMSO-d_6) δ 10.94 (br s, NH *Z*-), 10.76 (br s, NH *E*-), 8.94 (dd, J_{HF} 9.6 Hz, $^4J_{HH}$ 2.1 Hz, H4' *E*-), 8.56 (br s, H4 *E*-), 8.36 (br s, H4 *Z*-), 8.15 (s, CH *Z*-), 7.84 (dd, J_{HF} 8.7 Hz, $^4J_{HH}$ 2.4 Hz, H4' *Z*-), 7.66 (s, CH *E*-), 7.21–7.09 (m, 1H, H6'), 6.86 (dd, *J* 8.4 Hz, J_{HF} 4.5 Hz, 1H, H7'), 3.57 (t, *J* 6.3 Hz, 3H, NCH_3), 3.10–2.96 (m, 2H, CH_2), 2.46 (t, *J* 7.0 Hz, 2H, CH_2), 2.17 (br s, 6H, $N(CH_3)_2$). ^{13}C NMR (75.83 MHz, DMSO-d_6) δ 169.7 (C2' *E*-), 168.1 (C2' *Z*-), 164.6 (C2 *E*-), 162.4 (C2 *Z*-), 160.2 (C5-CO), 159.0 (d, *J* 223.5 Hz, C5' *Z*-), 158.4 (d, *J* 233.3 Hz, C5' *E*-), 146.9 (C4 *E*-), 145.0 (C4 *Z*-), 141.2 (C7a'), 138.9 (C5), 130.1 (d, *J* 2.6 Hz, C3'), 129.4 (CH *Z*-), 125.4 (d, *J* 9.2 Hz, C3a' *Z*-), 124.7 (CH *E*-), 122.8 (d, *J* 9.6 Hz, C3a' *E*-), 119.0 (d, *J* 24.3 Hz, C6' *E*-), 117.9 (d, *J* 25.3 Hz, C6' *Z*-), 114.1 (d, *J* 28.4 Hz, C4' *E*-), 111.8 (d, *J* 8.1 Hz, C7' *Z*-), 111.5 (d, *J* 8.4 Hz, C7' *E*-), 109.7 (d, *J* 25.1 Hz, C4' *Z*-), 57.1 (CH_2), 47.0 (CH_2), 46.2 (NCH_3), 38.4 (NCH_3). UV/Vis (DMSO, nm (mol^{-1}dm^{3}cm^{-1})) λ_{max} (ε) 429 (3410), 361 (17900), 265 (11400). LC/MS (I, %) 375.2 (97) [*M*+H]$^+$. Anal. calcd. for $C_{18}H_{19}FN_4O_2S$ C (57.74), H (5.11), N (14.96), S (8.56), found C (57.79), H (5.09), N (14.94), S (8.59).

2.6. N-[3-(Dimethylamino)propyl]-2-[(5-fluoro-2-oxo-1,2-dihydro-3H-indol-3-ylidene)methyl]-N-methyl-1,3-thiazole-5-carboxamide (3d)

Synthesized following the general procedure using *N*,*N*,*N*'-trimethylpropil-1,3-diamine. The product was purified by column chromatography on silica gel using THF as eluent, and then THF/Et_3N(2%) to afford the mixture of isomerz (*E*- : *Z*- = 71 : 29) of desired compound **3d**.

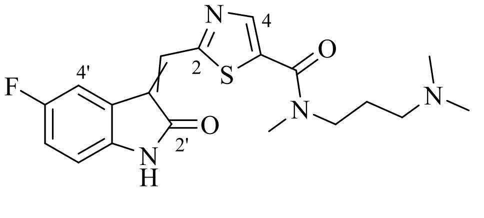

Red solid; yield 0.91 g, 34%. M.p. 89-90°C. ^{1}H NMR (400 MHz, DMSO-d$_6$) δ 10.97 (br s, NH *Z*-), 10.79 (br s, NH *E*-), 8.96 (br d, J_{HF} 9.2 Hz, H4’ *E*-), 8.61 (br s, H4 *E*-), 8.54 (br s, H4 *Z*-), 8.17 (br s, CH *Z*-), 7.86 (br d, J_{HF} 8.4 Hz, H4’ *Z*-), 7.68 (br s, CH *E*-), 7.18 (br t, *J* 8.8 Hz, H6’ *E*-), 7.13 (br t, *J* 8.4 Hz, H6’ *Z*-), 6.87 (dd, $^{3}J_{HH}$ 8.0 Hz, J_{HF} 4.0 Hz, 1H, H7’), 3.51–3.44 (m, 3H, NCH$_3$), 3.09–2.96 (m, 2H, CH$_2$), 2.25–2.05 (m, 8H, N(CH$_3$)$_2$, CH$_2$), 1.69–1.67 (m, 2H, CH$_2$). ^{13}C NMR (150.8 MHz, DMSO-d$_6$) δ 169.7 (C2’ *E*-), 168.1 (C2’ *Z*-), 164.7 (br, C2 *E*-), 164.2 (br, C2 *Z*-), 161.9 (br, C5-CO *Z*-), 161.4 (br, C5-CO *E*-), 159.0 (d, *J* 235.9 Hz, C5’ *Z*-), 158.4 (d, *J* 233.4 Hz, C5’ *E*-), 146.9 (C4 *E*-), 146.1 (C4 *Z*-), 141.2 (C7a’ *E*-), 140.1 (C5 *E*-), 139.3 (C5 *Z*-), 138.4 (C7a’ *Z*-), 130.1 (C3’ *E*-), 129.4 (C3’ *Z*-), 128.9 (CH *Z*-), 125.3 (d, *J* 9.0 Hz, C3a’ *Z*-), 124.7 (CH *E*-), 122.8 (d, *J* 10.7 Hz, C3a’ *E*-), 118.9 (d, *J* 24.6 Hz, C6’ *E*-), 117.9 (d, *J* 24.4 Hz, C6’ *Z*-), 115.0 (d, *J* 27.6 Hz, C4’ *E*-), 111.7 (d, *J* 7.8 Hz, C7’ *Z*-), 111.4 (d, *J* 8.0 Hz, C7’ *E*-), 109.7 (d, *J* 24.6 Hz, C4’ *Z*-), 57.3 (br, CH$_2$), 47.6 (br, CH$_2$), 45.9 (NCH$_3$), 35.3 (NCH$_3$), 25.5 (br, CH$_2$). UV/Vis (DMSO, nm (mol^{-1}dm^{3}cm^{-1})) λ_{max} (ε) 435 (3050), 376 (14000), 359 (16200), 267 (9340). LC/MS (I, %) 389.2 (100) [*M*+H]$^+$, RT 0.978 min; 389.2 (100) [*M*+H]$^+$, RT 0.991 min. Anal. calcd. for $C_{19}H_{21}FN_4O_2S$ C (58.75), H (5.45), N (14.42), S (8.25), found C (58.80), H (5.47), N (14.39), S (8.28).

2.7. 2-[(5-Fluoro-2-oxo-1,2-dihydro-3H-indol-3-ylidene)methyl]-N-methyl-N-(pyridin-2-ylmethyl)-1,3-thiazole-5-carboxamide (3e)

Synthesized following the general procedure using *N*-methyl-1-(pyridin-2-yl)methanamine. The product was purified by recrystallization from EtOH/THF (1:1) to afford the mixture of isomerz (*E*- : *Z*- = 85 : 15) of desired compound **3e**.

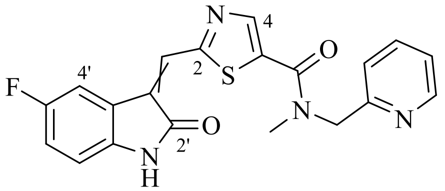

Red solid; yield 0.95 g, 35%. M.p. 208-210°C. ^{1}H NMR (500 MHz, DMSO-d$_6$) δ 10.90 (br s, NH *Z*-), 10.70 (br s, NH *E*-), 8.93–8.80 (m, H-Pyridinyl *Z*-, H4’ *E*-), 8.69–8.47 (m, H-Pyridinyl *E*-, H4), 8.13–8.07 (m, CH *Z*-), 7.80–7.78 (m, H-Pyridinyl *Z*-, CH *E*-), 7.65–7.60 (m, H-Pyridinyl *E*-), 7.35–7.30 (m, 2H, H-Pyridinyl), 7.15–7.05 (m, 1H, H6’), 6.85–6.80 (m, 1H, H7’), 4.85–4.78 (m, 2H, CH$_2$), 3.36–3.30 (m, 3H, NCH$_3$). ^{13}C NMR (125.6 MHz, DMSO-d$_6$) δ 169.6 (C2’ *E*-), 168.0 (C2’ *Z*-), 165.0 (br, C2 *E*-), 164.5 (br, C2 *Z*-), 162.7 (br, C5-CO *Z*-), 161.9 (br, C5-CO *E*-), 158.9 (d, *J* 234.8 Hz, C5’ *Z*-), 158.3 (d, *J* 234.6 Hz, C5’ *E*-), 157.4 (br, C-Pyridinyl), 156.8 (br, C-Pyridinyl), 150.5 (br, C-Pyridinyl), 150.1 (br, C-Pyridinyl), 147.3 (br, C4 *E*-), 146.2 (br, C4 *Z*-), 141.1 (C7a’ *E*-), 138.4 (C7a’ *Z*-), 138.4 (C5), 137.9 (br, C-Pyridinyl), 130.2 (br, C3’), 129.3 (CH *Z*-), 125.3 (d, *J* 9.3 Hz, C3a’ *Z*-), 124.5 (CH *E*-), 123.7 (br, C-Pyridinyl), 123.3 (br, C-Pyridinyl), 123.0 (br, C-Pyridinyl), 122.7 (d, *J* 8.9 Hz, C3a’ *E*-), 122.5 (br, C-Pyridinyl), 118.9 (d, *J* 23.0 Hz, C6’ *E*-), 117.9 (d, *J* 23.5 Hz, C6’ *Z*-), 115.0 (d, *J* 27.0 Hz, C4’ *E*-), 111.7 (d, *J* 8.4 Hz, C7’ *Z*-), 111.4 (d, *J* 8.2 Hz, C7’ *E*-), 109.6 (d, *J* 24.8 Hz, C4’ *Z*-), 56.6 (br, CH$_2$), 54.3 (br, CH$_2$), 38.9 (br, NCH$_3$), 35.2 (br, NCH$_3$). LC/MS (I, %) 395.0 (100) [*M*+H]$^+$, RT 1.230 min; 395.0 (100) [*M*+H]$^+$, RT 1.197 min. Anal. calcd. for $C_{20}H_{15}FN_4O_2S$ C (60.90), H (3.83), N (14.20), S (8.13), found C (60.97), H (3.80), N (14.23), S (8.10).

2.8. 5-Fluoro-3-{[5-(pyrrolidin-1-ylcarbonyl)-1,3-thiazol-2-yl]methylidene}-1,3-dihydro-2H-indol-2-one (3f)

Synthesized following the general procedure using pyrrolidine. The product was purified by recrystallization from EtOH/THF (1:1) to afford the mixture of isomerz (*E*- : *Z*- = 84 : 16) of desired compound **3f**.

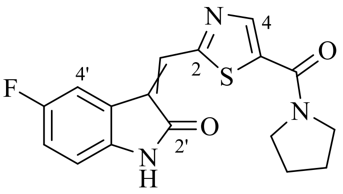

Red solid; yield 0.92 g, 39%. M.p. 235-237°C. ^{1}H NMR (301.55 MHz, DMSO-d_6) δ 10.89 (br s, NH *Z*-), 10.72 (br s, NH *E*-), 8.93 (d, J_{HF} 9.6 Hz, H4’ *E*-), 8.63 (s, H4 *E*-), 8.44 (br s, H-4 *Z*-), 8.10 (s, CH *Z*-), 7.82 (d, J_{HF} 8.7 Hz, H4’ *Z*-), 7.62 (s, CH *E*-), 7.19–7.11 (m, 1H, H-6’), 6.86 (d.d, $^{3}J_{HH}$ 8.4 Hz, J_{HF} 4.5 Hz, 1H, H7’), 3.80–3.73 (m, 2H, NCH$_2$), 3.51–3.47 (m, 2H, NCH$_2$), 2.00–1.83 (m, 4H, CH$_2$). ^{13}C NMR (75.83 MHz, DMSO-d_6) δ 169.6 (C2’ *E*-), 168.0 (C2’ *Z*-), 165.0 (C2 *E*-), 162.4 (C2 *Z*-), 160.2 (C5-CO *Z*-), 159.4 (C5-CO *E*-),158.3 (d, *J* 235.9 Hz, C5’ *Z*-), 158.4 (d, *J* 235.4 Hz, C5’ *E*-), 146.9 (C4 *E*-), 145.4 (C4 *Z*-), 141.2 (C7a’ *E*-), 140.5 (C5 *E*-), 140.1 (C5 *Z*-), 138.9 (C7a’ *Z*-), 130.2 (d, *J* 2.9 Hz, C3’ *E*-), 129.6 (d, *J* 2.2 Hz, C3’ *Z*-), 129.5 (CH Z-), 125.3 (d, *J* 9.1 Hz, C3a’ *Z*-), 124.7 (CH *E*-), 122.8 (d, *J* 11.1 Hz, C3a’ *E*-), 119.0 (d, *J* 24.0 Hz, C6’ *E*-), 117.9 (d, *J* 23.2 Hz, C6’ *Z*-), 115.1 (d, *J* 28.7 Hz, C4’ *E*-), 111.7 (d, *J* 7.7 Hz, C7’ *Z*-), 111.4 (d, *J* 8.0 Hz, C7’ *E*-), 109.6 (d, *J* 25.0 Hz, C4’ *Z*-), 49.2 (NCH$_2$), 48.1 (NCH$_2$ *Z*-), 48.0 (NCH$_2$ *E*-), 27.0 (CH$_2$), 24.5 (CH$_2$). UV/Vis (DMSO, nm (mol^{-1}dm^{3}cm^{-1})) λ_{max} (ε) 438 (4580), 377 (21200), 359 (24900), 264 (12000). LC/MS (I, %) 344.2 (100) [*M*+H]$^+$, RT 1.250 min; 344.2 (100) [*M*+H]$^+$, RT 1.298 min. Anal. calcd. for $C_{17}H_{14}FN_3O_2S$ C (59.46), H (4.11), N (12.24), S (9.34), found C (59.40), H (4.15), N (12.28), S (9.32).

2.9. 5-Fluoro-3-{[5-(piperidin-1-ylcarbonyl)-1,3-thiazol-2-yl]methylidene}-1,3-dihydro-2H-indol-2-one (3g)

Synthesized following the general procedure using piperidine. The product was purified by recrystallization from EtOH to afford the mixture of isomerz (*E*- : *Z*- = 87 : 13) of desired compound **3g**.

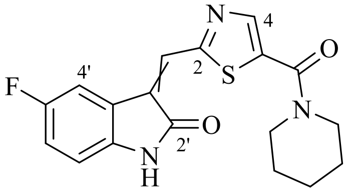

Red solid; yield 0.89 g, 36%. M.p. 207-209°C. ^{1}H NMR (301.55 MHz, DMSO-d_6) δ 10.90 (br s, NH *Z*-), 10.72 (br s, NH *E*-), 8.94 (dd, J_{HF} 10.3 Hz, $^{4}J_{HH}$ 2.4 Hz, H4’ *E*-), 8.47 (s, H4 *E*-), 8.26 (s, H4 *Z*-), 8.17 (s, CH *Z*-), 7.85 (dd, J_{HF} 8.4 Hz, $^{4}J_{HH}$ 1.5 Hz, H4’ *Z*-), 7.67 (s, CH *E*-), 7.21–7.12 (m, 1H, H6’), 6.86 (dd, $^{3}J_{HH}$ 8.4 Hz, J_{HF} 4.5 Hz, 1H, H7’), 3.65–3.55 (m, 4H, NCH$_2$), 1.65–1.50 (m, 6H, CH$_2$). ^{13}C NMR (75.83 MHz, DMSO-d_6) δ 169.7 (C2’ *E*-), 168.1 (C2’ *Z*-), 164.2 (C2 *E*-), 161.5 (C2 *Z*-), 161.1 (C5-CO *Z*-), 160.4 (C5-CO *E*-), 159.0 (d, *J* 235.5 Hz, C5’ *Z*-), 158.4 (d, *J* 234.6 Hz, C5’ *E*-), 146.2 (C4 *E*-), 144.4 (C4 *Z*-), 141.1 (C7a’ *E*-), 138.8 (C-7a’ *Z*-), 138.2 (C5), 129.9 (d, *J* 2.9 Hz, C3’ *E*-), 129.4 (CH *Z*-), 129.2 (d, *J* 1.7 Hz, C3’ *Z*-), 125.4 (d, *J* 9.3 Hz, C3a’ *Z*-), 124.7 (CH *E*-), 122.8 (d, *J* 11.0 Hz, C3a’ *E*-), 118.9 (d, *J* 24.0 Hz, C6’ *E*-), 117.9 (d, *J* 24.1 Hz, C6’ *Z*-), 115.0 (d, *J* 27.5 Hz, C4’ *E*-), 111.7 (d, *J* 7.9 Hz, C7’ *Z*-), 111.4 (d, *J* 8.1 Hz, C7’ *E*-), 109.7 (d, *J* 25.1 Hz, C4’ *Z*-), 49.0 (NCH$_2$), 44.2 (NCH$_2$), 26.7 (CH$_2$), 24.8 (2C, CH$_2$). UV/Vis (DMSO, nm (mol^{-1}dm^{3}cm^{-1})) λ_{max} (ε) 432 (4430), 376 (20700), 360 (23600), 265 (12500). LC/MS (I, %) 358.0 (100) [*M*+H]$^+$, RT 1.111 min; 358.0 (100) [*M*+H]$^+$, RT 1.159 min. Anal. calcd. for $C_{18}H_{16}FN_3O_2S$ C (60.49), H (4.51), N (11.76), S (8.97), found C (60.52), H (4.49), N (11.79), S (8.99).

2.10. 5-Fluoro-3-{[5-(morpholin-4-ylcarbonyl)-1,3-thiazol-2-yl]methylidene}-1,3-dihydro-2H-indol-2-one (3h)

Synthesized following the general procedure using morpholine. The product was purified by recrystallization from EtOH/THF (1:1) to afford the mixture of isomerz (*E*- : *Z*- = 82 : 18) of desired compound **3h**.

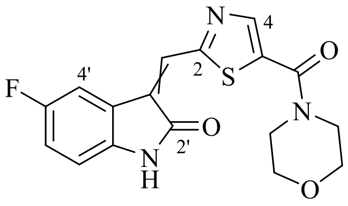

Red solid; yield 1.04 g, 42%. M.p. 221-223°C. ^{1}H NMR (301.55 MHz, DMSO-d$_6$) δ 10.92 (br s, NH *Z*-), 10.74 (br s, NH *E*-), 8.94 (dd, J_{HF} 10.0 Hz, $^4J_{HH}$ 2.7 Hz, H4' *E*-), 8.54 (s, H4 *E*-), 8.33 (s, H4 *Z*-), 8.17 (s, CH *Z*-), 7.85 (dd, J_{HF} 9.0 Hz, $^4J_{HH}$ 2.1 Hz, H4' *Z*-), 7.68 (s, CH *E*-), 7.21–7.09 (m, 1H, H-6'), 6.86 (dd, $^3J_{HH}$ 8.4 Hz, J_{HF} 4.5 Hz, 1H, H7'), 3.67–3.65 (m, 8H, CH$_2$). ^{13}C NMR (75.83 MHz, DMSO-d$_6$) δ 169.7 (C2' *E*-), 168.1 (C2' *Z*-), 164.6 (C2 *E*-), 161.9 (C2 *Z*-), 161.5 (C5-CO *Z*-), 160.9 (C5-CO *E*-),159.0 (d, *J* 236.2 Hz, C5' *Z*-), 158.4 (d, *J* 233.8 Hz, C5' *E*-), 146.7 (C4 *E*-), 145.0 (C4 *Z*-), 141.2 (C7a' *E*-), 138.9 (C7a' *Z*-), 137.6 (C5 *E*-), 137.5 (C5 *Z*-), 130.1 (d, *J* 4.2 Hz, C3' *E*-), 129.5 (d, *J* 2.7 Hz, C3' *Z*-), 129.3 (CH *Z*-), 125.3 (d, *J* 9.3 Hz, C3a' *Z*-), 124.7 (CH *E*-), 122.8 (d, *J* 9.5 Hz, C3a' *E*-), 119.0 (d, *J* 23.4 Hz, C6' *E*-), 118.0 (d, *J* 24.0 Hz, C6' *Z*-), 115.1 (d, *J* 26.6 Hz, C4' *E*-), 111.8 (d, *J* 8.0 Hz, C7' *Z*-), 111.5 (d, *J* 7.9 Hz, C7' *E*-), 109.8 (d, *J* 26.0 Hz, C4' *Z*-), 67.0 (2C, OCH$_2$), 48.4 (NCH$_2$), 44.0 (NCH$_2$). UV/Vis (DMSO, nm (mol^{-1}dm^{3}cm^{-1})) λ_{max} (ε) 435 (4070), 375 (19200), 358 (21900), 266 (11400). LC/MS (I, %) 360.0 (100) [*M*+H]$^+$, RT 0.965 min; 360.0 (100) [*M*+H]$^+$, RT 0.993 min. Anal. calcd. for $C_{17}H_{14}FN_3O_3S$ C (56.82), H (3.93), N (11.69), S (8.92), found C (56.87), H (3.96), N (11.65), S (8.90).

2.11. 5-Fluoro-3-({5-[(4-methylpiperazin-1-yl)carbonyl]-1,3-thiazol-2-yl}methylidene)-1,3-dihydro-2H-indol-2-one (3i)

Synthesized following the general procedure using 1-methylpiperazine. The product was purified by recrystallization from EtOH/THF (1:1) to afford the mixture of isomerz (*E*- : *Z*- = 80 : 20) of desired compound **3i**.

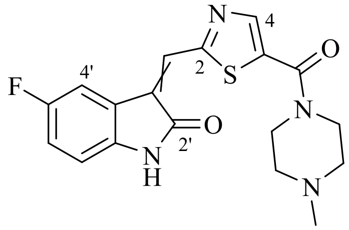

Red solid; yield 1.05 g, 41%. M.p. 153-155°C. ^{1}H NMR (301.55 MHz, DMSO-d$_6$) δ 10.92 (br s, NH *Z*-), 10.74 (br s, NH *E*-), 8.94 (br d, J_{HF} 9.6 Hz, H4' *E*-), 8.50 (s, H4 *E*-), 8.29 (s, H4 *Z*-), 8.16 (s, CH *Z*-), 7.85 (br d, J_{HF} 8.1 Hz, H4' *Z*-), 7.66 (s, CH *E*-), 7.20–7.09 (m, 1H, H6'), 6.87–6.83 (m, 1H, H7'), 3.75–3.50 (m, 4H, CH$_2$), 2.45–2.25 (m, 4H, CH$_2$), 2.20 (s, 3H, CH$_3$). ^{13}C NMR (75.83 MHz, DMSO-d$_6$) δ 169.7 (C2' *E*-), 168.1 (C2' *Z*-), 164.5 (C2 *E*-), 161.8 (C2 *Z*-), 161.3 (C5-CO *Z*-), 160.7 (C5-CO *E*-), 159.0 (d, *J* 236.7 Hz, C5' *Z*-), 158.4 (d, *J* 233.7 Hz, C5' *E*-), 146.6 (C4 *E*-), 144.8 (C4 *Z*-), 141.2 (C7a' *E*-), 138.9 (C7a' *Z*-), 137.8 (C5 *E*-), 137.7 (C5 *Z*-), 130.1 (d, *J* 2.7 Hz, C3' *E*-), 129.4 (d, *J* 2.5 Hz, C3' *Z*-), 129.3 (CH *Z*-), 125.4 (d, *J* 10.9 Hz, C3a' *Z*-), 124.6 (CH *E*-), 122.8 (d, *J* 8.7 Hz, C3a' *E*-), 119.0 (d, *J* 23.5 Hz, C6' *E*-), 117.9 (d, *J* 26.0 Hz, C6' *Z*-), 115.0 (d, *J* 27.5 Hz, C4' *E*-), 111.8 (d, *J* 8.4 Hz, C7' *Z*-), 111.5 (d, *J* 7.1 Hz, C7' *E*-), 109.7 (d, *J* 27.7 Hz, C4' *Z*-), 55.35 (2C, NCH$_2$), 48.0 (br, NCH$_2$), 46.4 (CH$_3$), 43.3 (br, NCH$_2$). UV/Vis (DMSO, nm (mol^{-1}dm^{3}cm^{-1})) λ_{max} (ε) 435 (3730), 375 (17400), 359 (20000), 264 (11500). LC/MS (I, %) 373.0 (100) [*M*+H]$^+$, RT 0.687 min; 373.0 (100) [*M*+H]$^+$, RT 0.720 min. Anal. calcd. for $C_{18}H_{17}FN_4O_2S$ C (58.05), H (4.60), N (15.04), S (8.61), found C (58.11), H (4.63), N (15.01), S (8.64).

2.12. 5-Fluoro-3-[(5-{[4-(1-methylethyl)piperazin-1-yl]carbonyl}-1,3-thiazol-2-yl)methylidene]-1,3-dihydro-2H-indol-2-one (3j)

Synthesized following the general procedure using 1-(propan-2-yl)piperazine. The product was purified by recrystallization from EtOH/THF (1:1) to afford the mixture of isomerz (*E*- : *Z*- = 81 : 19) of desired compound **3j**.

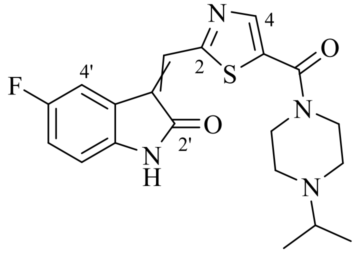

Red solid; yield 1.21 g, 44%. M.p. 203-205°C. ^{1}H NMR (301.55 MHz, DMSO-d$_6$) δ 10.91 (br s, NH *Z*-), 10.73 (br s, NH *E*-), 8.93 (dd, J_{HF} 9.6 Hz, $^4J_{HH}$ 2.4 Hz, H4' *E*-), 8.50 (s, H4 *E*-), 8.29 (s, H4 *Z*-), 8.15 (s, CH *Z*-), 7.84 (dd, J_{HF} 9.0 Hz, $^4J_{HH}$ 2.1 Hz, H4' *Z*-), 7.66 (s, CH *E*-), 7.20–7.08 (m, 1H, H6'), 6.85 (dd, $^3J_{HH}$ 8.7 Hz, J_{HF} 4.8 Hz, 1H, H7'), 3.65–3.55 (m, 4H, CH$_2$), 2.69 (sept, *J* 6.6 Hz, 1H, NCH), 2.50–2.40 (m, 4H, CH$_2$), 0.96 (d, *J* 6.6 Hz, 6H, CH$_3$). ^{13}C NMR (75.83 MHz, DMSO-d$_6$) δ 169.7 (C2' *E*-), 168.1 (C2' *Z*-), 164.4 (C2 *E*-), 161.7 (C2 *Z*-), 161.2 (C5-CO *Z*-), 160.5 (C5-CO *E*-),159.0 (d, *J* 235.2 Hz, C5' *Z*-

), 158.4 (d, *J* 233.0 Hz, C5' *E*-), 146.6 (C4 *E*-), 144.8 (C4 *Z*-), 141.2 (C7a' *E*-), 138.8 (C7a' *Z*-), 137.9 (C5 *E*-), 137.8 (C5 *Z*-), 130.0 (d, *J* 2.2 Hz, C3' *E*-), 129.4 (d, *J* 3.2 Hz, C3' *Z*-), 129.3 (CH *Z*-), 125.3 (d, *J* 9.3 Hz, C3a' *Z*-), 124.6 (CH *E*-), 122.8 (d, *J* 9.4 Hz, C3a' *E*-), 119.0 (d, *J* 24.0 Hz, C6' *E*-), 117.9 (d, *J* 25.0 Hz, C6' *Z*-), 115.0 (d, *J* 28.5 Hz, C4' *E*-), 111.7 (d, *J* 9.4 Hz, C7' *Z*-), 111.4 (d, *J* 8.0 Hz, C7' *E*-), 109.6 (d, *J* 25.5 Hz, C4' *Z*-), 54.7 (CH), 49.0 (br, NCH$_2$), 43.7 (br, NCH$_2$), 11.0 (CH$_3$). UV/Vis (DMSO, nm (mol^{-1}dm^{3}cm^{-1})) λ_{max} (ε) 434 (4150), 364 (23400), 270 (13600). LC/MS (I, %) 401.2 (100) [*M*+H]$^+$. Anal. calcd. for $C_{20}H_{21}FN_4O_2S$ C (59.98), H (5.29), N (13.99), S (8.01), found C (60.03), H (5.31), N (13.96), S (7.98).

2.13. 3-[(5-{[4-(Dimethylamino)piperidin-1-yl]carbonyl}-1,3-thiazol-2-yl)methylidene]-5-fluoro-1,3-dihydro-2H-indol-2-one (3k)

Synthesized following the general procedure using *N,N*-dimethylpiperidin-4-amine. The product was purified by re-crystallization from EtOH/THF (1:1) to afford the mixture of isomerz (*E*- : *Z*- = 82 : 18) of desired compound **3k**.

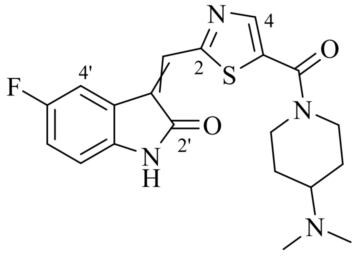

Red solid; yield 1.44 g, 52%. M.p. 97-99°C. ^{1}H NMR (301.55 MHz, DMSO-d$_6$) δ 10.94 (br s, NH *Z*-), 10.76 (br s, NH *E*-), 8.94 (dd, J_{HF} 9.6 Hz, $^4J_{HH}$ 2.1 Hz, H4' *E*-), 8.50 (s, H4 *E*-), 8.29 (s, H4 *Z*-), 8.16 (s, CH *Z*-), 7.84 (dd, J_{HF} 8.4 Hz, $^4J_{HH}$ 2.1 Hz, H4' *Z*-), 7.66 (s, CH *E*-), 7.20–7.08 (m, 1H, H6'), 6.86 (dd, $^3J_{HH}$ 8.4 Hz, J_{HF} 4.8 Hz, 1H, H7'), 4.55–3.97 (m, 2H, CH$_2$), 3.30–2.73 (m, 2H, CH$_2$), 2.41–2.35 (m, 1H, CH), 2.18 (br s, 6H, N(CH$_3$)$_2$), 1.85–1.75 (m, 2H, CH$_2$), 1.55–1.33 (m, 2H, CH$_2$). ^{13}C NMR (75.83 MHz, DMSO-d$_6$) δ 169.7 (C2' *E*-), 168.1 (C2' *Z*-), 164.3 (C2 *E*-), 161.6 (C2 *Z*-), 161.1 (C5-CO *Z*-), 160.4 (C5-CO *E*-),159.0 (d, *J* 235.6 Hz, C5' *Z*-), 158.4 (d, *J* 234.2 Hz, C5' *E*-), 146.3 (C4 *E*-), 144.6 (C4 *Z*-), 141.2 (C7a' *E*-), 138.9 (C7a' *Z*-), 138.1 (C5), 130.0 (d, *J* 3.2 Hz, C3' *E*-), 129.4 (CH *Z*-), 129.3 (d, *J* 3.7 Hz, C3' *Z*-), 125.4 (d, *J* 9.1 Hz, C3a' *Z*-), 124.6 (CH *E*-), 122.8 (d, *J* 10.4 Hz, C3a' *E*-), 119.0 (d, *J* 24.0 Hz, C6' *E*-), 117.9 (d, *J* 24.6 Hz, C6' *Z*-), 115.0 (d, *J* 26.2 Hz, C4' *E*-), 111.8 (d, *J* 8.8 Hz, C7' *Z*-), 111.4 (d, *J* 9.3 Hz, C7' *E*-), 109.7 (d, *J* 24,7 Hz, C4' *Z*-), 61.9 (CH), 47.4 (br, NCH$_2$), 45.9 (2C, CH$_2$), 42.2 (2C, N(CH$_3$)$_2$), 29.1 (br, NCH$_2$). UV/Vis (DMSO, nm (mol^{-1}dm^{3}cm^{-1})) λ_{max} (ε) 433 (3760), 376 (17300), 359 (19600), 264 (12100). LC/MS (I, %) 401.2 (100) [*M*+H]$^+$, RT 0.717 min; 401.2 (100) [*M*+H]$^+$, RT 0.736 min. Anal. calcd. for $C_{20}H_{21}FN_4O_2S$ C (59.98), H (5.29), N (13.99), S (8.01), found C (60.02), H (5.25), N (14.02), S (8.05).

3. SUPPLEMENTARY TABLES AND FIGURES

3.1. Supplementary tables

Table S2. Growth inhibition NCI-60 cancer cell lines of tested compounds, GI, %

**Panel**/Cell Line	**Compounds / GI**											
	**2**	**3a**	**3b**	**3c**	**3d**	**3e**	**3f**	**3g**	**3h**	**3i**	**3j**	**3k**
**Leukemia**												
CCRF-CEM	98.81	109.56	87.78	92.80	96.43	26.79	52.13	25.37	73.26	78.98	87.64	88.87
HL-60(TB)	137.08	74.72	64.39	58.83	83.36	40.26	*43.29*	19.79	52.16	62.52	71.65	67.80
K-562	100.13	83.47	58.97	56.57	73.38	43.32	*34.71*	*36.28*	58.33	63.69	64.22	62.74

MOLT-4	110.55	95.76	86.60	109.63	106.95	32.87	*34.60*	16.97	93.44	93.73	104.26	85.03
RPMI-8226	101.78	114.15	97.65	94.86	95.49	40.44	*43.34*	28.51	70.45	80.74	74.69	81.61
SR	106.89	ND	ND	ND	105.16	88.14	13.40	15.51	51.30	ND	87.44	87.56
**NSCLC**												
A549/ATCC	104.89	91.73	87.76	85.85	101.74	86.28	83.77	71.35	85.48	88.55	94.75	95.69
EKVX	108.95	89.57	76.39	78.17	93.04	96.00	70.80	52.24	80.41	71.37	78.36	81.22
HOP-62	113.30	89.60	91.75	85.97	90.20	65.32	60.19	63.38	81.00	84.30	87.41	84.49
HOP-92	108.11	87.96	79.95	81.23	87.52	48.10	74.68	**4.05**	59.62	80.05	83.37	70.57
NCI-H226	105.70	84.56	77.92	72.62	82.22	67.03	72.99	*45.90*	65.35	89.75	74.65	67.12
NCI-H23	115.09	89.69	77.09	82.19	86.36	65.31	71.49	*43.62*	67.87	78.06	73.88	70.80
NCI-H322M	111.22	102.93	81.88	88.92	95.31	71.15	78.19	80.08	82.88	89.96	92.41	97.84
NCI-H460	81.87	106.13	94.59	85.38	107.61	85.50	69.50	*35.87*	89.72	85.16	113.90	112.99
NCI-H522	104.89	91.59	83.02	72.11	93.65	86.28	*38.64*	23.28	82.31	76.01	87.88	82.68
**Colon Cancer**												
COLO 205	117.74	92.65	85.64	93.55	101.61	113.77	96.32	71.61	94.97	99.69	92.93	80.99
HCC-2998	115.74	101.57	98.95	110.98	98.16	99.26	96.22	90.80	94.79	95.84	103.99	97.00
HCT-116	102.62	87.89	79.26	ND	96.00	65.35	57.80	*31.46*	58.30	79.86	73.06	65.12
HCT-15	100.22	91.01	78.36	ND	92.77	68.83	*41.28*	27.76	57.48	72.72	72.27	85.18
HT29	102.49	98.55	88.28	83.25	104.78	88.26	75.90	*47.14*	93.77	95.30	102.90	81.85
KM12	100.16	89.70	75.95	73.21	95.65	63.15	58.32	54.12	74.76	82.82	95.04	87.37
SW-620	105.98	90.34	79.40	80.35	115.59	89.06	87.16	*48.40*	114.63	93.14	100.62	100.25
**CNS Cancer**												
SF-268	87.88	80.32	80.86	73.49	96.70	55.79	85.37	59.96	88.05	79.08	104.08	86.17
SF-295	101.21	94.86	96.25	92.57	98.45	73.60	66.47	*32.31*	74.22	96.36	97.05	98.32

SF-539	99.12	90.70	94.32	92.46	83.24	63.94	65.30	*47.79*	80.16	88.79	94.43	87.42
SNB-19	98.11	97.33	92.25	83.18	98.58	111.21	72.19	77.84	99.98	84.48	106.46	108.60
SNB-75	87.88	103.21	87.25	90.23	106.79	55.79	59.41	**10.09**	89.04	84.30	79.80	93.60
U251	101.21	88.14	86.44	75.27	91.14	73.60	57.42	*45.97*	69.29	78.02	74.65	70.61
**Melanoma**												
LOX IMVI	94.64	86.50	79.43	80.53	93.47	47.95	57.31	24.79	71.80	75.31	85.59	76.49
MALME-3M	109.53	106.23	101.74	135.81	110.59	90.82	76.30	79.68	169.50	115.92	103.37	120.08
M14	110.62	86.13	80.80	76.48	94.42	80.55	64.39	58.96	75.63	79.47	80.05	78.45
MDA-MB-435	119.48	81.10	67.37	59.54	95.08	100.52	**4.60**	21.95	62.20	71.01	82.02	69.43
SK-MEL-2	93.62	100.40	104.47	101.70	115.10	91.00	58.94	*31.94*	81.95	100.87	106.05	99.23
SK-MEL-28	103.60	93.51	105.75	100.86	125.23	70.84	69.46	67.55	89.31	94.98	105.45	101.11
SK-MEL-5	98.99	83.96	78.36	72.62	103.63	76.28	79.44	60.45	88.70	76.30	108.05	102.18
UACC-257	140.95	94.69	96.99	90.67	109.91	26.00	76.64	84.55	92.54	85.95	113.20	115.45
UACC-62	116.75	ND	ND	ND	95.97	69.47	*50.38*	55.52	96.12	ND	96.29	104.22
**Ovarian Cancer**												
IGROV1	84.54	125.18	75.45	97.70	95.77	81.71	73.57	66.47	106.73	106.59	108.79	112.34
OVCAR-3	103.50	91.35	ND	75.21	108.36	69.23	*37.99*	12.98	70.19	81.07	81.15	81.38
OVCAR-4	100.74	97.95	88.56	80.06	87.23	35.42	*41.21*	28.04	61.84	84.69	84.63	71.75
OVCAR-5	98.16	89.87	109.69	107.45	113.01	77.70	97.60	74.54	84.07	104.78	95.68	102.13
OVCAR-8	96.68	91.33	87.57	85.07	94.59	60.48	69.66	51.54	80.12	78.78	88.18	91.98
NCI/ADR-RES	107.61	94.15	93.80	86.10	93.64	75.42	28.41	**4.87**	53.42	74.86	65.54	83.57
SK-OV-3	103.94	95.90	88.05	78.44	99.73	85.30	66.59	57.58	89.78	86.10	106.03	103.78
**Renal Cancer**												
786-0	96.46	79.55	78.61	74.88	94.83	71.99	81.02	67.78	82.86	82.10	85.31	82.08

A498	100.64	105.29	94.27	96.04	105.44	45.31	96.82	79.45	83.87	99.40	87.36	99.76
ACHN	91.30	96.68	90.48	85.80	104.54	74.15	*49.39*	51.14	82.70	82.96	93.70	83.09
CAKI-1	94.49	90.97	92.46	74.81	79.59	68.84	<u>17.19</u>	<u>15.16</u>	*46.27*	80.67	58.91	*43.44*
RXF 393	93.32	69.22	63.66	60.60	90.12	65.75	64.10	*42.28*	73.36	70.45	84.48	73.49
SN12C	110.10	99.16	99.60	94.50	96.74	76.63	81.52	73.22	98.35	86.99	116.09	120.40
TK-10	96.46	94.68	105.10	104.82	149.41	71.99	80.57	96.41	101.17	101.98	106.55	158.80
UO-31	100.64	86.10	75.47	59.13	74.61	45.31	61.88	*42.68*	70.45	55.63	57.30	55.31
**Prostate Cancer**												
PC-3	109.93	83.95	74.05	76.78	81.85	76.73	66.11	54.60	79.54	75.89	83.04	77.95
DU-145	109.61	86.32	74.67	68.79	95.97	81.45	52.61	*50.42*	69.94	78.25	93.70	88.12
**Breast Cancer**												
MCF7	96.91	78.62	69.47	54.99	82.06	45.56	<u>29.18</u>	<u>11.81</u>	55.34	57.50	51.76	61.19
MDA-MB-231/ATCC	110.91	113.19	94.93	106.91	96.21	94.60	73.57	61.46	89.24	102.72	103.49	100.49
HS 578T	79.52	99.09	ND	80.41	98.73	29.90	78.70	94.80	110.67	83.65	102.27	113.40
BT-549	98.99	75.15	80.66	75.89	106.05	47.49	60.62	<u>27.56</u>	66.31	78.16	93.71	87.08
T-47D	96.91	ND	ND	ND	74.06	45.56	<u>25.41</u>	**3.83**	*39.91*	ND	66.13	57.81
MDA-MB-468	110.91	81.05	74.30	*50.36*	86.10	94.60	55.15	<u>24.66</u>	66.57	63.03	86.16	65.42

3.2. Supplementary Figures

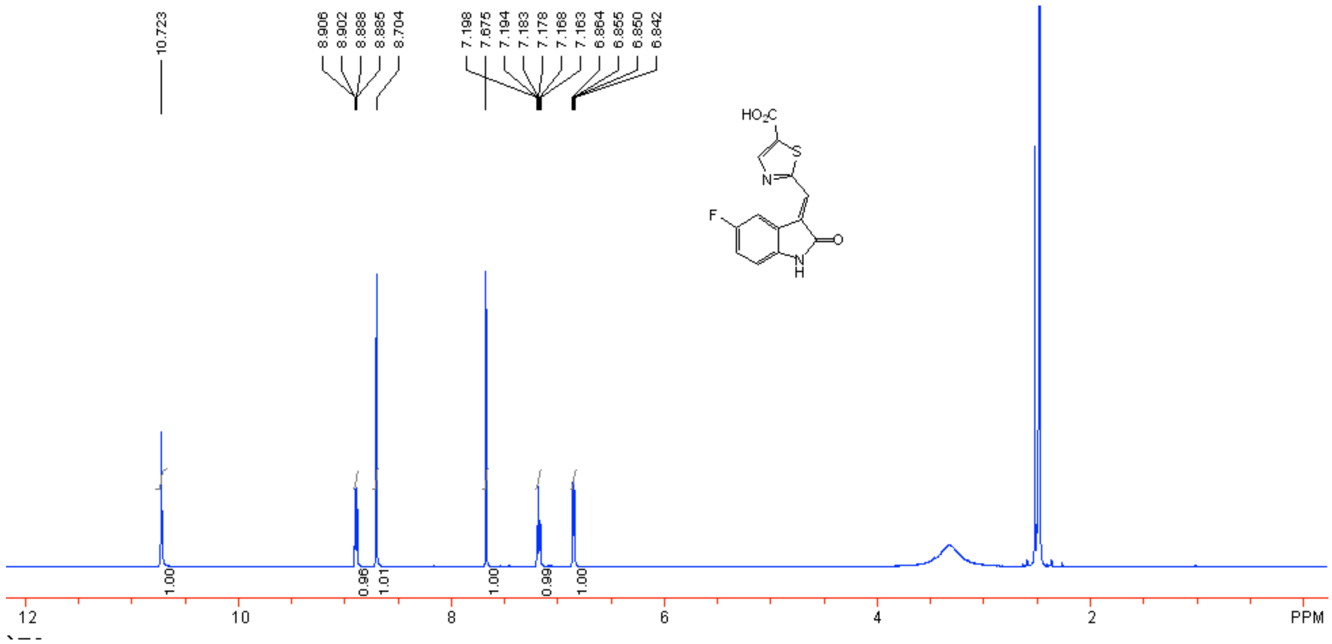

Fig. (S1). ^{1}H-NMR spectrum (DMSO-d_6) of compound **2**.

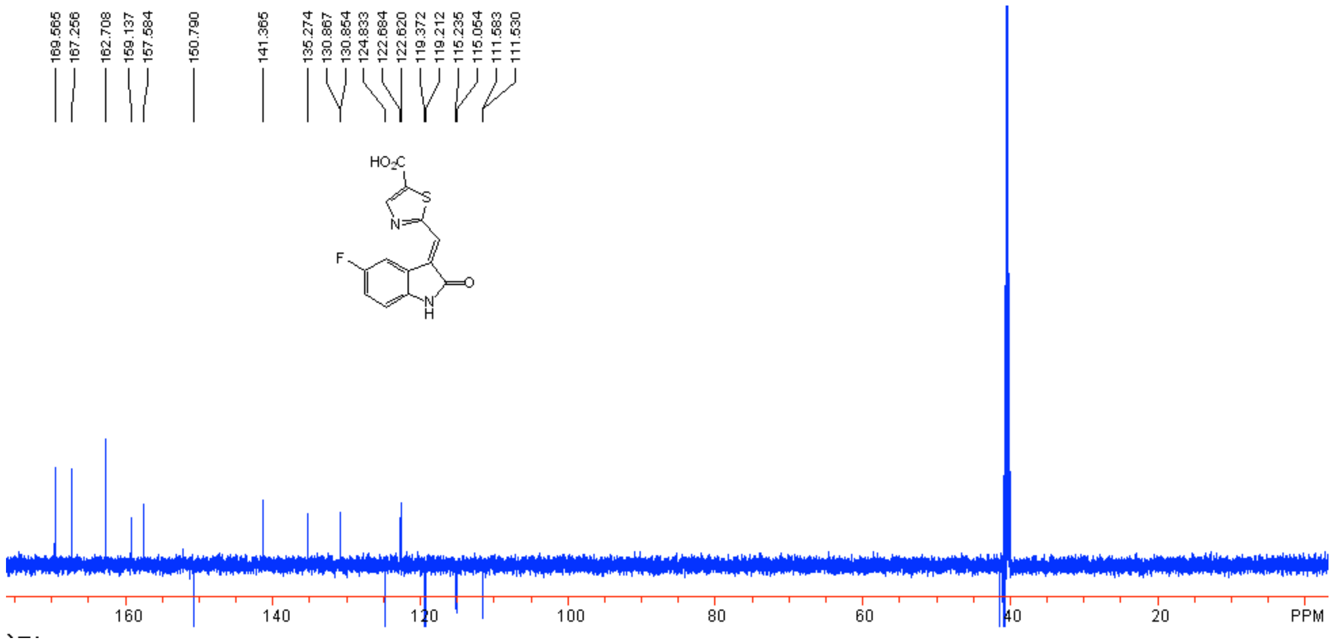

Fig. (S2). ^{13}C-NMR spectrum (DMSO-d_6) of compound **2**.

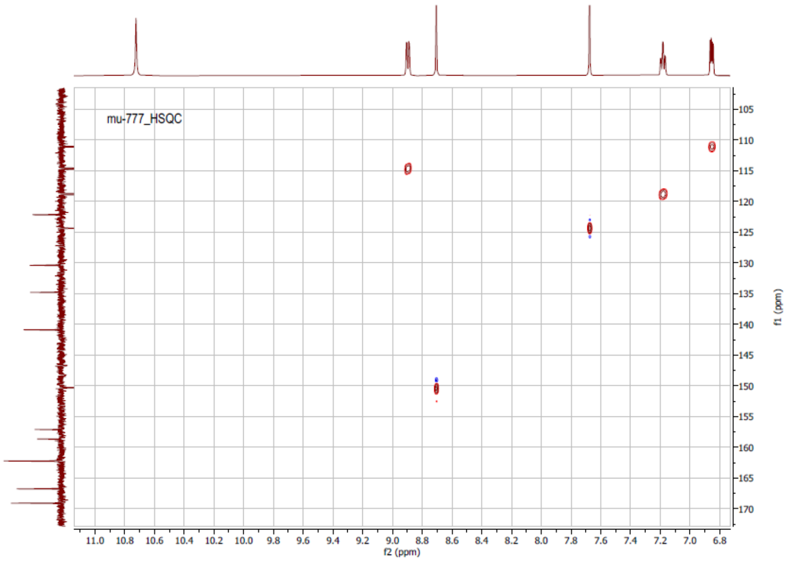

Fig. (S3). HSQC spectrum (DMSO-d_6) of compound **2**.

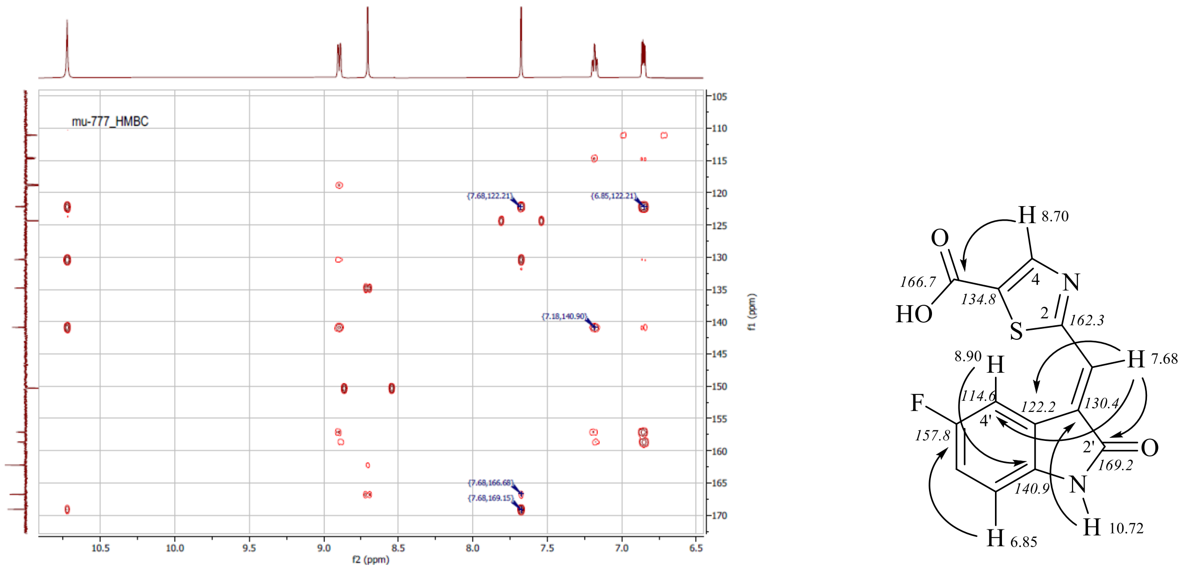

Fig. (S4). HMBC spectrum (DMSO-d_6) and corelations for compound **2**.

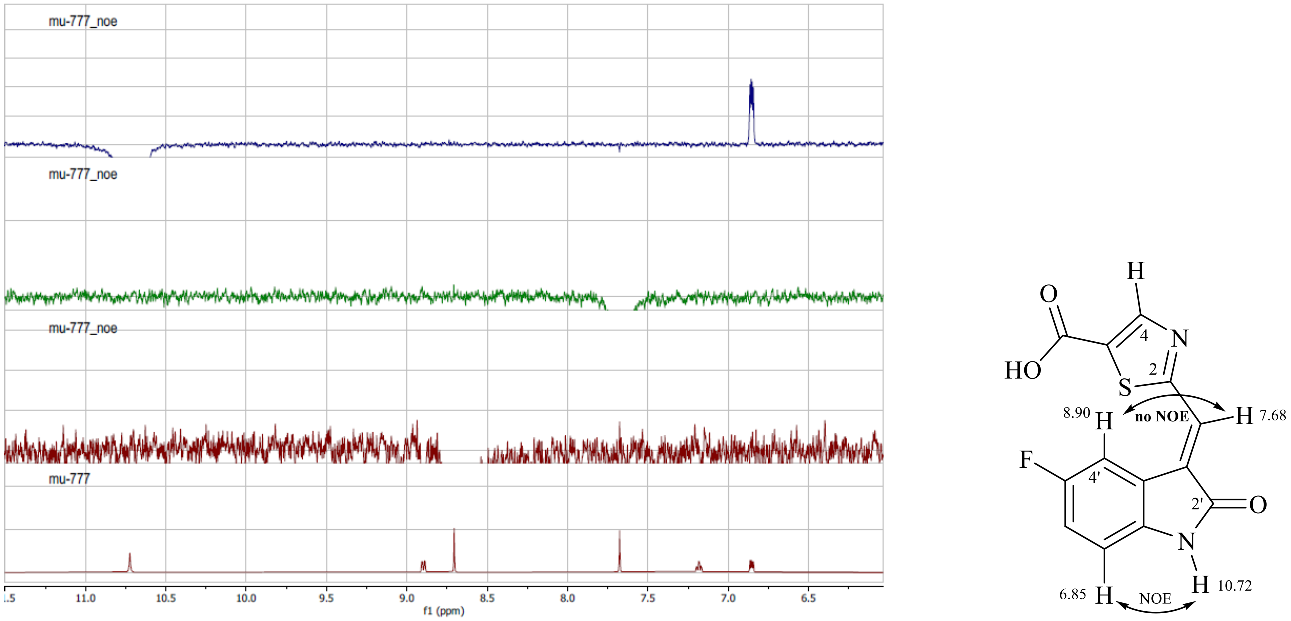

Fig. (S5). NOE experiment (DMSO-d$_6$) for compound **2**.

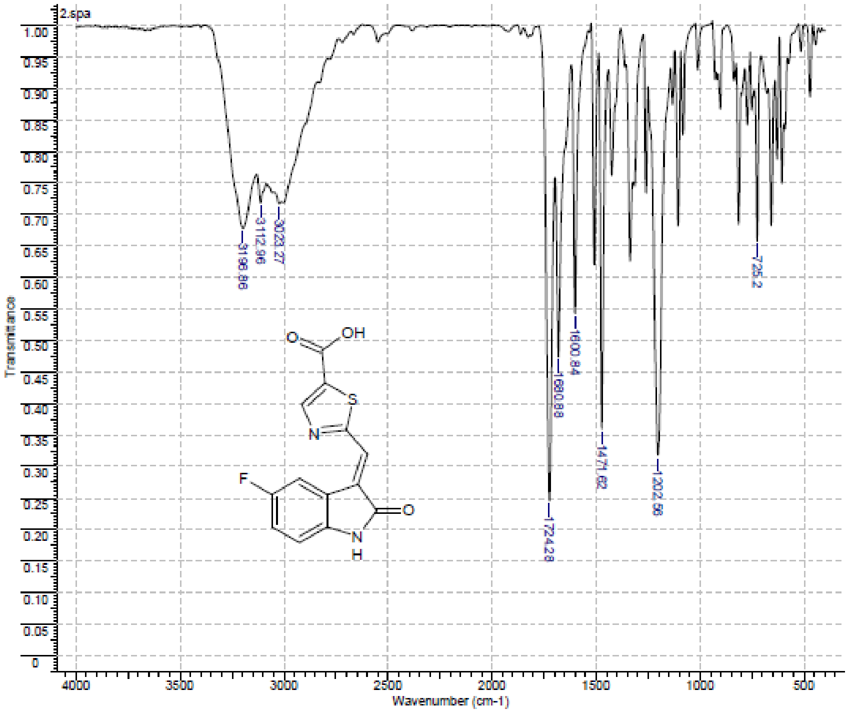

Fig. (S6). ATR-IR spectrum (KBr) of compound **2**.

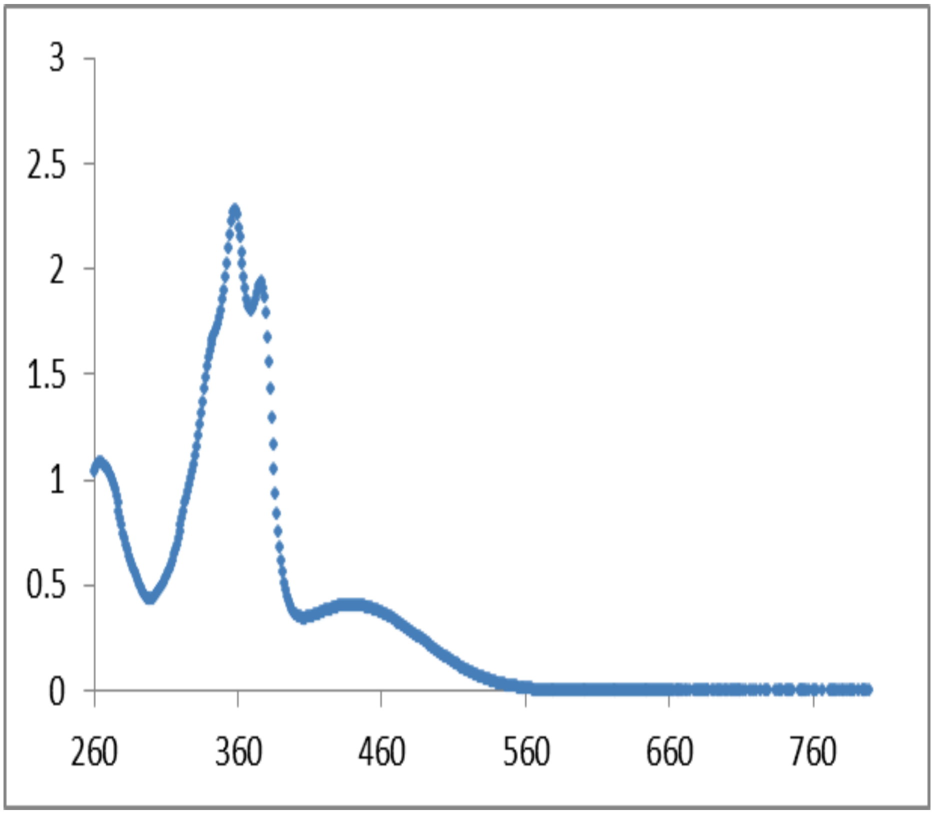

Fig. (S7). UV/Vis spectrum (DMSO) of compound **2**.

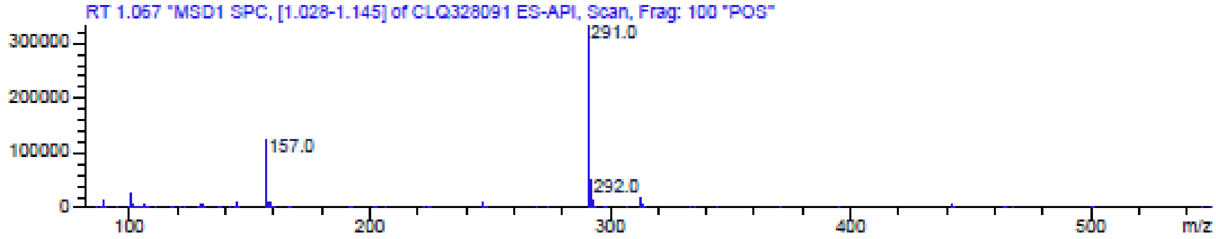

#	RT	DAD1A	DAD1B	MSD1	MSD2	ELSD	MSD1 ions	MSD1 rt	MSD2 ions	MSD2 rt	Info
1	1.061	100.0%	100.0%	100.0%	100.0%	100.0%	291.0(100)	1.067	245.0(86),187.0(6),326.8(5)	1.069	

Fig. (S8). LC/MS spectrum of compound **2**.

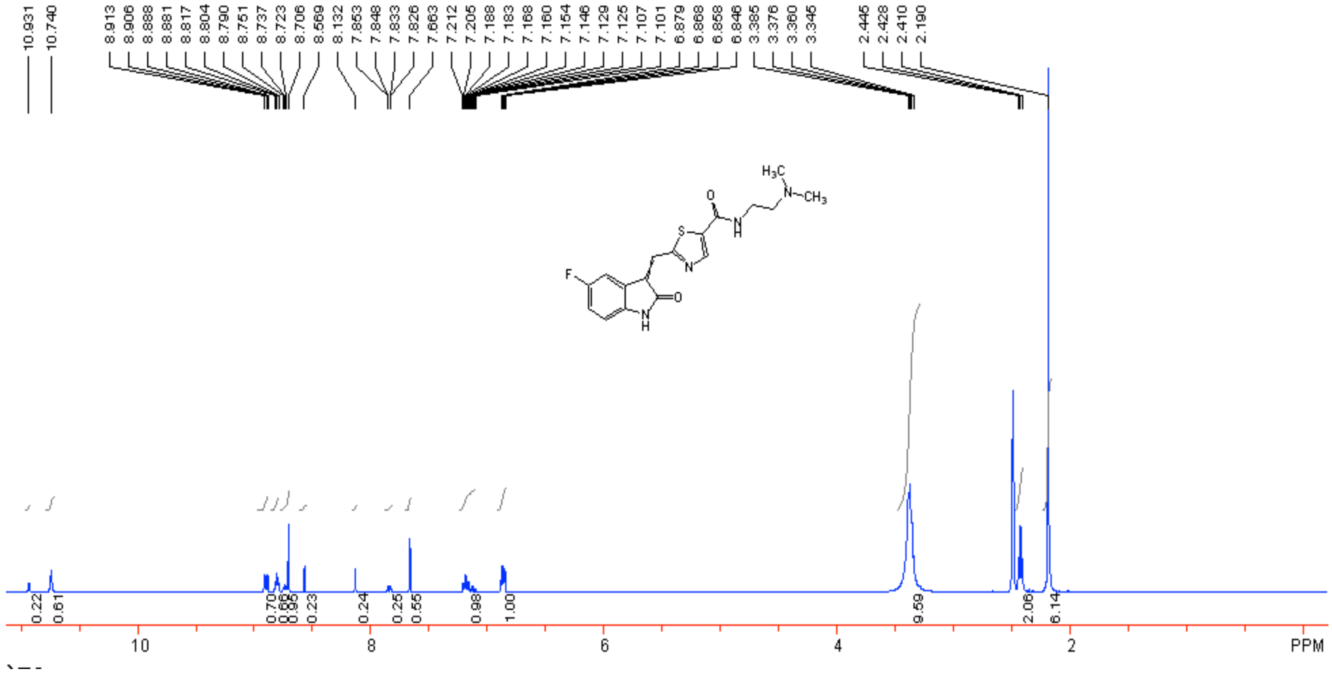

Fig. (S9). ^{1}H-NMR spectrum (DMSO-d_6) of compound **3a**.

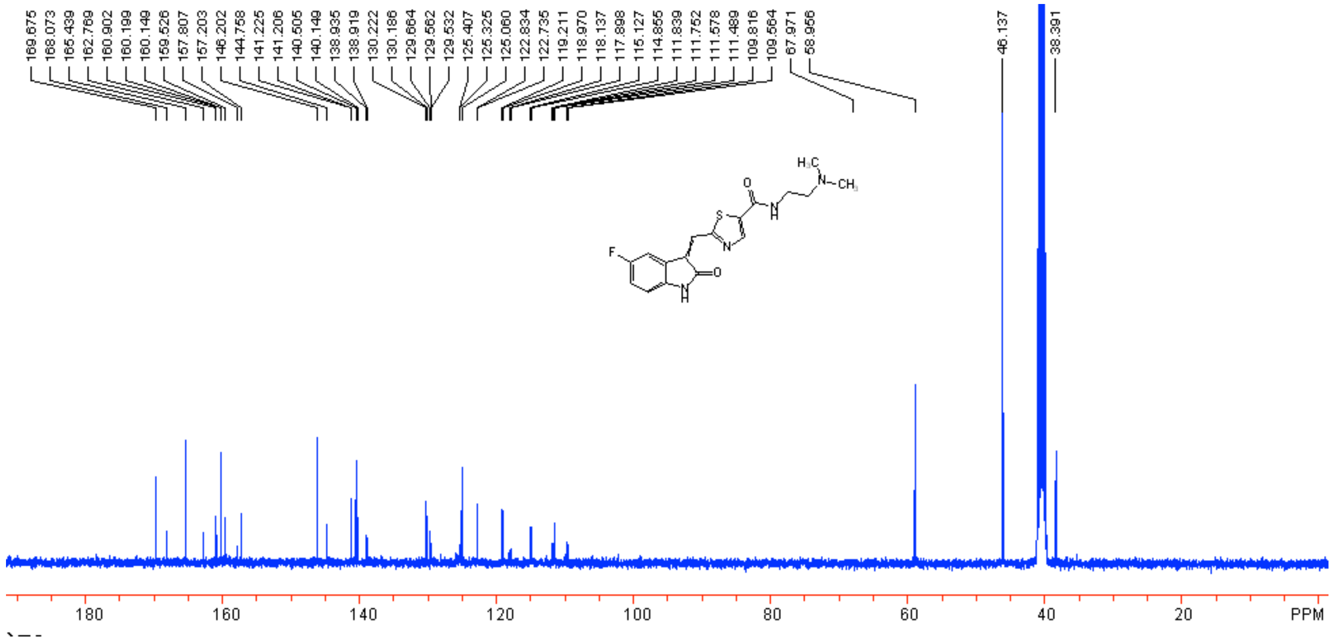

Fig. (S10). ^{13}C-NMR spectrum (DMSO-d_6) of compound **3a**.

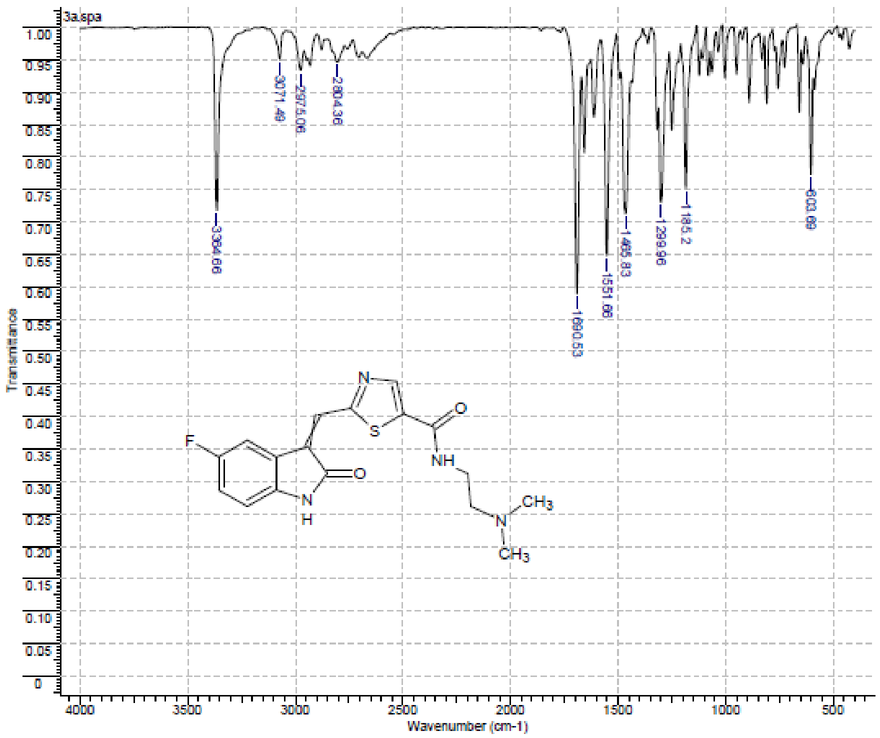

Fig. (S11). ATR-IR spectrum (KBr) of compound **3a**.

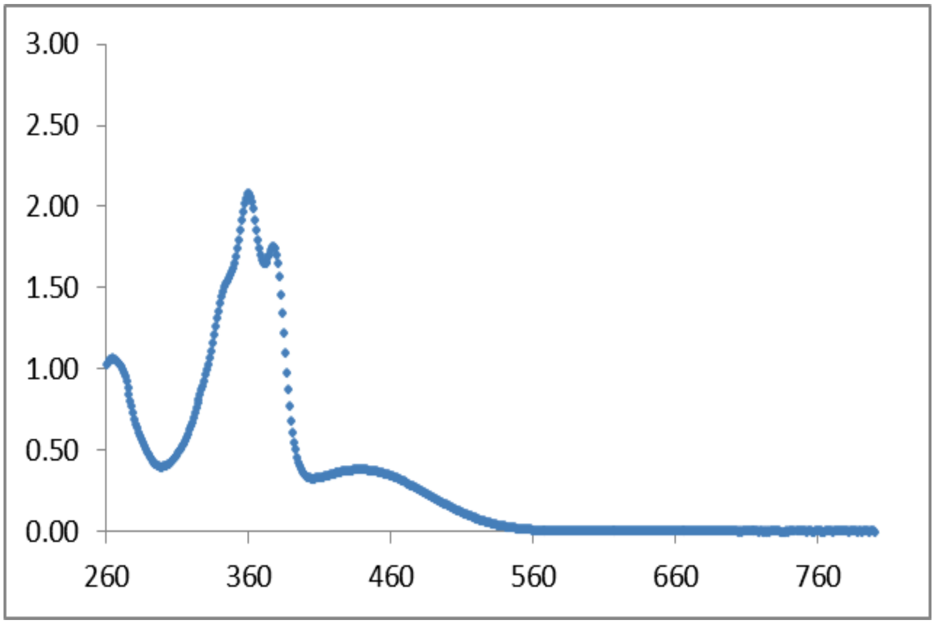

Fig. (S12). UV/Vis spectrum (DMSO) of compound **3a**.

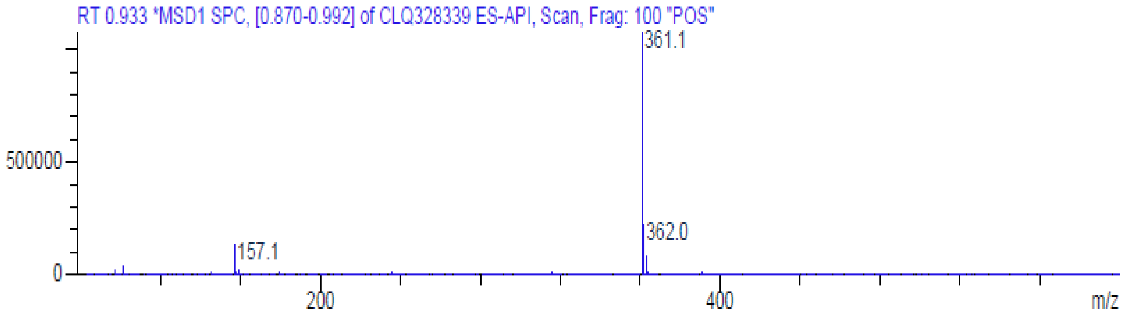

#	RT	DAD1A	DAD1B	MSD1	MSD2	ELSD	MSD1 ions	MSD1 rt	MSD2 ions	MSD2 rt	Info
	0.907	59.0%	78.8%	98.1%	74.3%	42.7%	361.1(100)	0.933	359.0(100)	0.922	

Fig. (S13). LC/MS spectrum of compound **3a**.

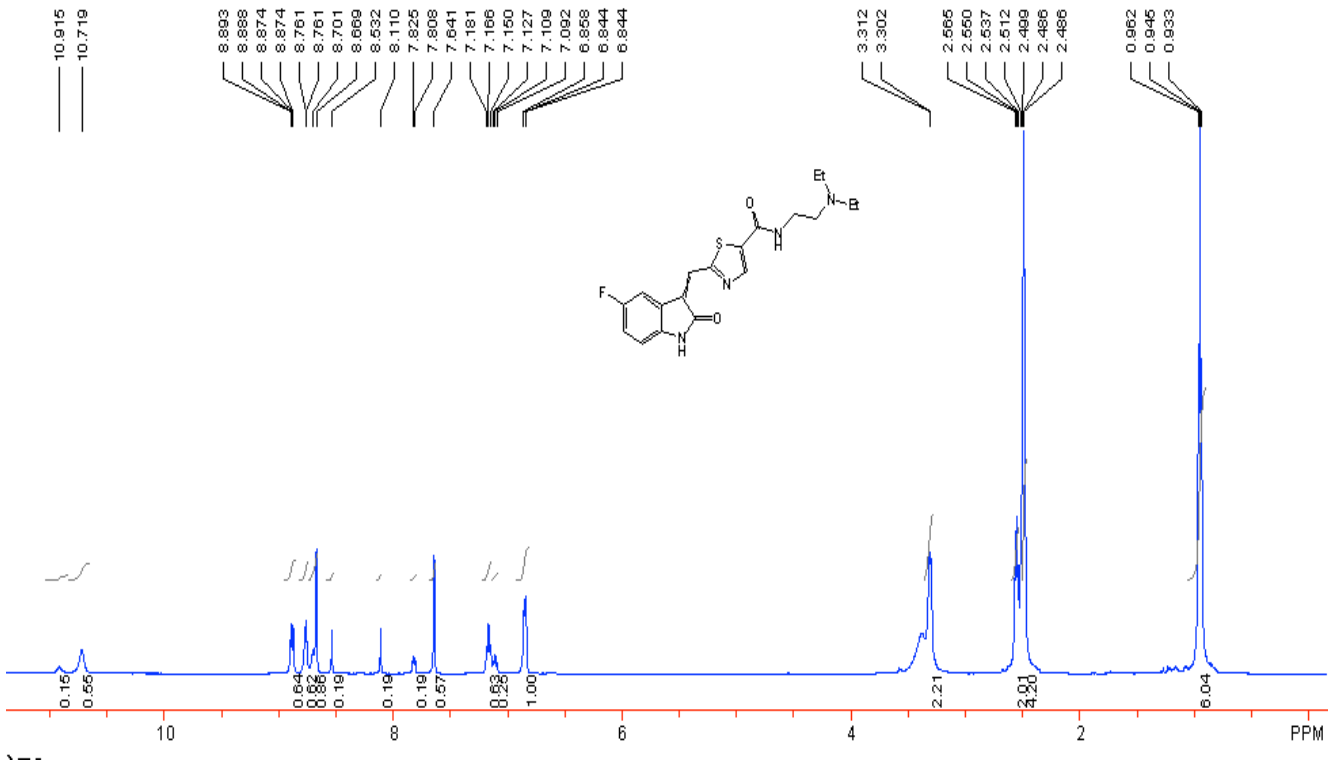

Fig. (S14). ^{1}H-NMR spectrum (DMSO-d_6) of compound **3b**.

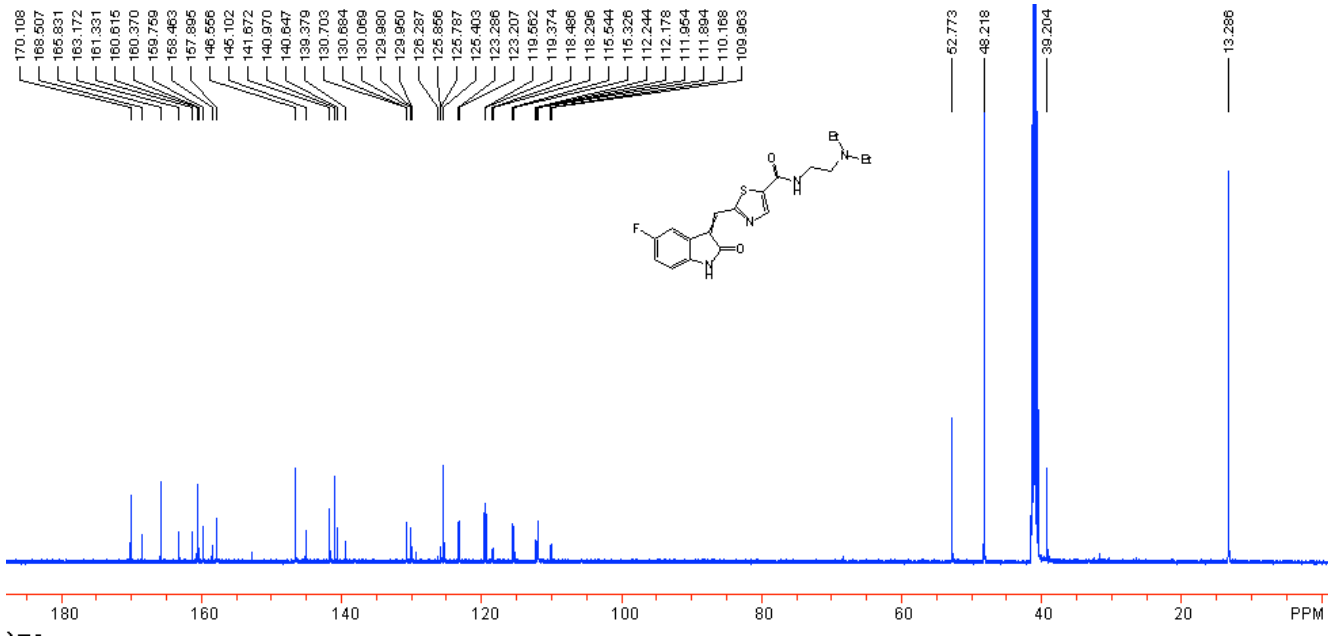

Fig (S15). ^{13}C-NMR spectrum (DMSO-d$_6$) of compound **3b**.

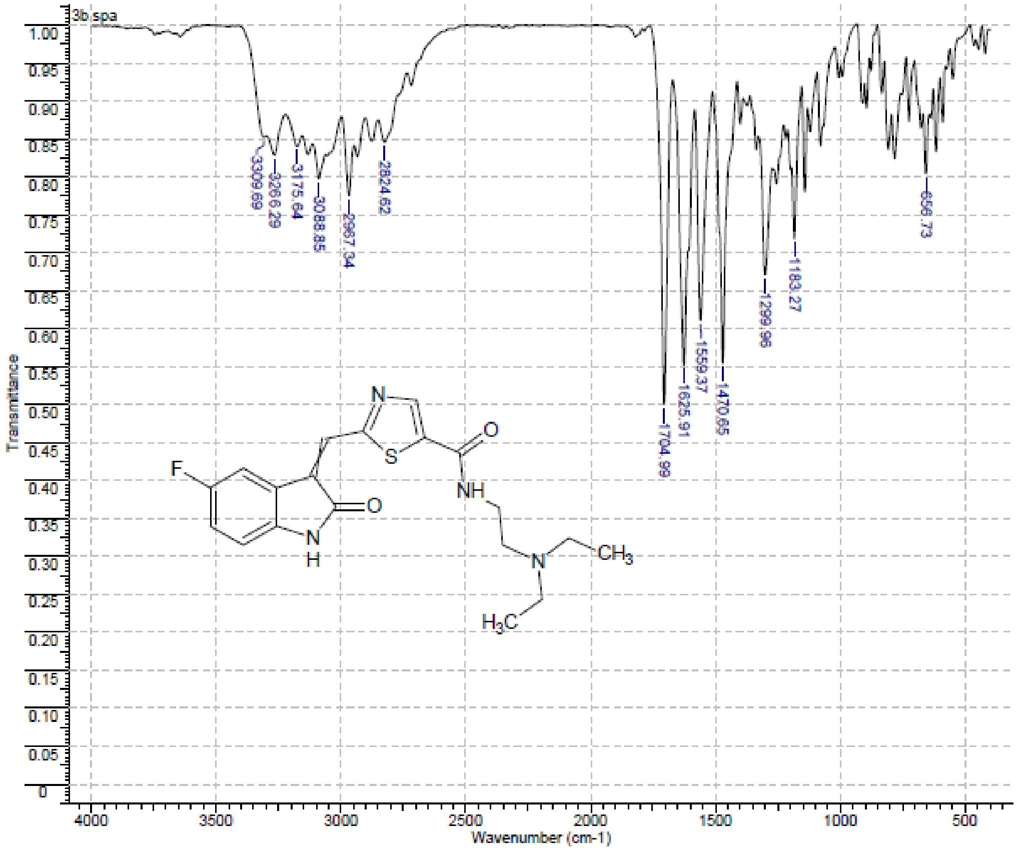

Fig. (S16). ATR-IR spectrum (KBr) of compound **3b**.

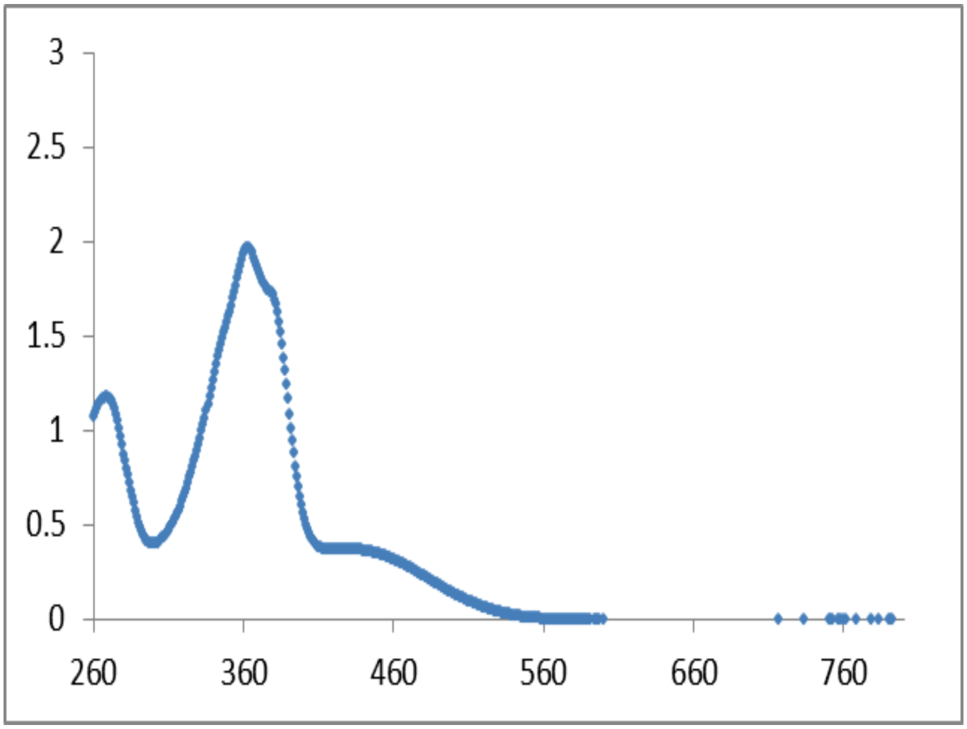

Fig. (S17). UV/Vis spectrum (DMSO) of compound **3b**.

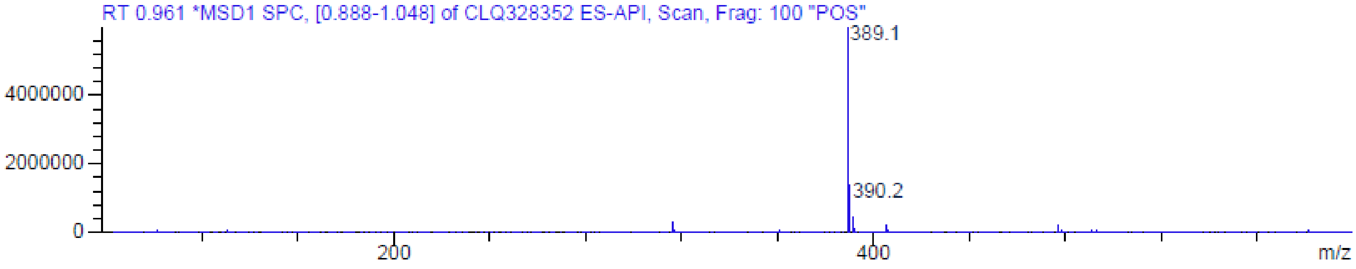

#	RT	DAD1A	DAD1B	MSD1	MSD2	ELSD	MSD1 ions	MSD1 rt	MSD2 ions	MSD2 rt	Info
	0.938	66.5%	73.1%	97.4%	89.5%	91.4%	389.1(93),316.1(4),405.2(2)	0.961	387.2(97),423.0(3)	0.967	

Fig. (S18). LC/MS spectrum of compound **3b**.

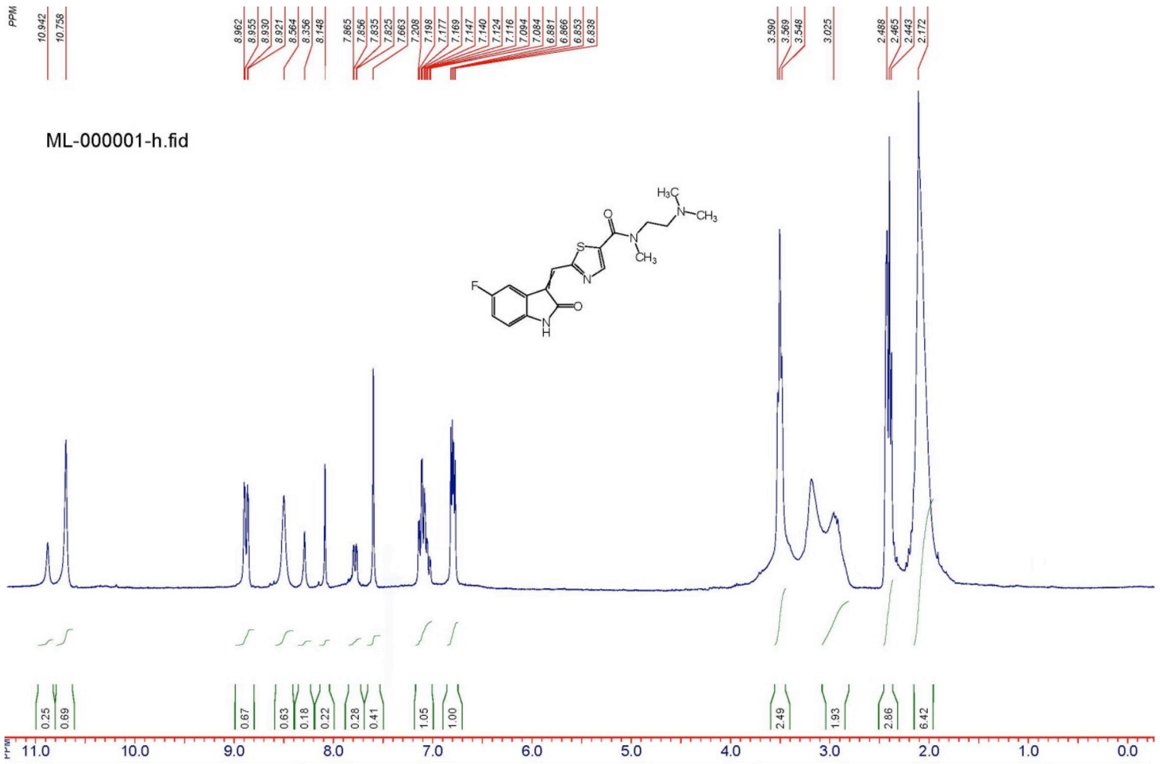

Fig. (S19). ^{1}H-NMR spectrum (DMSO-d_6) of compound **3c**.

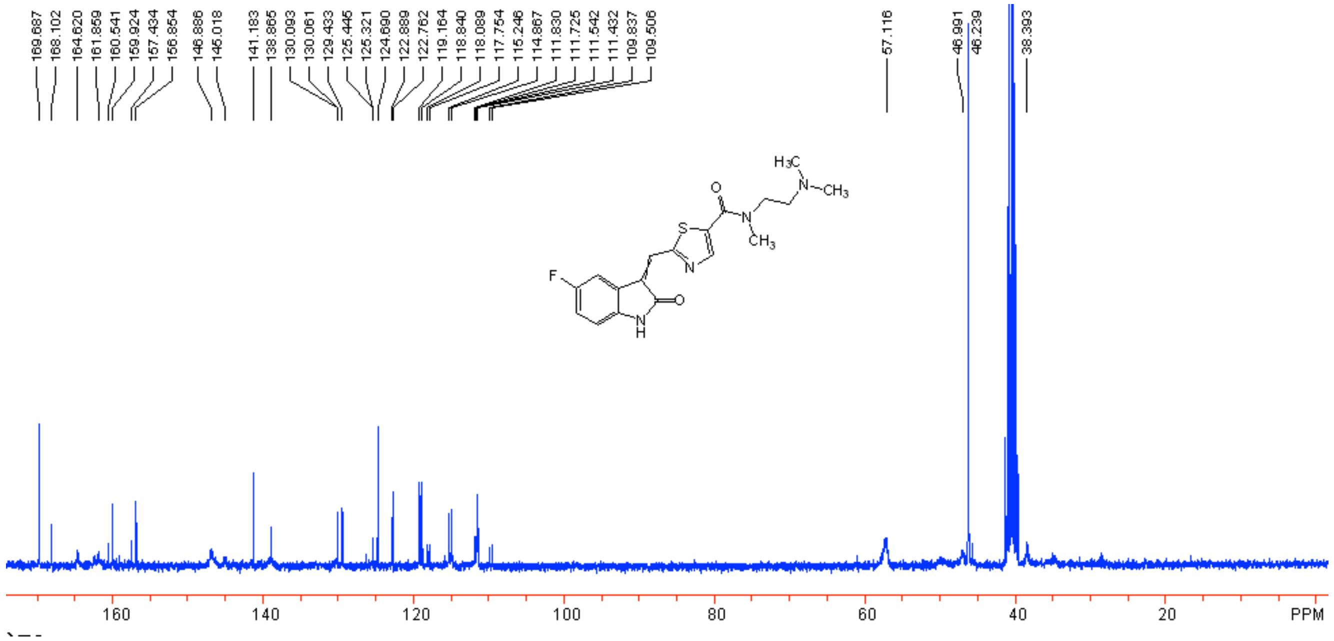

Fig. (S20). ^{13}C-NMR spectrum (DMSO-d_6) of compound **3c**.

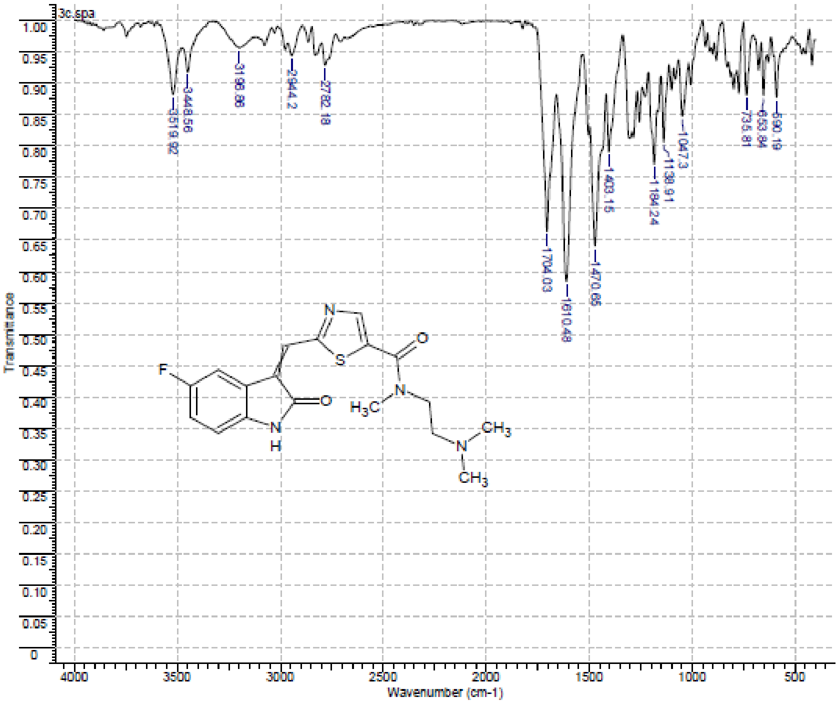

Fig. (S21). ATR-IR spectrum (KBr) of compound **3c**.

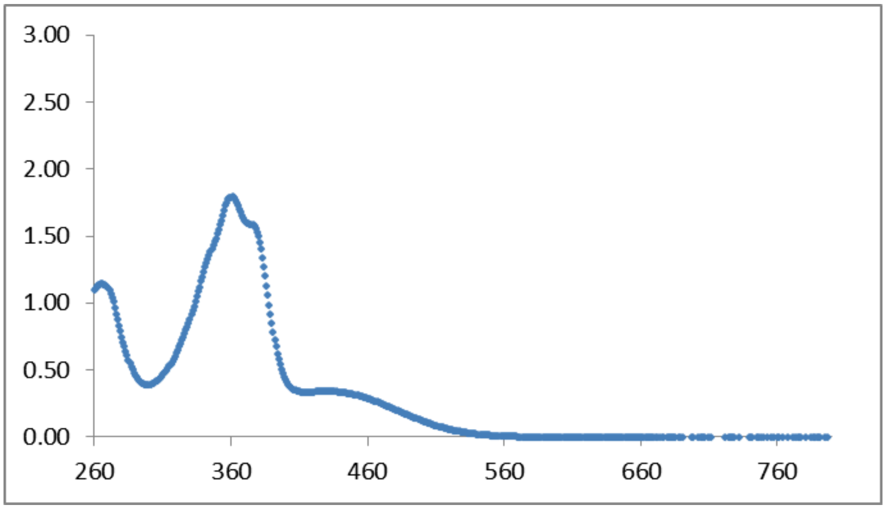

Fig. (S22). UV/Vis spectrum (DMSO) of compound **3c**.

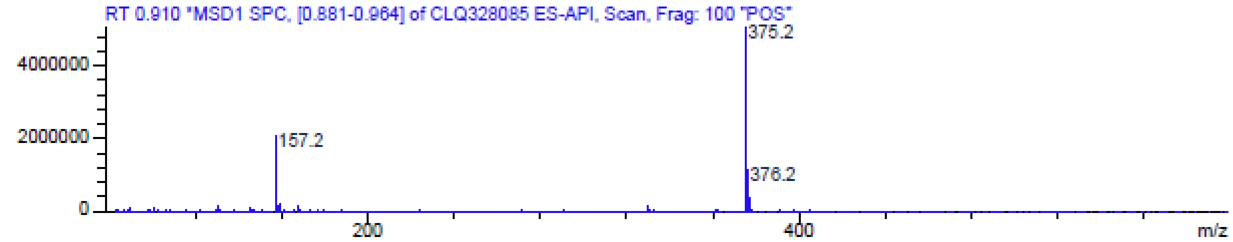

#	RT	DAD1A	DAD1B	MSD1	MSD2	ELSD	MSD1 ions	MSD1 rt	MSD2 ions	MSD2 rt	Info
	0.898	74.0%	75.8%	96.5%	---	100.0%	375.2(97),330.2(3)	0.910	---	---	

Fig. (S23). LC/MS spectrum of compound **3c**.

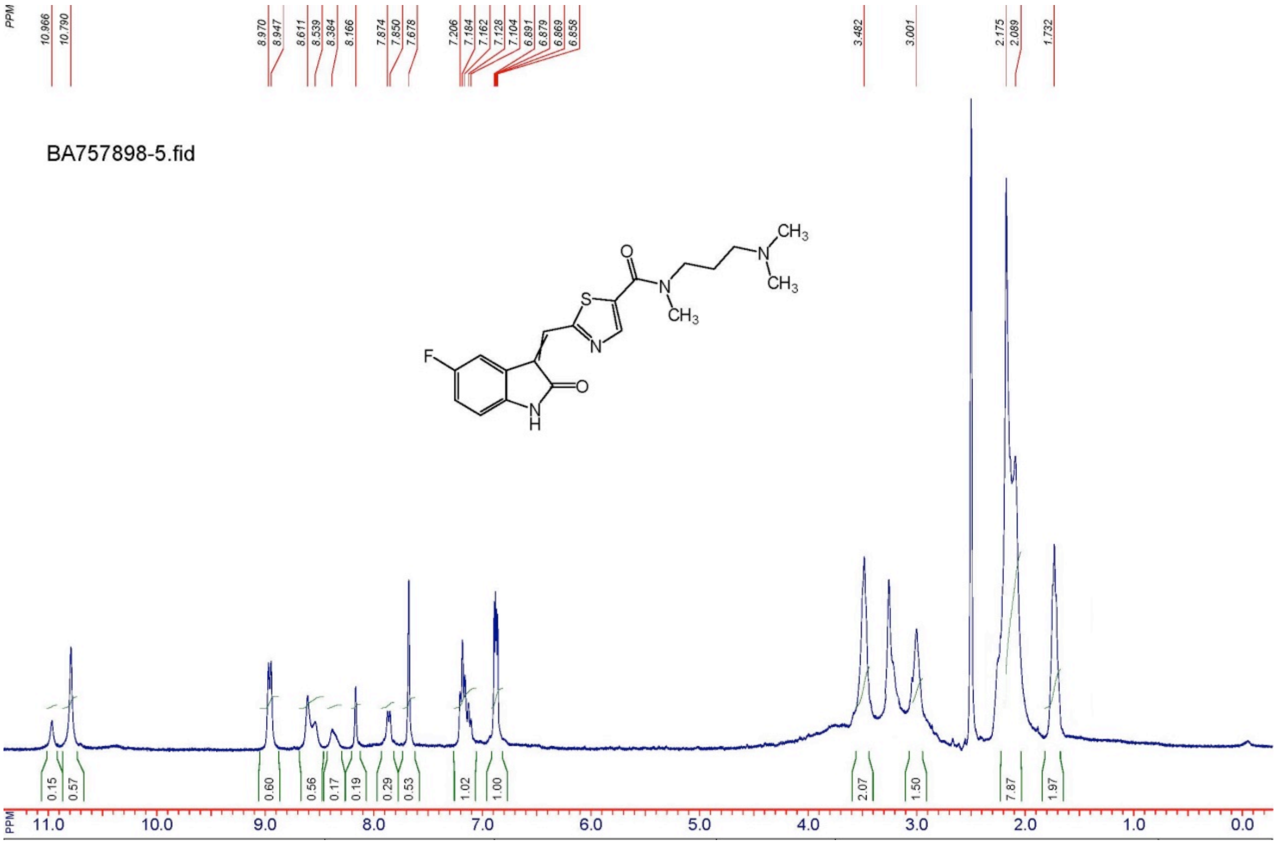

Fig. (S24). ^{1}H-NMR spectrum (DMSO-d_6) of compound **3d**.

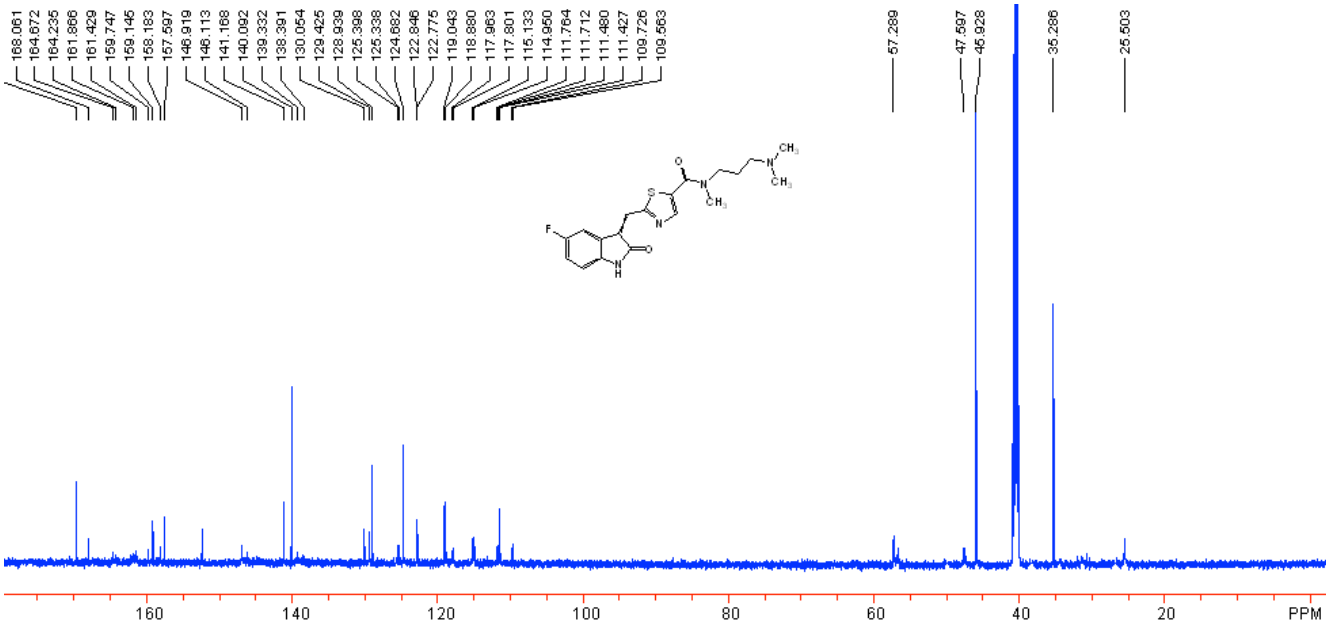

Fig. (S25). ^{13}C-NMR spectrum (DMSO-d$_6$) of compound **3d**.

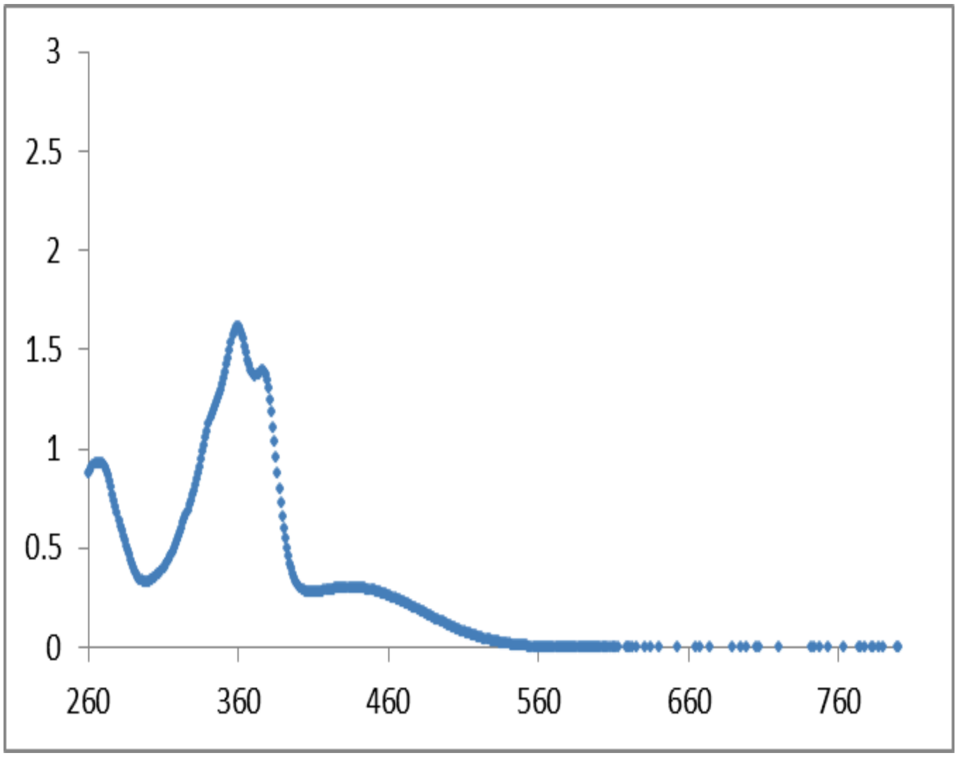

Fig. (S26). UV/Vis spectrum (DMSO) of compound **3d**.

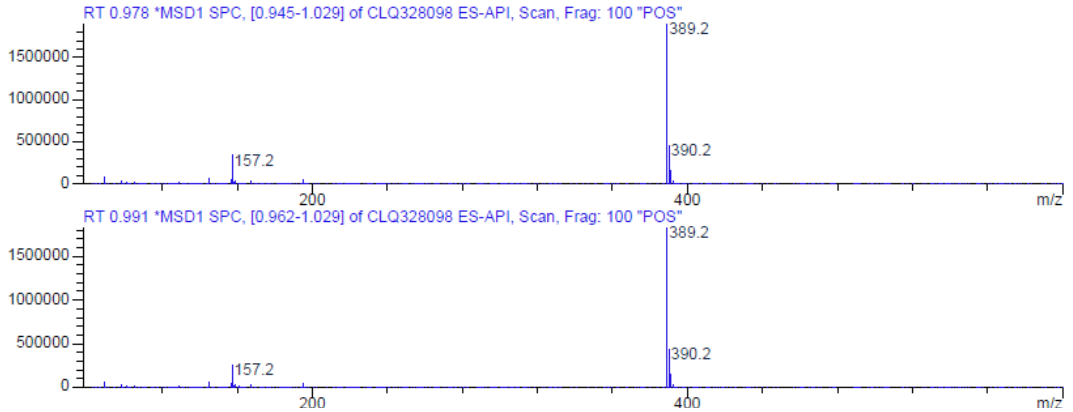

#	RT	DAD1A	DAD1B	MSD1	MSD2	ELSD	MSD1 ions	MSD1 rt	MSD2 ions	MSD2 rt	Info
4	0.966	---	---	96.5%	80.7%	---	389.2(100)	0.978	387.2(100)	0.979	
5	0.979	26.8%	23.1%	2.0%	1.2%	---	195.0(100)	0.991	423.0(100)	0.991	

Fig. (S27). LC/MS spectrum of compound **3d**.

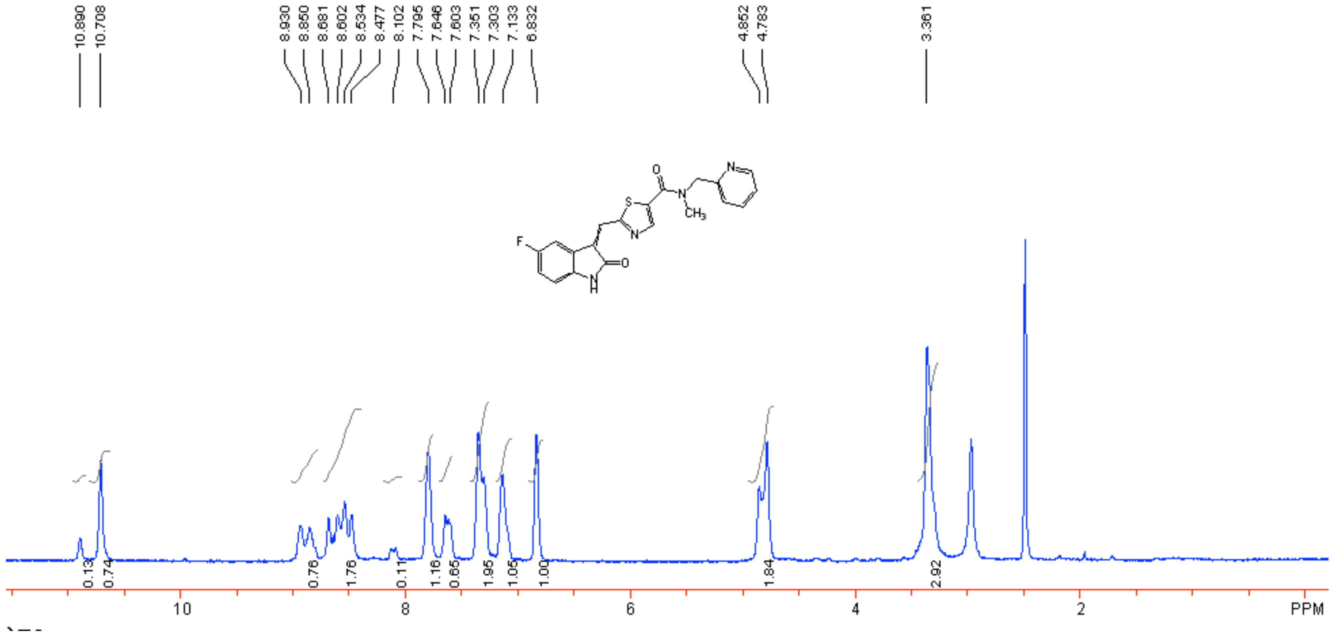

Fig. (S28). ^{1}H-NMR spectrum (DMSO-d_6) of compound **3e**.

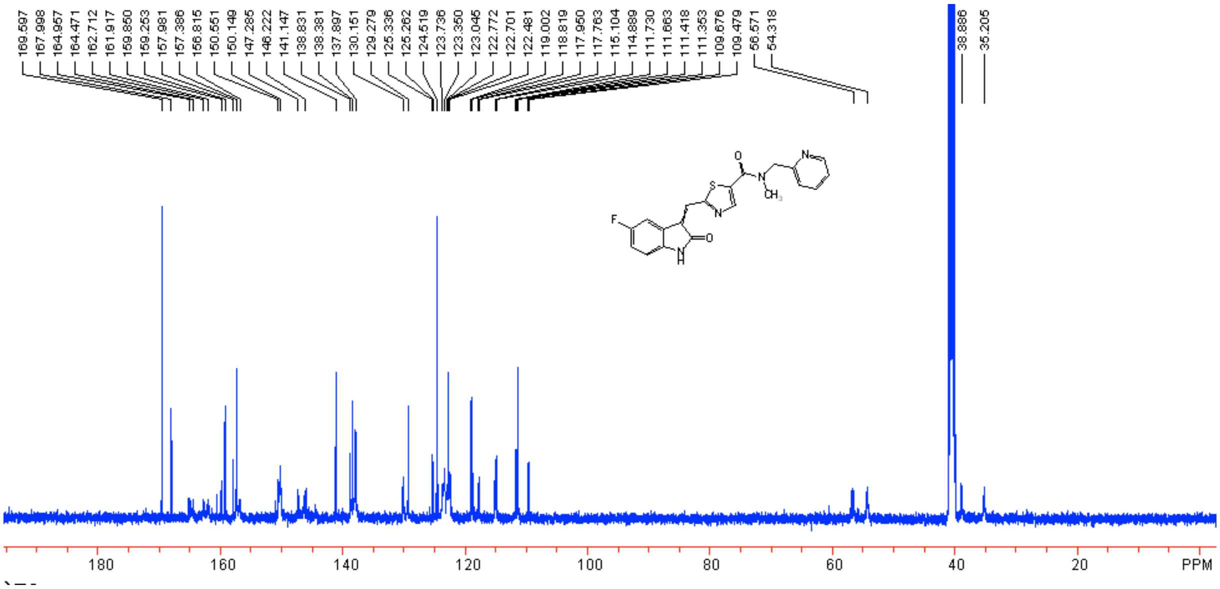

Fig. (S29). ^{13}C-NMR spectrum (DMSO-d_6) of compound **3e**.

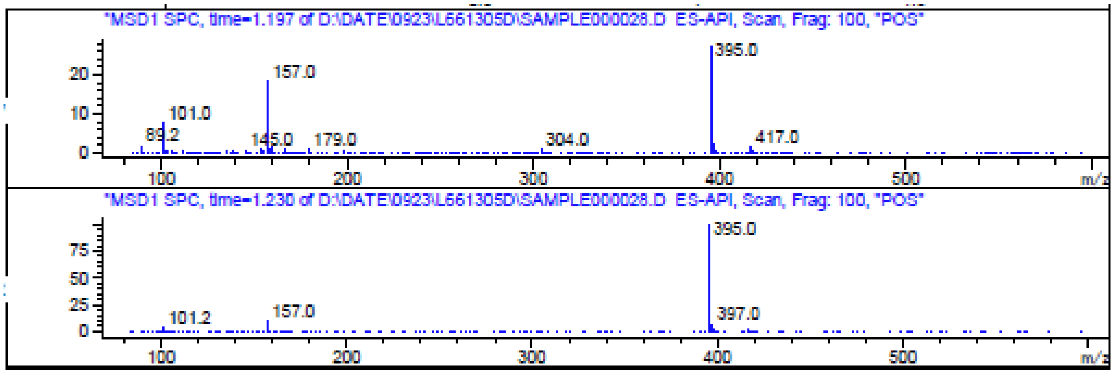

Fig. (S30). LC/MS spectrum of compound **3e**. RT = 1.197 min, 10.16 %; RT = 1.230 min, 85.28 %

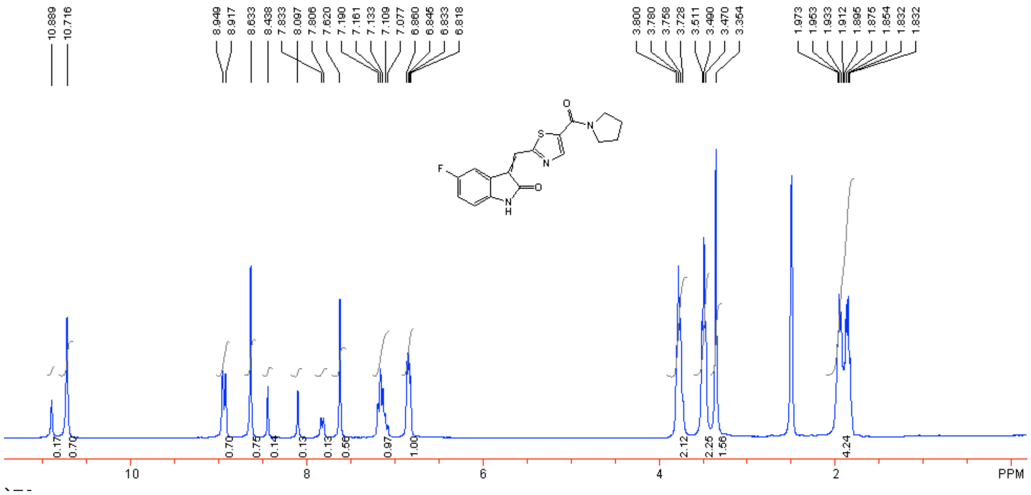

Fig. (S31). ^{1}H-NMR spectrum (DMSO-d_6) of compound **3f**.

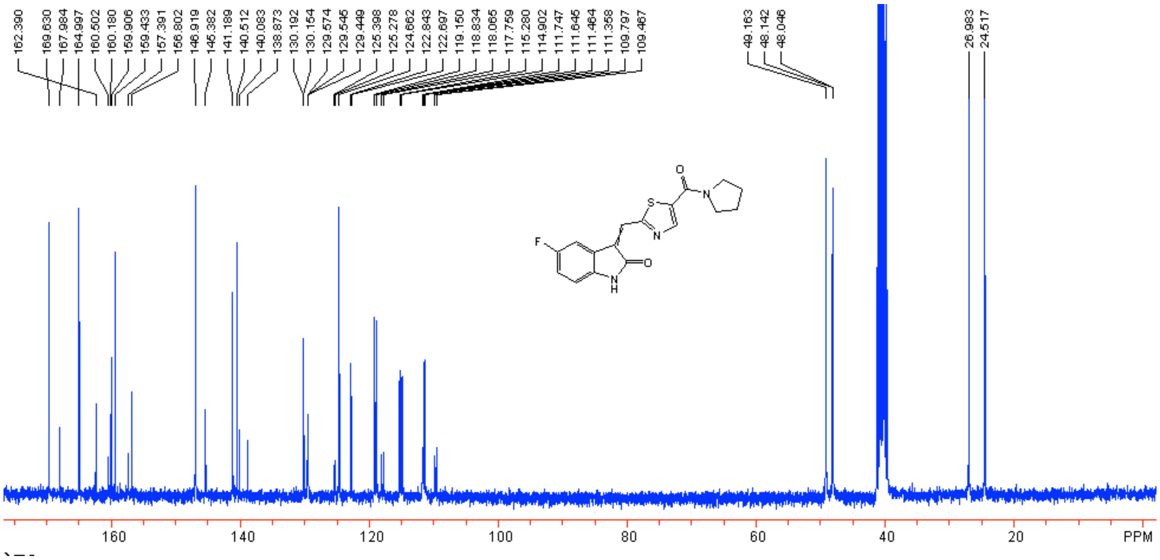

Fig. (S32). ^{13}C-NMR spectrum (DMSO-d_6) of compound **3f**.

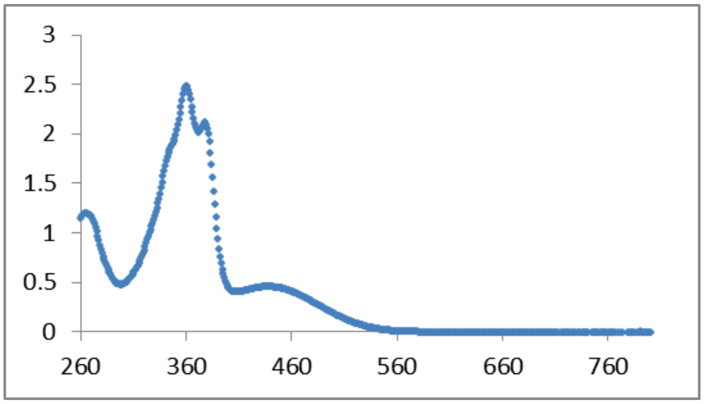

Fig. (S33). UV/Vis spectrum (DMSO) of compound **3f**.

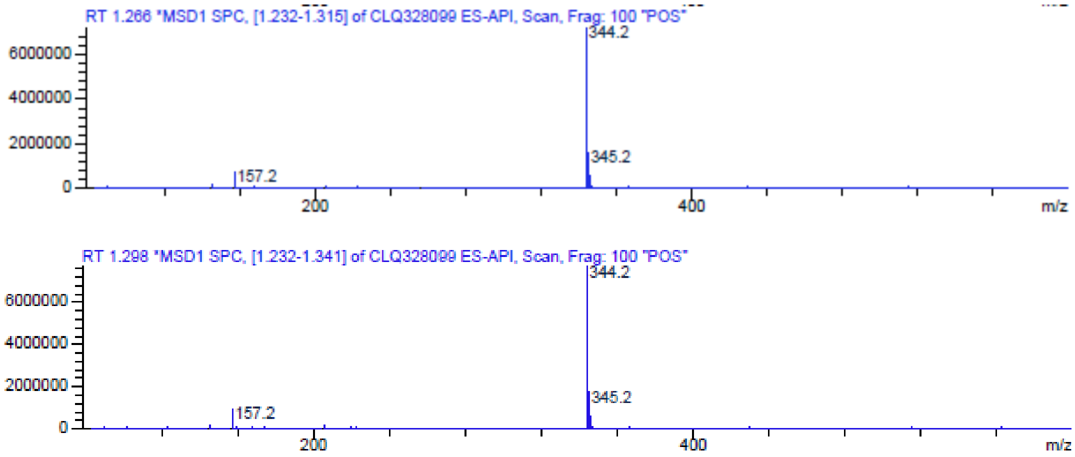

#	RT	DAD1A	DAD1B	MSD1	MSD2	ELSD	MSD1 ions	MSD1 rt	MSD2 ions	MSD2 rt	Info
	1.250	7.9%	6.6%	1.5%	13.5%	---	515.2(100)	1.266	342.2(100)	1.264	
	1.285	85.8%	88.3%	82.7%	71.1%	100.0%	344.2(100)	1.298	342.2(100)	1.297	

Fig. (S34). LC/MS spectrum of compound **3f**.

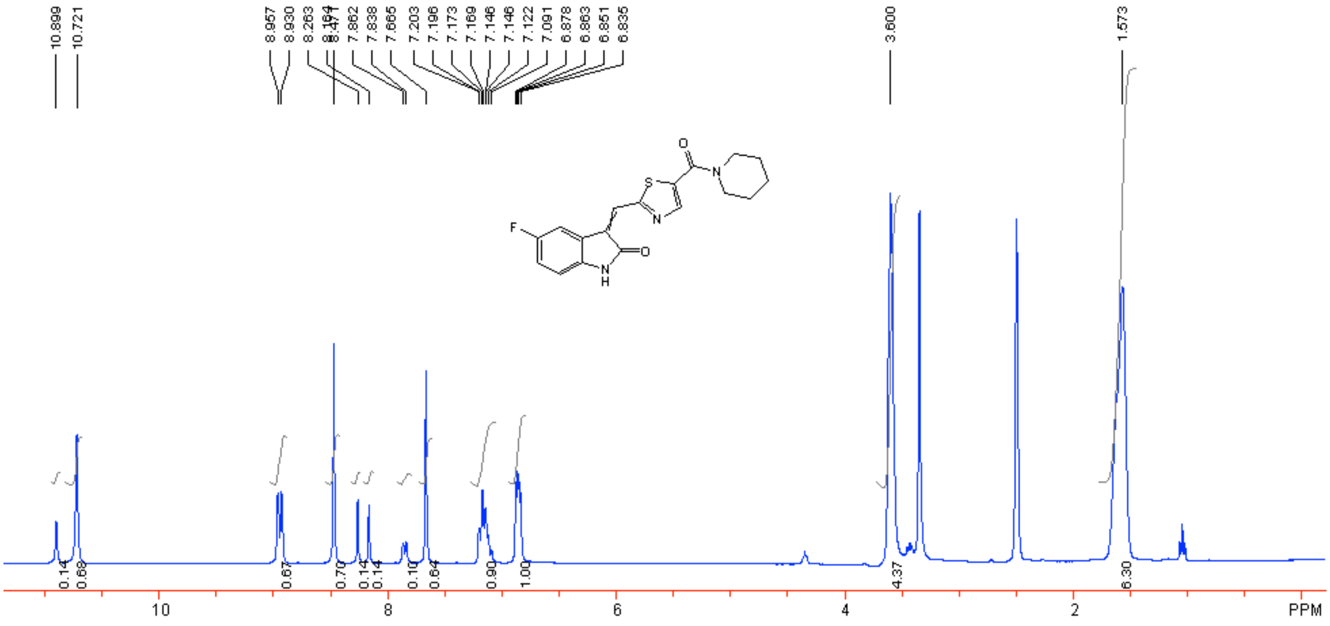

Fig. (S35). ^{1}H-NMR spectrum (DMSO-d_6) of compound **3g**.

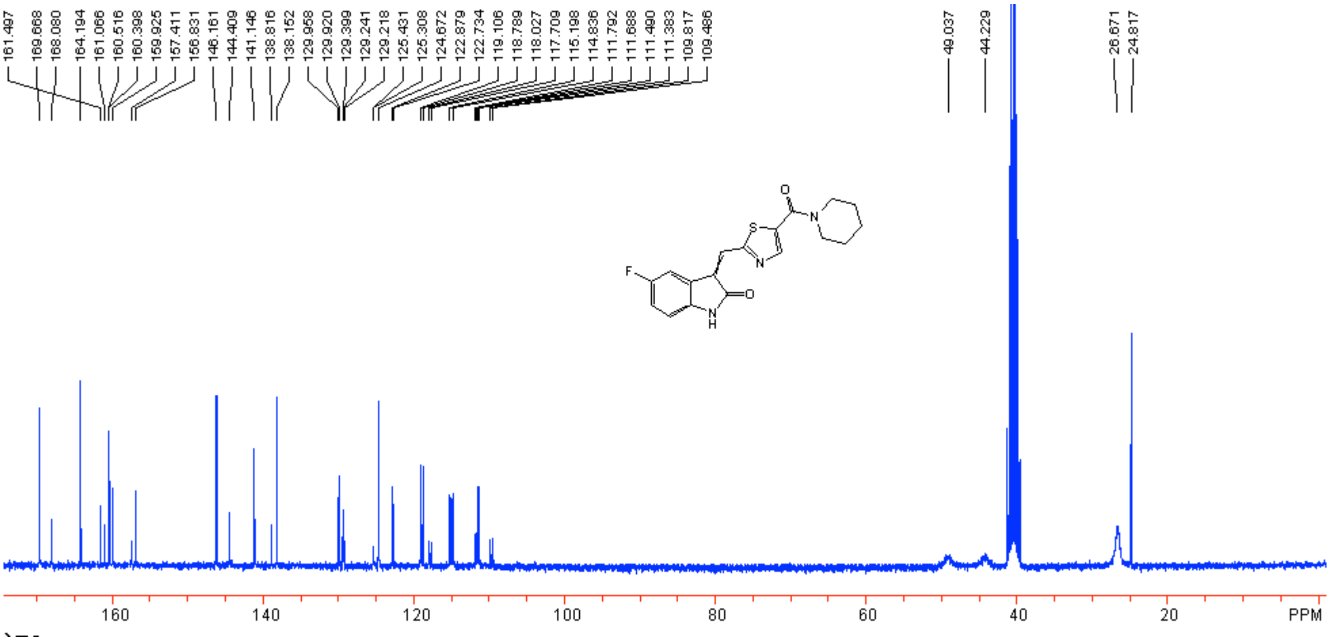

Fig. (S36). ^{13}C-NMR spectrum (DMSO-d_6) of compound **3g**.

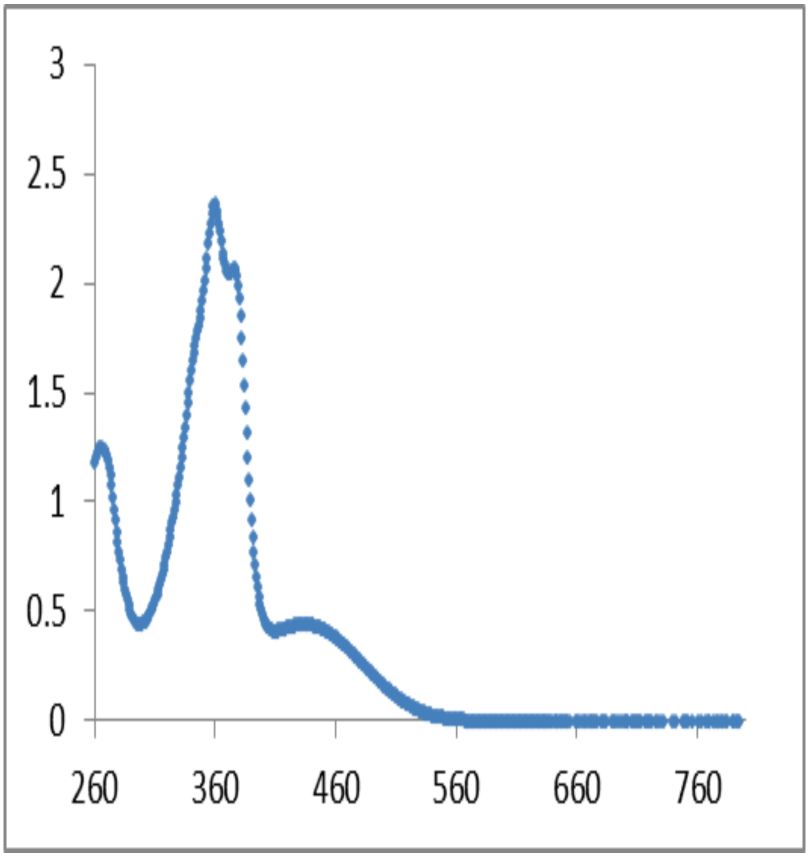

Fig. (S37). UV/Vis spectrum (DMSO) of compound **3g**.

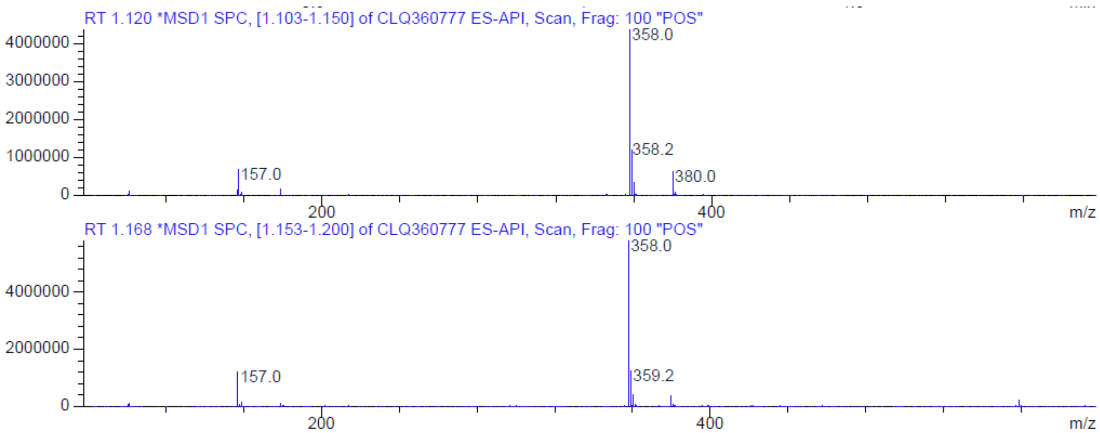

#	RT	DAD1A	DAD1B	MSD1	MSD2	ELSD	MSD1 ions	MSD1 rt	MSD2 ions	MSD2 rt	Info
1	1.111	22.8%	19.0%	50.2%	30.2%	---	358.0(91),380.0(9)	1.120	356.0(95),423.8(5)	1.118	
2	1.159	77.2%	81.0%	49.8%	69.8%	100.0%	358.0(95),380.0(5)	1.168	356.0(100)	1.169	

Fig. (S38). LC/MS spectrum of compound **3g**.

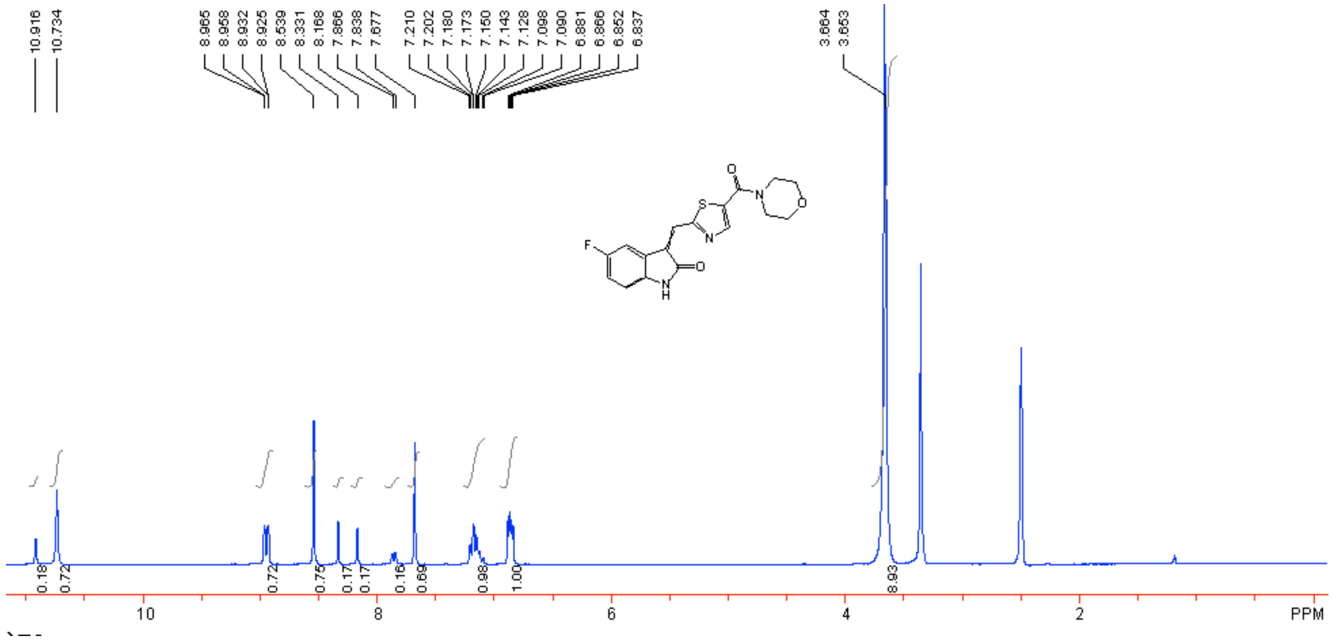

Fig. (S39). ^{1}H-NMR spectrum (DMSO-d_6) of compound **3h**.

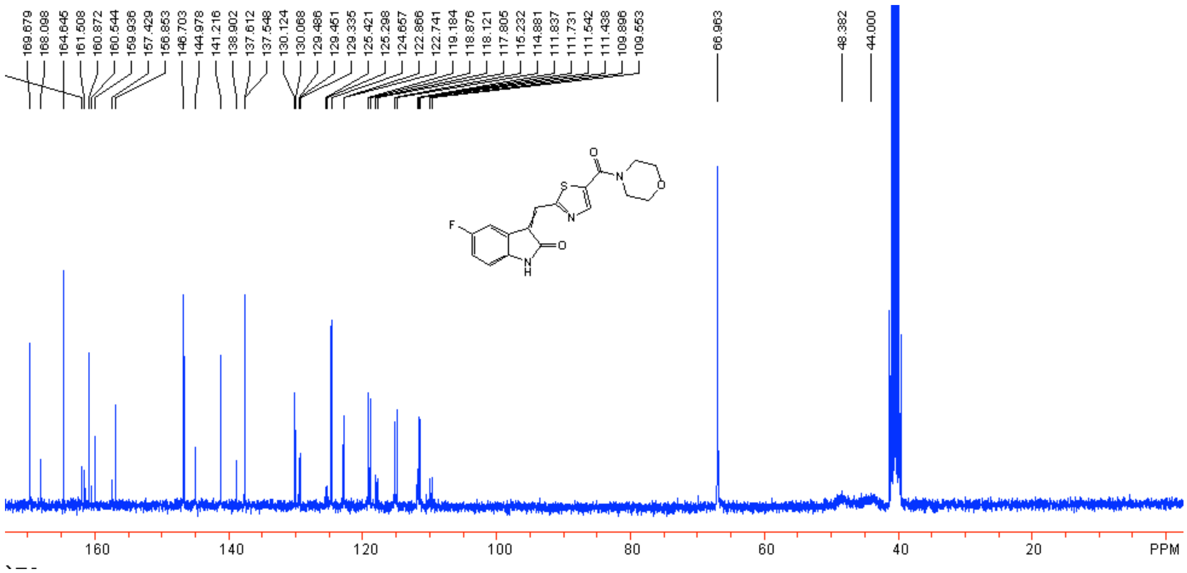

Fig. (S40). ^{13}C-NMR spectrum (DMSO-d_6) of compound **3h**.

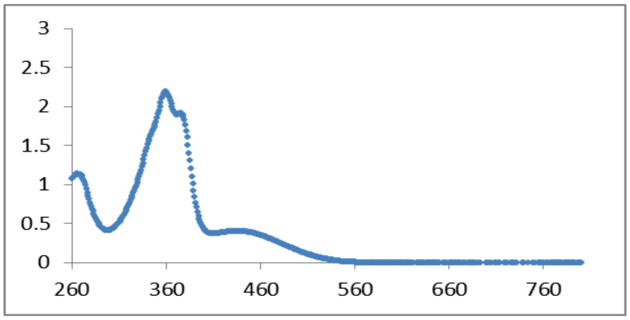

Fig. (S41). UV/Vis spectrum (DMSO) of compound **3h**.

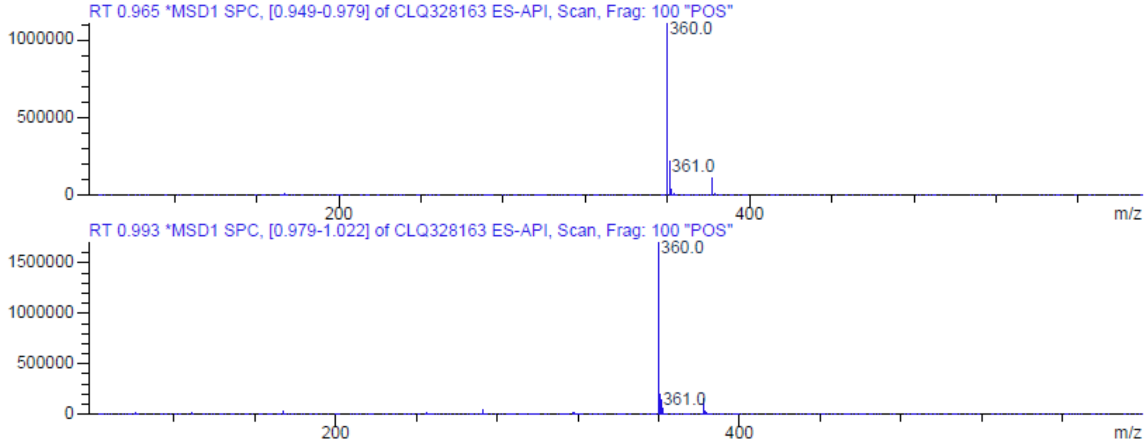

#	RT	DAD1A	DAD1B	MSD1	MSD2	ELSD	MSD1 ions	MSD1 rt	MSD2 ions	MSD2 rt	Info
1	0.958	20.5%	16.7%	37.1%	32.9%	---	360.0(100)	0.965	358.2(100)	0.965	
2	0.984	79.5%	83.3%	62.9%	67.1%	100.0%	360.0(90),382.0(8),273.0(2)	0.993	358.2(100)	0.992	

Fig. (S42). LC/MS spectrum of compound **3h**.

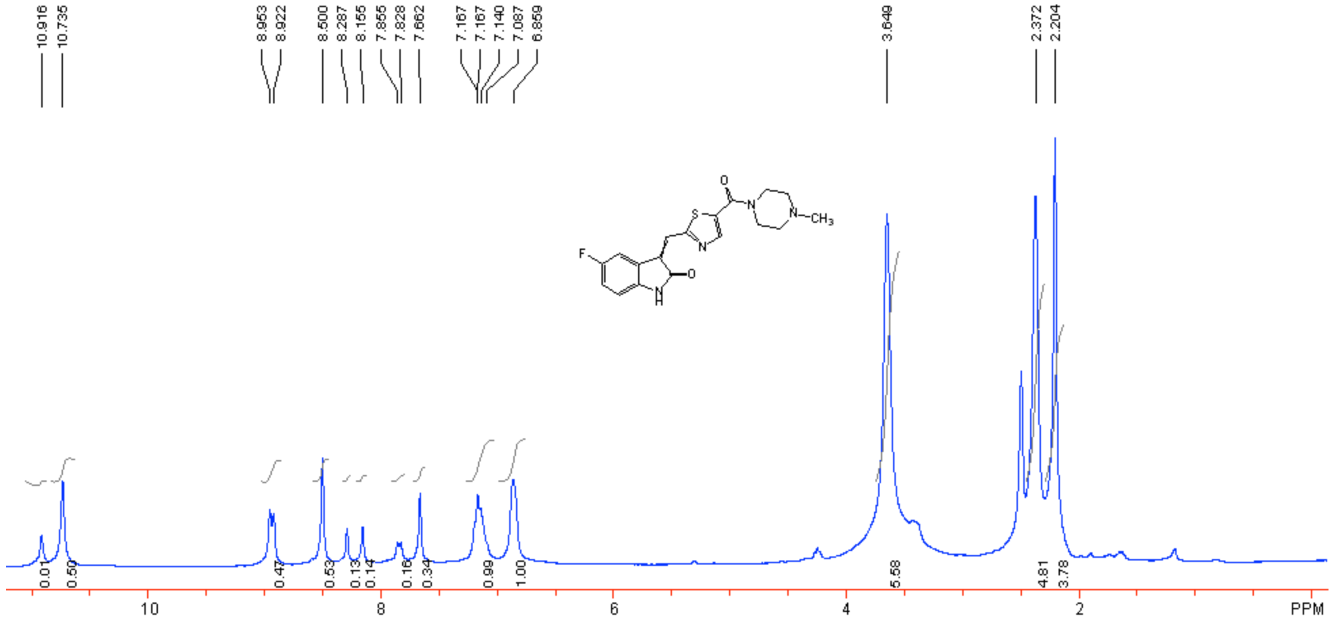

Fig. (S43). ^{1}H-NMR spectrum (DMSO-d_6) of compound **3i**.

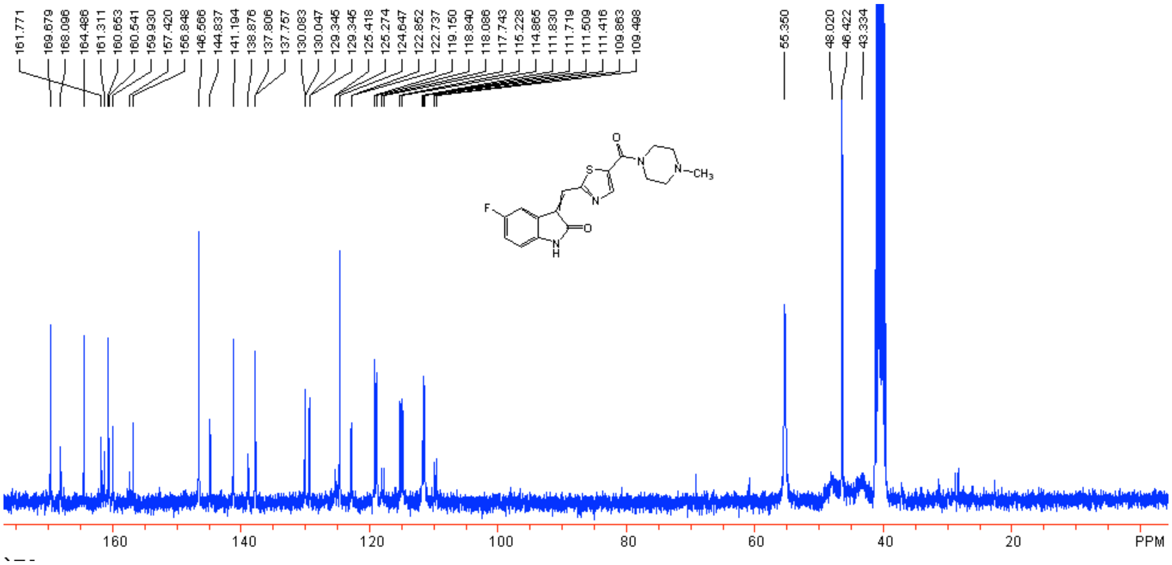

Fig. (S44). ^{13}C-NMR spectrum (DMSO-d_6) of compound **3i**.

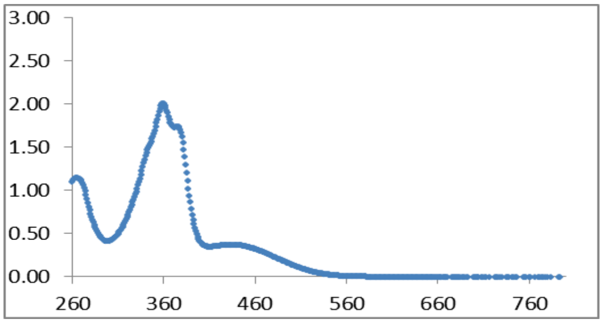

Fig. (S45). UV/Vis spectrum (DMSO) of compound **3i**.

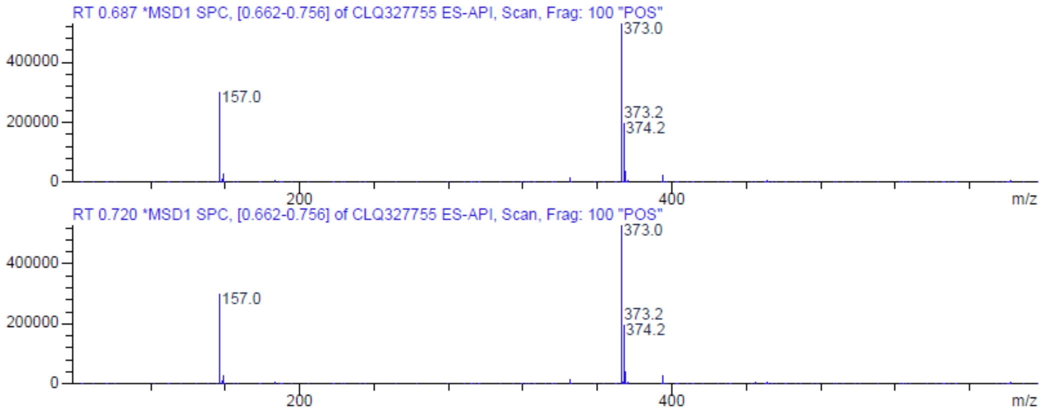

#	RT	DAD1A	DAD1B	MSD1	MSD2	ELSD	MSD1 ions	MSD1 rt	MSD2 ions	MSD2 rt	Info
1	0.676	77.7%	81.0%	82.6%	1.5%	100.0%	373.0(100)	0.687	428.8(100)	0.685	
2	0.707	22.3%	19.0%	3.0%	98.5%	---	395.0(100)	0.720	371.0(70),407.0(27),369.4(2)	0.716	

Fig. (S46). LC/MS spectrum of compound **3i**.

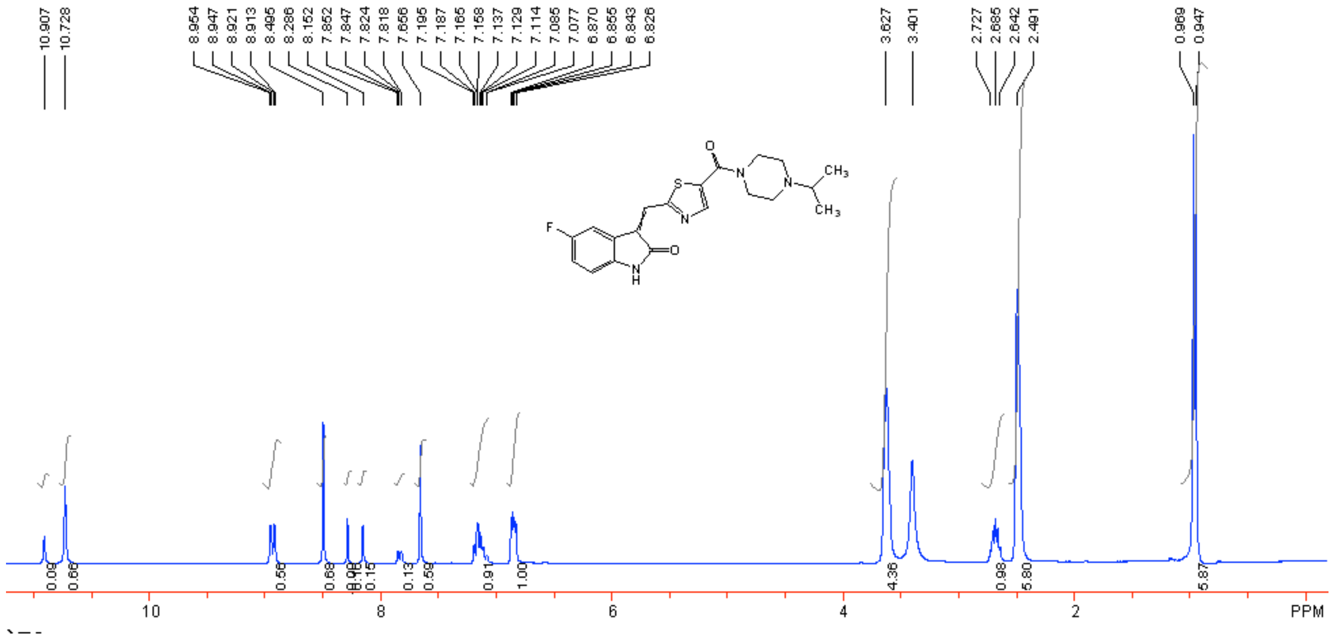

Fig. (S47). ^{1}H-NMR spectrum (DMSO-d_6) of compound **3j**.

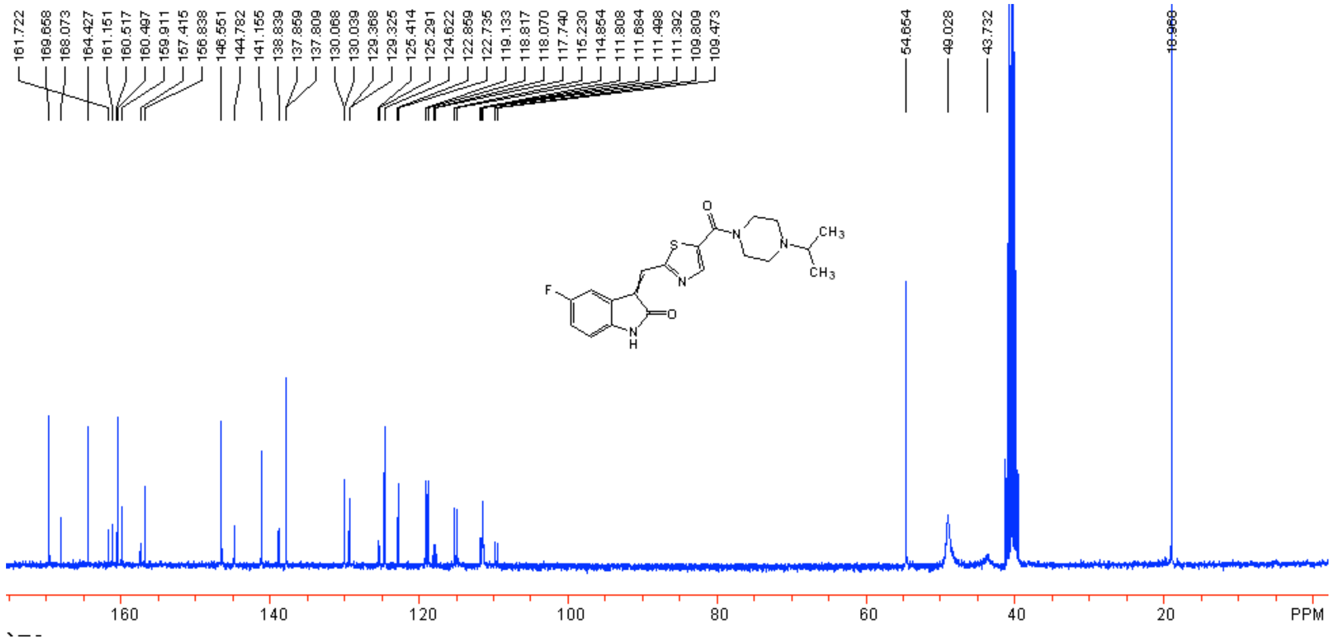

Fig. (S48). ^{13}C-NMR spectrum (DMSO-d_6) of compound **3j**.

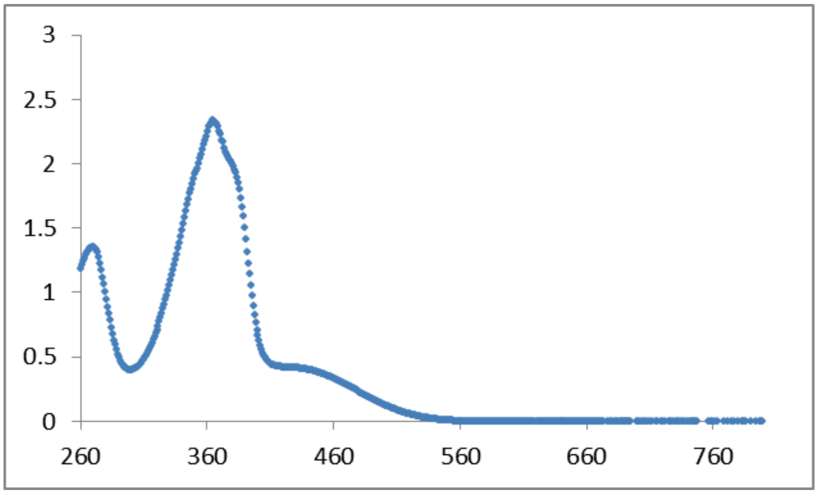

Fig. (S49). UV/Vis spectrum (DMSO) of compound **3j**.

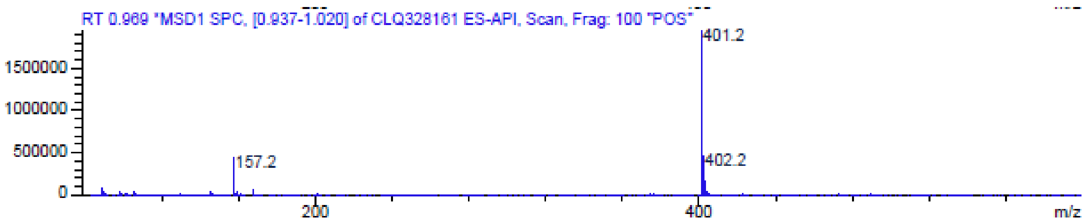

#	RT	DAD1A	DAD1B	MSD1	MSD2	ELSD	MSD1 ions	MSD1 rt	MSD2 ions	MSD2 rt	Info
	0.952	73.4%	69.4%	96.2%	2.0%	100.0%	401.2(100)	0.969	443.4(100)	0.967	

Fig. (S50). LC/MS spectrum of compound **3j**.

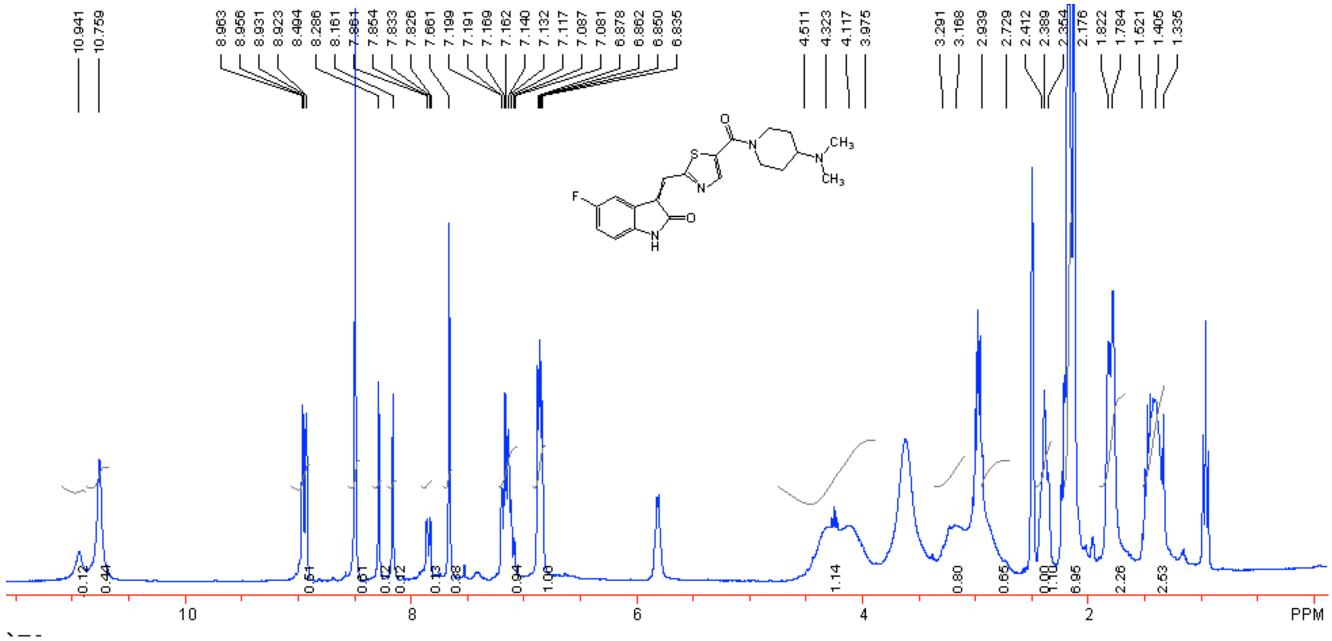

Fig. (S51). ^{1}H-NMR spectrum (DMSO-d_6) of compound **3k**.

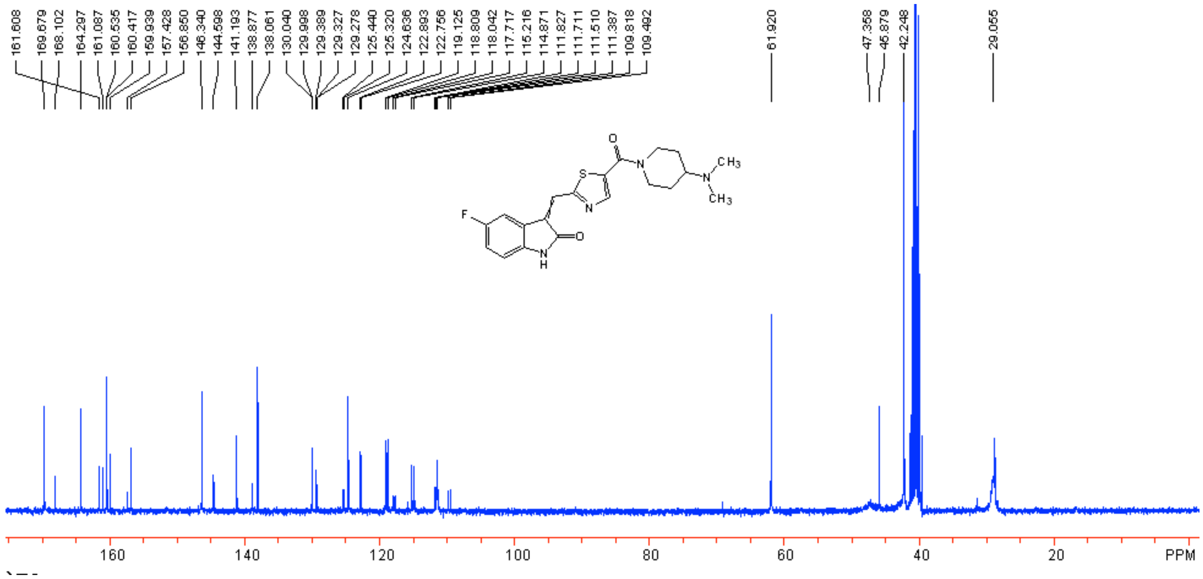

Fig. (S52). ^{13}C-NMR spectrum (DMSO-d$_6$) of compound **3k**.

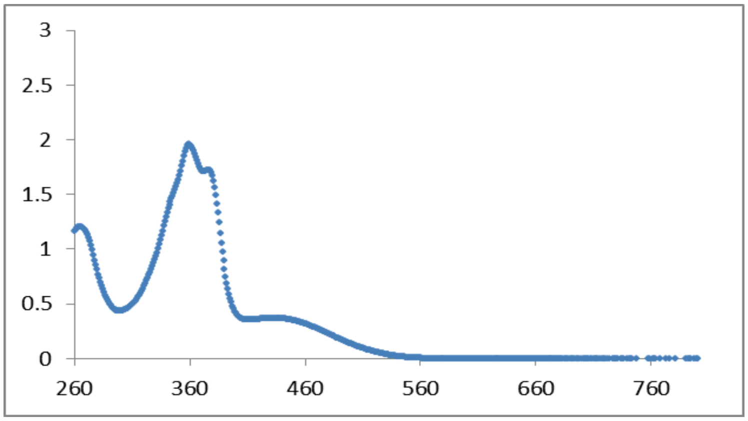

Fig. (S53). UV/Vis spectrum (DMSO) of compound **3k**.

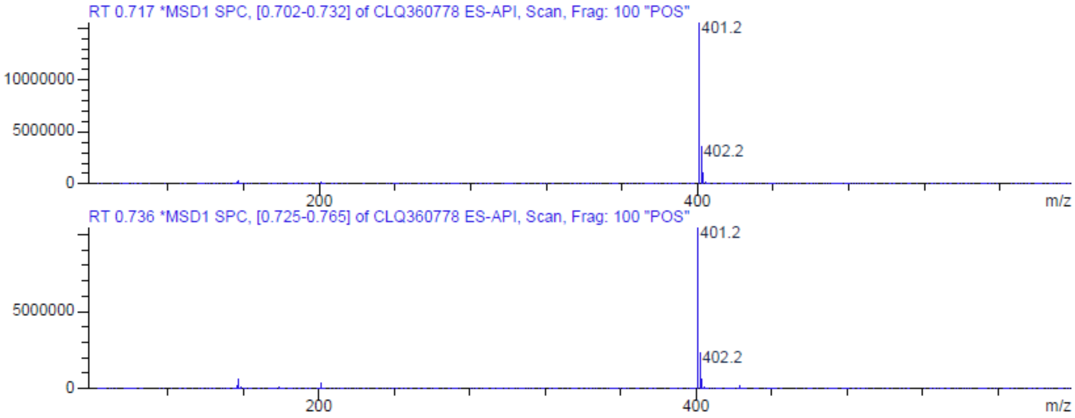

#	RT	DAD1A	DAD1B	MSD1	MSD2	ELSD	MSD1 ions	MSD1 rt	MSD2 ions	MSD2 rt	Info
1	0.708	74.4%	78.2%	64.9%	63.1%	100.0%	401.2(100)	0.717	399.0(100)	0.718	
2	0.727	25.6%	21.8%	35.1%	36.9%	---	401.2(96),201.0(4)	0.736	399.0(100)	0.735	

Fig. (S54). LC/MS spectrum of compound **3k**.

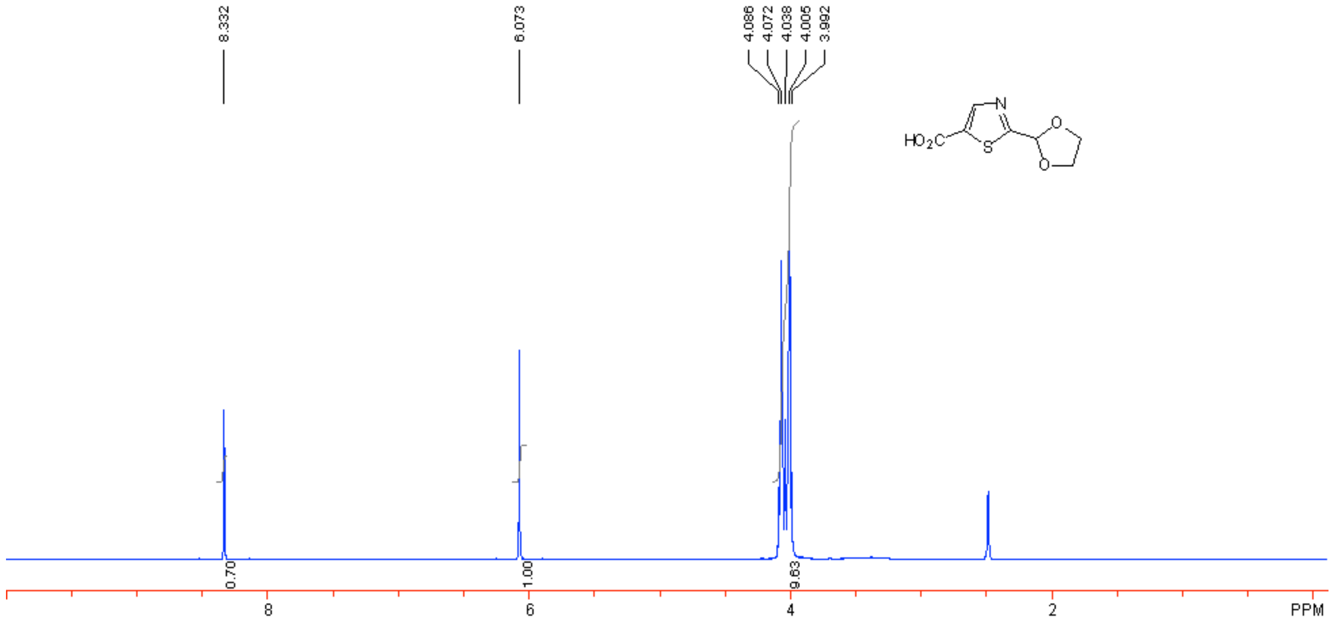

Fig. (S55). ^{1}H-NMR spectrum (DMSO-d$_6$) of compound **1**.

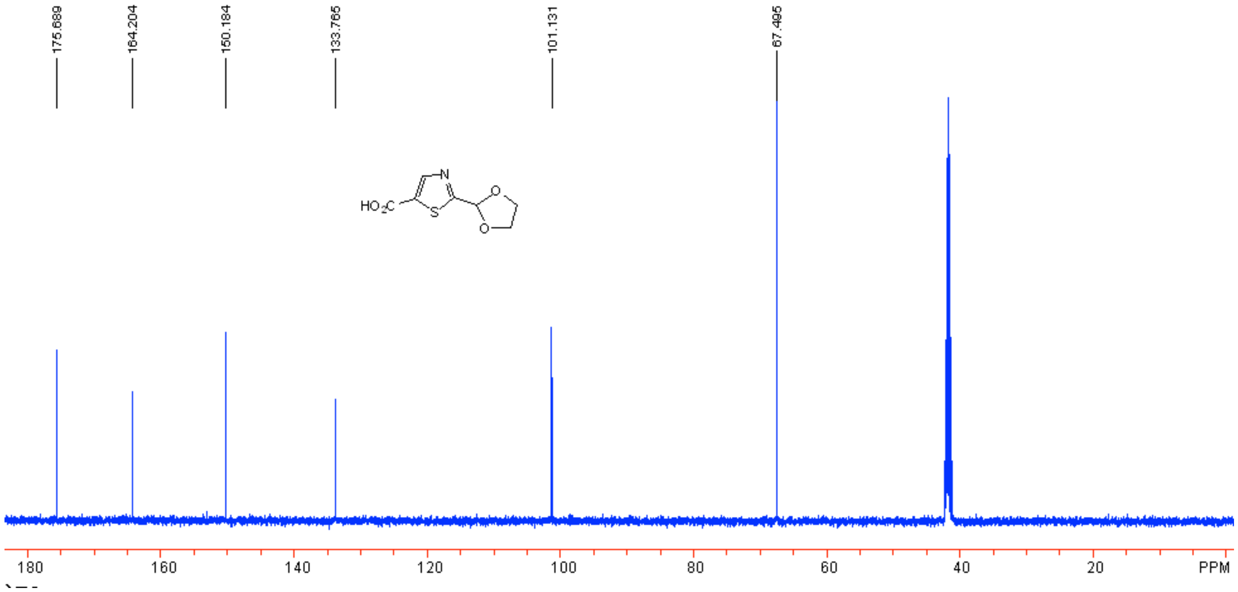

Fig. (S56). ^{13}C-NMR spectrum (DMSO-d$_6$) of compound **1.**

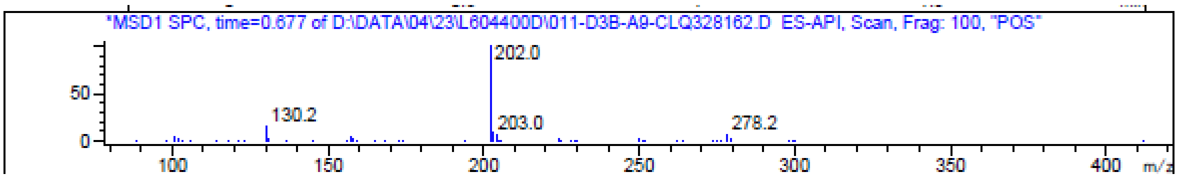

Fig. (S57). LC/MS spectrum of compound **1**, RT = 0.677 min.
